# A mild catalytic system for radical conjugate addition of nitrogen heterocycles†
†Electronic supplementary information (ESI) available. See DOI: 10.1039/c7sc00243b



**DOI:** 10.1039/c7sc00243b

**Published:** 2017-02-13

**Authors:** R. A. Aycock, H. Wang, N. T. Jui

**Affiliations:** a Department of Chemistry , Winship Cancer Institute , Emory University , Atlanta , GA 30322 , USA . Email: njui@emory.edu

## Abstract

The direct addition of pyridine and diazine units to electron-poor alkenes has been achieved *via* a redox radical mechanism that is enabled by limiting the effective concentration of the hydrogen-atom source. The described method is tolerant of acidic functional groups and is generally applicable to the union of a wide range of Michael acceptors and 6-membered heterocyclic halides.

## Abstract 2

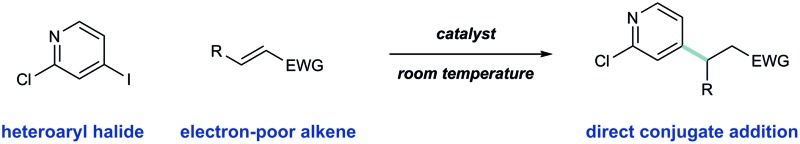
A catalytic redox system for the direct conjugate addition of pyridines and diazines to Michael acceptors has been developed.

## Introduction

**Fig. 1:**
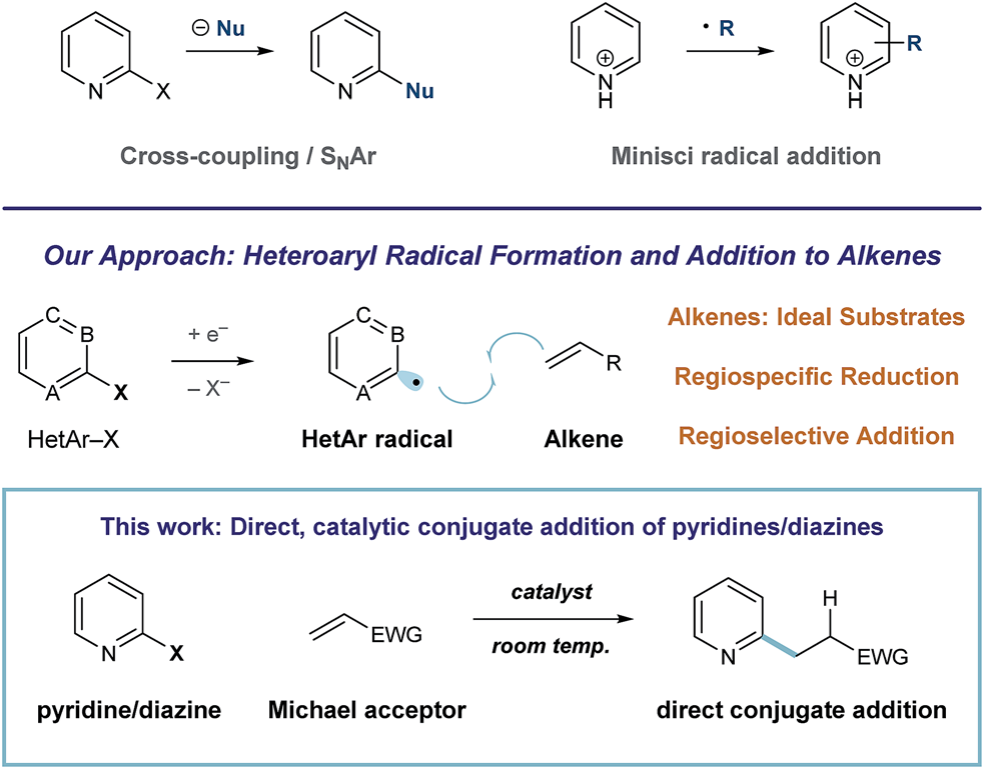
General strategies for the synthesis of complex heteroarenes.

Pyridines and diazines are critical structural elements in many biologically active small molecules^1^ and, as a result, significant research effort has been devoted to their preparation.^2^ In addition to *de novo* heterocycle assembly, a number of powerful methods exist for the functionalization of these heteroarenes. For example, Minisci radical addition is a direct and effective synthetic approach to the preparation of alkyl pyridines and diazines,^3^ however, the regiochemical outcome of these processes is largely dictated by the inherent reactivity of a given substrate (or substrate class).^4,5^ Catalytic coupling processes of halogenated heteroarene substrates with alkyl metals^6^ and, more recently, alkyl halides^7^ have been developed for the direct synthesis of alkylated heteroaromatics. We recently became interested in developing an alternative approach to complex pyridine and diazine synthesis *via* direct union of these heteroaromatic units with alkenes. More specifically, we envision a general strategy for programmed, regiospecific heteroarene activation that functions through heteroaryl radical intermediates. In contrast to alkyl radicals, aryl radical species effectively engage a wide range of unsaturated substrates.^8^ Consequently, mild conditions that deliver these reactive intermediates could enable the development of many discrete, practical processes for complex pyridine and diazine synthesis (Fig. 1).

Here, we describe the development of a catalytic system for heteroaryl radical formation and direct coupling with electron-deficient alkenes, a reductive Meerwein arylation^9^ process (illustrated in Fig. 1). Conjugate addition is a highly utilized strategic disconnection, but direct Michael addition of 6-membered nitrogen heterocycles remains challenging.

Because pyridines are weakly nucleophilic, they require activation to effectively add to alkenes. Miyaura demonstrated that rhodium-catalyzed asymmetric conjugate addition of *o*-methoxy pyridylboronic acids is efficient, but analogous coupling of the parent 2-pyridyl boronic acid (devoid of the electron-donating blocking group) was unsuccessful.^10^ A 2-pyridylboronate substrate was utilized in Akita's aryl radical conjugate addition system, based on photoredox arylboronate oxidation, to give the alkylpyridine in low yield (24%).^11^ Nilsson described an effective system for pyridylcuprate Michael addition,^12^ but Gilman reagents are extremely acid-sensitive, which limits their utility in complex molecule synthesis. Additionally, none of these strategies have demonstrated the ability to accomplish diazine conjugate addition. Condon described a Ni-catalyzed reductive Heck process of heteroaryl halides using electrochemistry, but this system was limited to monosubstituted alkenes.^13^ Our strategy for heteroarene activation is based on single-electron reduction and fragmentation of heteroaryl halides to regiospecifically afford the corresponding radical species.^14^ Aryl radical addition to electron-poor alkenes is facile,^15^ and this would offer a general alternative to pyridine and diazine conjugate addition that operates at room temperature and is tolerant of acidic functional groups.

Aryl radicals are indispensable intermediates in organic synthesis, and redox processes of arenediazonium salts^8,9,16^ or arylboronic acids^17^ are reliable methods for their formation. However, these strategies are limited in the context of pyridine or diazine-based radical generation, due to the instability of the requisite heteroaryl-diazonium^18*a*^ or -boronic acid reagents.^18*b*^ Tin-mediated halogen abstraction delivers (hetero)aryl radical intermediates^19^ but intermolecular alkene coupling reactions are challenging within this manifold because hydrogen atom transfer (HAT) to aryl radicals by tin-hydrides is rapid.^20^ Our method for reductive aryl radical generation involves photoinduced electron transfer. This mode of radical formation, first described by Beckwith,^21^ has been recently employed by Stephenson,^22^ Read de Alaniz and Hawker,^23^ Weaver,^24^ and König^25^ to accomplish hydrodehalogenation and a range of C–C bond-formations, mediated by photoredox catalysts.^26^ Notably, Weaver detailed conditions for the reductive coupling of simple alkenes with 2-haloazoles, polyfluorinated (hetero)aromatics, and a single example of an electron-deficient pyrimidine.^24^ The successful translation of this radical strategy to heteroaryl conjugate addition could streamline the invention of bioactive small molecules.

## Results and discussion

To assess the feasibility of our design, we studied the radical coupling of 2-iodopyridine (**1**) with the alkylidene malonate **2** (3.0 equivalents). We found that 1 mol% of the iridium-based photoredox catalyst Ir[dF(CF_3_)ppy]_2_dtbbpy·PF_6_ (among others)^27^ is capable of reductive 2-pyridyl radical formation under irradiation with a commercially available blue LED.

Alkylamines are effective stoichiometric reductants in photoredox processes, and their use in this context afforded the desired radical conjugate addition (RCA) product **3**, albeit in low yield (Table 1, entries 1 and 2). While tributylammonium formate (the reductant used by Weaver for 2-bromoazole radical formation,^24a–c^ entry 3) was similar in efficiency to the free base, the use of Hantzsch ester (HEH) delivered **3** in 50% yield (entry 4). In this system, HEH presumably donates an H-atom (to the intermediate radical adduct) and an electron to maintain redox neutrality. We found that the yield of this process was uniformly improved when aqueous solvent mixtures were employed (entries 5–8), and the use of 25% (v/v) H_2_O/DMSO afforded the desired product in 96% yield (entry 8).

**Table 1:** Optimization of conditions for heteroaryl radical conjugate addition^*a*^

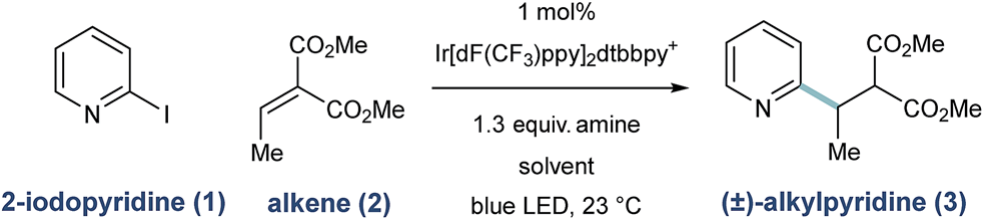			
Entry	Amine	Solvent	Yield of **3** ^*b*^
1^*c*^	i-Pr_2_NEt	CH_3_CN	12%
2^*c*^	NBu_3_	CH_3_CN	29%
3^*c*^	NBu_3_·HCO_2_H	CH_3_CN	28%
4	Hantzsch ester	CH_3_CN	50%
5	Hantzsch ester	25% H_2_O/CH_3_CN	80%
6	Hantzsch ester	25% H_2_O/DMF	75%
7	Hantzsch ester	25% H_2_O/MeOH	78%
8	Hantzsch ester	25% H_2_O/DMSO	96%

^*a*^Reaction conditions: 2-iodopyridine (1.0 equiv.), dimethyl ethylidene malonate (3.0 equiv.), Ir[dF(CF_3_)ppy]_2_dtbbpy·PF_6_ (1.0 mol%), amine (1.3 equiv.), 25% H_2_O/DMSO (10 mL mmol^–1^ heteroarene), blue light, 23 °C, 18 h.

^*b*^Yields determined by GC using dodecane as internal standard.

^*c*^With 3.0 equiv. amine.

The scope of this heteroarene conjugate addition protocol was then investigated. As shown in Table 2, these mild redox conditions enable the union of 2-pyridyl radical with an array of Michael acceptors with good efficiency. Cyclic ketones and crotononitrile react to give the corresponding pyridines in good yield (entries 1–3, 72–85% yield). Enoates with α-phenyl or α-chloro substitution are effective radical acceptors in this system (entries 4 and 5, ≥84% yield), giving rise to the complex esters in 4 : 3 and 4 : 1 dr, respectively. These radical conditions tolerate N–H and O–H bonds, as exemplified by the effective coupling of carboxylic acid, benzyl amide, and primary alcohol containing substrates (entries 6, 7, 14; 68–74% yield). Steric congestion on the alkene currently diminishes reactivity in this protocol, as demonstrated by entries 10–12; β-methyl, -isobutyl, and -isopropyl substitution results in formation of the desired malonate products in decreasing order (89%, 67%, and 50% yield respectively). A tryptophan-derived crotonamide was reacted with pyridyl radical to give the radical conjugate addition product in moderate yield (entry 15, 42% yield) as a 1 : 1 mixture of diastereomers. Although pyridine derivatives (like the products shown here) are effective radical traps, these conditions select for radical alkene addition. Additionally, phenyl rings (entries 4 and 7) and the indole function in the tryptophan product (entry 15) are unreactive toward aryl radical addition in this system.

**Table 2:** Heteroaryl radical conjugate addition: scope of the alkene coupling partner^*a*^

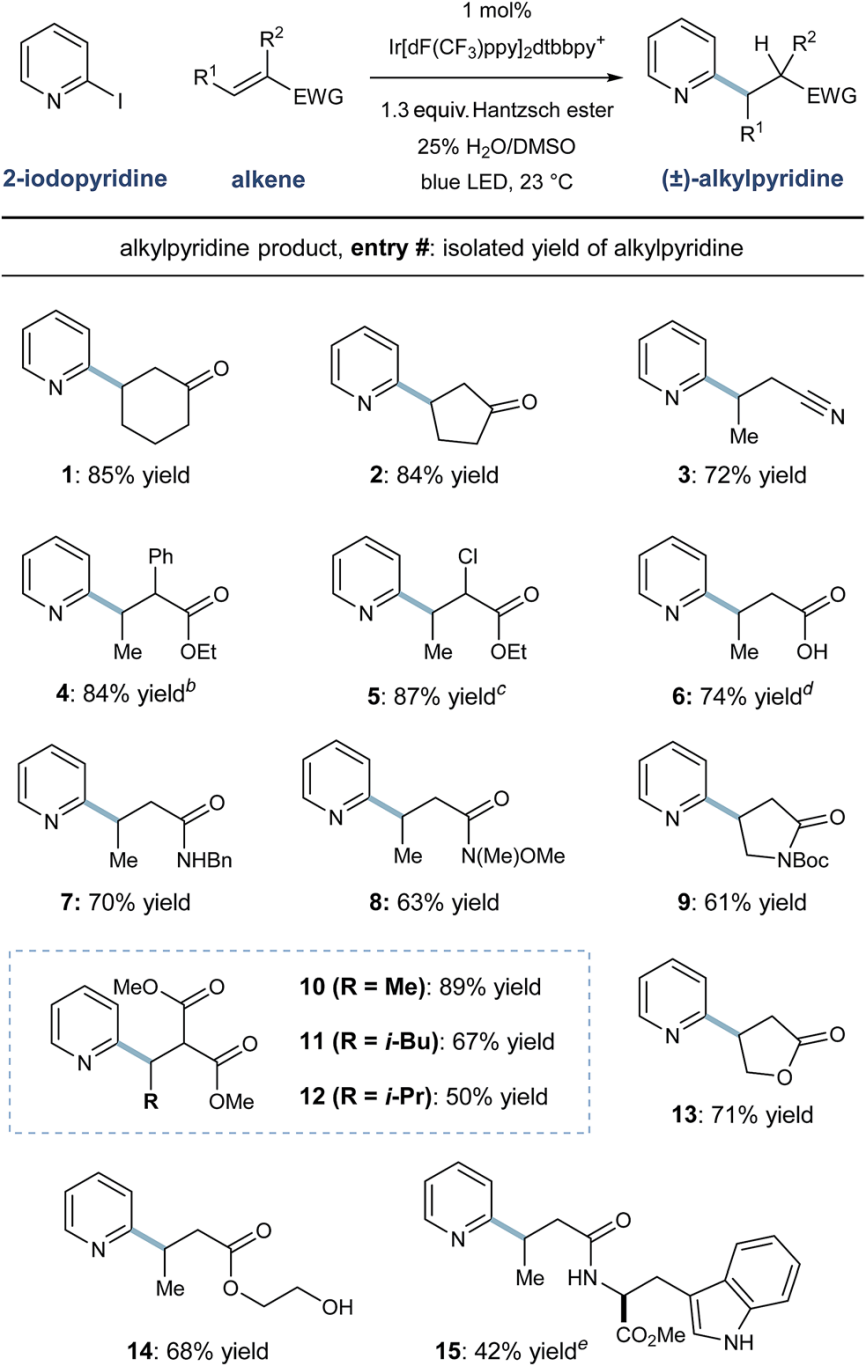

^*a*^Reaction conditions: 2-iodopyridine (1.0 equiv.), Michael acceptor (3.0 equiv.), Ir[dF(CF_3_)ppy]_2_dtbbpy·PF_6_ (1.0 mol%), Hantzsch ester (1.3 equiv.), 25% H_2_O/DMSO (10 mL mmol^–1^ heteroarene), blue light, 23 °C, 18 h.

^*b*^4 : 3 diastereomeric ratio (d.r.).

^*c*^4 : 1 d.r.

^*d*^Yield determined by ^1^H NMR using 1,3,5-trimethoxybenzene as internal standard.

^*e*^1 : 1 d.r.

We then evaluated the ability of our system to perform radical conjugate addition of other halogenated pyridines and diazines to ethyl crotonate (5 equivalents). Under standard conditions, a number of stable, commercially available heteroaryl iodides and bromides similarly function as radical precursors. As shown in Table 3, methyl substitution is tolerated at all positions of 2-pyridyl halides, and the corresponding products were formed in good yield (entries 1–4, 53–82%). Also competent are 3- and 4-iodopyridines (entries 5 and 6), and their use in this system provided the products in 52% and 48% yield, respectively.^28^ Because reductive radical formation is a regiospecific process, this approach allows for the predictable formation of alkylheterocycles as single regioisomers, including 3-alkylpyridines, which are not generally accessible *via* Minisci radical processes. Phenyl- and chloro-substitution is well tolerated to give the corresponding alkylpyridines in useful yield (entries 8, 10, 13; 53–61% yield).

**Table 3:** Photoredox radical conjugate addition: scope of halogenated heteroarenes^*a*^

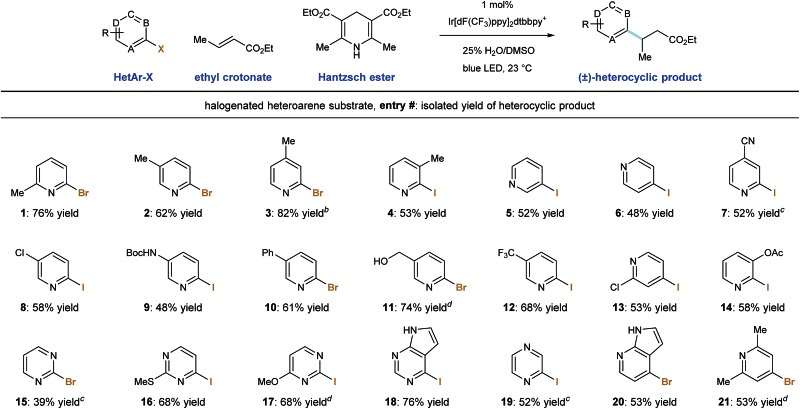

^*a*^Reaction conditions: halogenated heteroarene (1.0 equiv.), ethyl crotonate (5.0 equiv.), Ir[dF(CF_3_)ppy]_2_dtbbpy·PF_6_ (1.0 mol%), Hantzsch ester (1.3 equiv.), 25% H_2_O/DMSO (10 mL mmol^–1^ heteroarene), blue light, 23 °C, 18 h.

^*b*^2.0 mol% Ir[dF(CF_3_)ppy]_2_dtbbpy·PF_6_.

^*c*^Reaction conditions: heteroarene (1.0 equiv.), ethyl crotonate (3.0 equiv.), Ir[dF(CF_3_)ppy]_2_dtbbpy·PF_6_ (1.0 mol%), sodium formate (3.0 equiv.), 2,4,6-trimethylaniline (1.0 equiv.) DMSO (10 mL mmol^–1^ heteroarene), blue light, 23 °C, 18 h.

^*d*^Ir(ppy)_2_dtbbpy·PF_6_ (1.0 mol%) was used as catalyst.

Iodopyridines with the electron-withdrawing nitrile (entry 7) and trifluoromethyl (entry 12) groups were coupled with ethyl crotonate to give the corresponding products in 52% and 68% yield, respectively. Iodopyridines containing Boc-protected amine (entry 9) and benzyl alcohol (entry 11) functions were successfully coupled under these conditions without protecting groups that would be required to participate in anionic conjugate addition protocols (48% and 74% yield), respectively. Importantly, substituted iodopyrimidines also undergo radical formation and conjugate addition in moderate to good yield (entries 16–18, 68–76% yield). However, when iodopyrazine and 2-bromopyrimidine were used, the desired product was formed in trace amounts and a low mass balance was observed. We identified an alternate set of conditions involving the use of sodium formate (3.0 equiv.) and 2,4,6-trimethylaniline (1.0 equiv.) in DMSO solvent, which accomplished the radical conjugate addition of the parent pyrimidine (entry 15) and pyrazine (entry 19) elements in moderate yields (39% and 52%, respectively). While these alternate (sodium formate, trimethylaniline) conditions were effective in some cases, the use of Hantzsch ester as terminal reductant/hydrogen-atom source under aqueous conditions was more generally applicable. Finally, 2-iodopyrazine and 4-bromoazaindole were capable RCA substrates and the corresponding products were delivered in reasonable yield (entries 19 and 20, 52% and 53% yield, respectively).

**Scheme 1:**
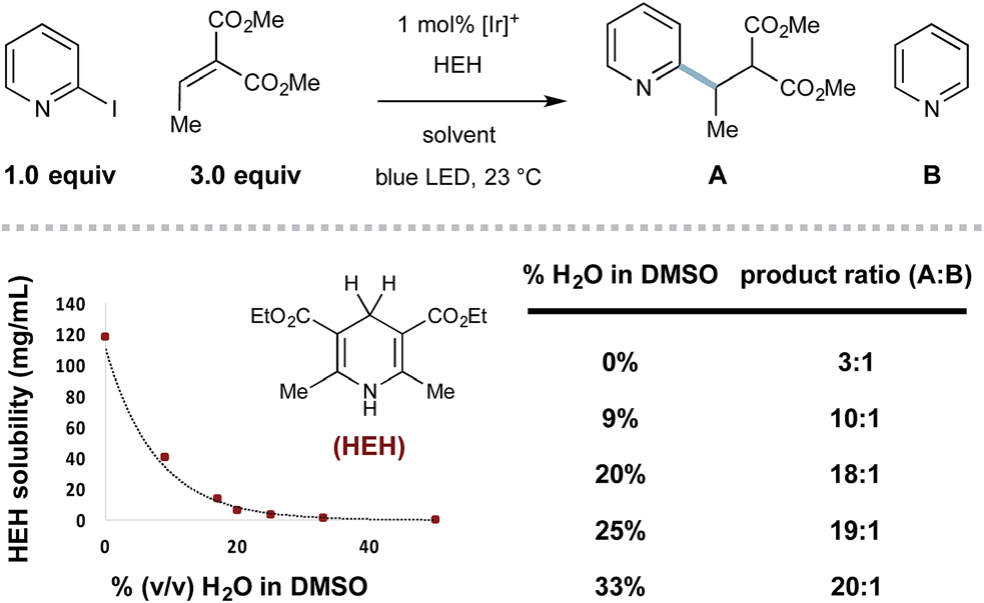
Limiting reductant (Hantzsch ester) solubility improves selectivity.

Throughout the course of this study, we observed that the described aqueous reaction conditions are uniquely effective for heteroaryl radical conjugate addition. Indeed, the use of aqueous solvents has improved the efficiency of other radical processes.^29^ In this system, we noticed that the introduction of water cosolvent resulted in heterogeneous reaction mixtures that became homogeneous with reaction progress. The solubility of HEH decreases precipitously with increasing amounts of water (shown in Scheme 1), and the selectivity for RCA *vs.* reduction is inversely proportional to HEH solubility (effective concentration), a principle first described by Stork.^30^ With the model 2-iodopyridine/ethylidene malonate coupling, the use of 33% (v/v) H_2_O/DMSO essentially eliminates the undesired hydrodehalogenation process, giving 20 : 1 selectivity (RCA product **A** : pyridine **B**).

To further exemplify the intermediacy of heteroaryl radical species in this system, we constructed the allyloxy iodopyridine **4**, understanding that reductive pyridyl radical formation would result in intramolecular addition to the pendant alkene. Under standard conditions, **4** underwent activation and radical cyclization to afford a mixture of bicyclic products (46% total yield, shown in eqn (1)). In addition to the expected product **5** (arising from 5-*exo*-trig cyclization), we observed preferential (2.5 : 1) formation of the 6-*endo* product **6**,^31^ and these data are consistent with the proposed radical nature of the described processes.1
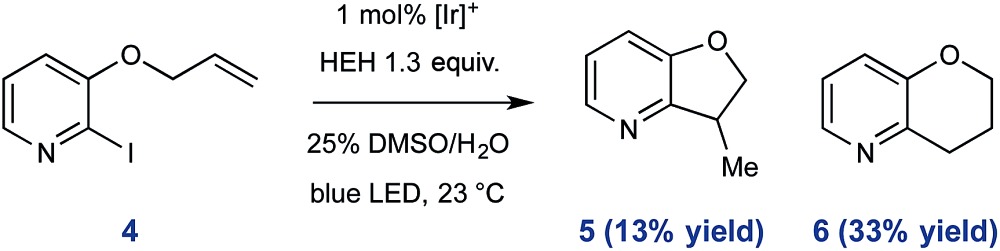



## Conclusions

In conclusion, we have designed a simple catalytic system that enables the general, regioselective coupling of pyridine and diazine units to electron-poor alkenes. This method utilizes simple alkenes, stable aryl radical precursors (many of the shown substrates are commercially available), and a commercial catalyst. We describe how limiting the effective concentration of Hantzsch ester enables the employment of these reactive species in the formation of carbon–carbon bonds for the preparation of a diverse array of heterocycle-containing products. Studies to further elucidate the operational mechanistic details of this process, as well as the development of related transformations are ongoing in our laboratory.
