# Supplementary material for: A mild catalytic system for radical conjugate addition of nitrogen heterocycles
Source: Chem Sci. 2017 Feb 13;8(4):3121–5. doi: 10.1039/c7sc00243b (PMC5412691; doi:10.1039/c7sc00243b)
Supplement: Supplementary file 1 [file SC-008-C7SC00243B-s001.pdf]

Electronic Supplementary Material (ESI) for Chemical Science.
This journal is © The Royal Society of Chemistry 2017

A Mild Catalytic System for Radical Conjugate Addition of Nitrogen Heterocycles

R. Adam Aycock‡, Hengbin Wang‡, Nathan T. Jui*

Department of Chemistry and Winship Cancer Institute, Emory University, Atlanta, GA 30322

Table of Contents

I. General Information…………………………………………………………. S-2
I-A. General Reagent Information……………………………………….. S-2
I-B. General Analytical Information……………………………………...S-2

II. General Procedures…………………………………………………………..S-3
II-A. General Procedure A……………………………………………….S-3
II-B. General Procedure B……………………………………………….. S-3

III. Optimization Details……………………………………………………….. S-4
III-A. Optimization Procedure A………………………………………… S-4
III-B. Optimization Procedure B…………………………………………S-4
III-C. Optimization Procedure C…………………………………………S-5
III-D. Optimization Procedure D…………………………………………S-5
III-E. Gas Chromatography Method Conditions…………………………S-5
III-F. Optimization Table……………………………………………….....S-6

IV. Preparation of Starting Materials…………………………………………S-6

V. Procedure and Characterization data………………………………………S-8

VI. Procedure for Determining Hantzsch Ester Solubility…………………... S-24

VII. Procedure for Intramolecular Cyclization………………………………. S-24

VIII. ^{1}H and ^{13}C NMR Spectra…………………………………………………..S-26

I. General Information

I-A. General Reagent Information:

All reactions were set up on the bench top and conducted under nitrogen atmosphere while subject to irradiation from blue LEDs (LEDwholesalers PAR38 Indoor Outdoor 16 Watt LED Flood Light Bulb, Blue; or PARsource PowerPAR LED Bulb-Blue 15 Watt/440 nm, available at www.eaglelight.com). Flash chromatography was carried out using Siliaflash® P60 silica gel obtained from Silicycle. Photo redox catalyst, $[Ir\{dF(CF_3)ppy\}_2(dtbbpy)]PF_6$, was prepared according to a literature procedure[1]. Halogenated heteroarenes were purchased from Aldrich Chemical Co., Alfa Aesar, Acros Organics, Combi-Blocks, or Oakwood Products and were used as received, with the exception of Table 3, entries 9 and 14. Table 3, entries 9 and 14 were prepared according to the procedure in section IV, Preparation of Starting Materials. Ethyl crotonate and alkenes for Table 2, entries 1, 2, 3, and 6 were purchased from Alfa Aesar and Acros Organics and were used as received. Alkenes for Table 2, entries 9 and 10 were purchased from Combi-Blocks and were used as received. Furan-2(5H)-one used for Table 2, entry 13 was purchased from Alfa Aesar and was used as received. Alkenes for Table 2, entries 4[2], 5[3], 7[4], 8[5], 11[6], 12[7] were prepared according to literature procedures. Alkenes for Table 2, entries 14 and 15 were prepared according to the designated procedures in section IV, Preparation of Starting Materials. DMSO was purified on a Pure Process Technologies solvent purification system. Reaction solvent was prepared by combining DMSO and tap water (3:1, v:v) which was degassed in a sidearm flask under weak vacuum while subject to sonication.

I-B. General Analytical Information:

All yields except refer to isolated yields with the exception of Table 2, entry 6, which was determined by NMR with 1,2,3-trimethoxybenzene as an internal standard on a Mercury 300 MHz spectrometer. New compounds were characterized by NMR, IR spectroscopy, HRMS, and melting point. NMR data were recorded on one of five spectrometers: INOVA 600 MHz, INOVA 500 MHz, VNMR 400 MHz, INOVA 400 MHz, or Mercury 300 MHz. Chemical shifts (δ) are internally referenced to residual protio solvent ($CDCl_3$: δ 7.26 ppm for ^1H NMR and 77.23 ppm for ^{13}C NMR; or Benzene-d_6: δ 7.15 for ^1H NMR and 128.4 ppm for ^{13}C NMR). IR spectra were obtained with a Thermo Scientific Nicolet iS10 Fourier transform infrared spectrophotometer. Mass spectrometry data were obtained from the

---

[1] Tellis, J. C.;Primer, D. N.; Molander, G. A. *Science.* **2014**, *345 (6195)*, 433-436.

[2]Mani, N. S.; Mapes, C. M.; Wu, J.; Deng, X.; Jones, T. K. *J. Org. Chem.* **2006**, *71 (13)*, 5039-5042.

[3]Brenna, E.; Gatti, F. G.; Manfredi, A.; Monti, D.; Parmeggiani, F. Organic Process Research & Development. *Org. Process Res. Dev.* **2012**, *16 (2)*, 262-268.

[4]Eriksson, J.; Aaberg, O.; Laangstroem, B. *Eur. J. Org. Chem.* **2007**, *3*, 455-461.

[5]Evans, D. A.; Song, H. J.; Fandrick, K. R. *Org. Lett.* **2006**, *8 (15)*, 3351-3354.

[6]Cardillo, G.; Gentilucci, L.; Gianotti, M.; Perciaccante, R.; Tolomelli, A. *J. Org. Chem.* **2001**, *66 (25)*, 8657-8660.

[7]Stevens, C. V.; Van Heecke, G.; Barbero, C.; Patora, K.; De Kimpe, N.; Verhe, R. *Synlett*. **2002**, *7*, 1089-1092.

Emory Mass Spectrometry Center. Melting point data was obtained with a Thomas Hoover Unimelt capillary melting point apparatus. Optimization data was obtained via gas chromatography with an Agilent Technologies 7890B Gas Chromatography system (flame-ionization detection) equipped with an Agilent Technologies 19091J-413 HP-5 column (30 m x 0.320 mm x 0.25 µm, 5 % phenyl methyl siloxane) and an Agilent Technologies G4513A autoinjector.

II. General Procedures for Coupling of Halogenated Heteroarene with Alkene

II-A. General Procedure A:

A 30-mL screw-top test tube equipped with a stir bar was charged with Hantzsch ester (1.3 equiv), [Ir{dF(CF_3)ppy}$_2$(dtbbpy)]PF_6 (1 mol %), alkene (3 or 5 equiv), and halogenated heteroarene (1 equiv). The tube was sealed with PTFE/silicon septum and connected to a vacuum line. The atmosphere was exchanged by applying vacuum and backfilling with N_2 (this process was conducted a total of three times). Under N_2 atmosphere, the tube was charged with degassed solvent (3:1 DMSO:H_2O, 10 mL/mmol heteroarene) by syringe. The resulting suspension was stirred under irradiation with blue LEDs for 18 hours or until consumption of the halogenated heteroarene was observed by gas chromatography. The reaction was quenched with saturated sodium bicarbonate solution (60 mL) and extracted with ethyl acetate (3 x 40 mL). The extracts were combined, dried over magnesium sulfate, filtered, and concentrated by rotary evaporation. The residue was purified by flash column chromatography using the indicated solvent mixture to afford the title compound.

II-B. General Procedure B:

A 30-mL screw-top test tube equipped with stir bar was charged with aryl halide (1 equiv), [Ir{dF(CF_3)ppy}$_2$(dtbbpy)]PF_6 1 mol %), sodium formate (3 equiv), 2,4,6-trimethylaniline (1 equiv), and alkene (3 equiv). The tube was sealed with a screw-cap with PTFE/silicon septum and connected to a vacuum line. The atmosphere was exchanged by applying vacuum and backfilling with N_2 (this process was conducted a total of three times). Under N_2 atmosphere, the tube was charged with degassed solvent (DMSO 10 mL/mmol heteroarene) by syringe. The resulting mixture was stirred under irradiation with blue LEDs overnight. The reaction was quenched with saturated sodium bicarbonate solution (60 mL) and extracted with ethyl acetate (3 x 40 mL). The extracts were combined, dried over magnesium sulfate, filtered and concentrated by rotary evaporation. The residue was purified by flash column chromatography using the indicated solvent mixture to afford the title compound.

III. Optimization Details

III-A. Optimization Procedure A (Entries 1-4):

A 15-mL screw-top test tube equipped with a stir bar was charged with Hantzsch ester (95 mg, 0.375 mmol, 1.3 equiv) [Ir{dF(CF_3)ppy}$_2$(dtbbpy)]PF_6 (2.8 mg, 0.0025 mmol, 1 mol %), dimethyl 2-ethylidenemalonate (120 mg, 0.75 mmol, 3 equiv), and 2-iodopyridine (52 mg, 0.25 mmol, 1 equiv). The tube was sealed with PTFE/silicon septum and connected to a vacuum line. The atmosphere was exchanged by applying vacuum and backfilling with N_2 (this process was conducted a total of three times). Under N_2 atmosphere, the tube was charged with amine or tributylammonium formate salt (NEt_3, NBu_3, (*i*-pr)$_2$NEt, or NBu_3•HCO_2H, 0.75mmol, 3 equiv) and degassed solvent (MeCN, 2.5 mL) by syringe. The resulting solution was stirred under irradiation with blue LEDs for 18 hours. The reaction was quenched with saturated sodium bicarbonate solution (10 mL) and extracted with ethyl acetate (5 x 5 mL). The extracts were combined and passed through a plug of silica which was flushed with acetone, and the solution was transferred to a 20-mL scintillation vial. An internal standard of dodecane (10 μL, 0.044 mmol) was delivered to the vial, and the contents were thoroughly mixed. A sample was analyzed by gas chromatography, and the integral values were used to calculate conversion, alkylpyridine (dimethyl 2-(1-(pyridin-2-yl)ethyl)malonate) yield, and hydrodehalogenation product (pyridine) yield.

III-B. Optimization Procedure B (Entry 5):

A 15-mL screw-top test tube equipped with a stir bar was charged with Hantzsch ester (83 mg, 0.325 mmol, 1.3 equiv), [Ir{dF(CF_3)ppy}$_2$(dtbbpy)]PF_6 (2.8 mg, 0.0025 mmol, 1 mol %), dimethyl 2-ethylidenemalonate (120 mg, 0.75 mmol, 3 equiv), and 2-iodopyridine (52 mg, 0.25 mmol, 1 equiv). The tube was sealed with PTFE/silicon septum and connected to a vacuum line. The atmosphere was exchanged by applying vacuum and backfilling with N_2 (this process was conducted a total of three times). Under N_2 atmosphere, the tube was charged with (*i*-pr)$_2$NEt (97 mg, 0.75mmol, 3 equiv) and degassed solvent (MeCN, 2.5 mL) by syringe. The resulting suspension was stirred under irradiation with blue LEDs for 18 hours. The reaction was quenched with saturated sodium bicarbonate solution (10 mL) and extracted with ethyl acetate (5 x 5 mL). The extracts were combined and passed through a plug of silica which was flushed with acetone, and the solution was transferred to a 20-mL scintillation vial. An internal standard of dodecane (10 μL, 0.044 mmol) was delivered to the vial, and the contents were thoroughly mixed. A sample was analyzed by gas chromatography, and the integral values were used to calculate conversion, alkylpyridine (dimethyl 2-(1-(pyridin-2-yl)ethyl)malonate) yield, and hydrodehalogenation product (pyridine) yield.

III-C. Optimization Procedure C (Entries 6-22):

A 15-mL screw-top test tube equipped with a stir bar was charged with Hantzsch ester (83 mg, 0.325 mmol, 1.3 equiv), [Ir{dF(CF_3)ppy}$_2$(dtbbpy)]PF_6 (2.8 mg, 0.0025 mmol, 1 mol %), dimethyl 2-ethylidenemalonate (120 mg, 0.75 mmol, 3 equiv), and 2-iodopyridine (52 mg, 0.25 mmol, 1 equiv). The tube was sealed with PTFE/silicon septum and connected to a vacuum line. The atmosphere was exchanged by applying vacuum and backfilling with N_2 (this process was conducted a total of three times). Under N_2 atmosphere, the tube was charged with degassed solvent (MeCN, DMSO, 3:1 DMF:H_2O, 3:1 MeOH:H_2O, 3:1 MeCN:H_2O, or 3:1 DMSO:H_2O, 2.5 mL) by syringe. The resulting suspension was stirred under irradiation with blue LEDs for 18 hours. The reaction was quenched with saturated sodium bicarbonate solution (10 mL) and extracted with ethyl acetate (5 x 5 mL). The extracts were combined and passed through a plug of silica which was flushed with acetone, and the solution was transferred to a 20-mL scintillation vial. An internal standard of dodecane (10 μL, 0.044 mmol) was delivered to the vial, and the contents were thoroughly mixed. A sample was analyzed by gas chromatography, and the integral values were used to calculate conversion, alkylpyridine (dimethyl 2-(1-(pyridin-2-yl)ethyl)malonate) yield, and hydrodehalogenation product (pyridine) yield.

III-D. Optimization Procedure D (Entry 22, air-exposed):

A 15-mL screw-top test tube equipped with a stir bar was charged with Hantzsch ester (83 mg, 0.325 mmol, 1.3 equiv), [Ir{dF(CF_3)ppy}$_2$(dtbbpy)]PF_6 (2.8 mg, 0.0025 mmol, 1 mol %), dimethyl 2-ethylidenemalonate (120 mg, 0.75 mmol, 3 equiv), and 2-iodopyridine (52 mg, 0.25 mmol, 1 equiv). The tube was charged with solvent (3:1 DMSO:H_2O, 2.5 mL) by syringe, and the tube was sealed with PTFE/silicon septum. An 18 G needle pierced the septum for the duration of the reaction to allow for constant air exposure. The suspension was stirred under irradiation with blue LEDs for 18 hours. The reaction was quenched with saturated sodium bicarbonate solution (10 mL) and extracted with ethyl acetate (5 x 5 mL). The extracts were combined and passed through a plug of silica which was flushed with acetone, and the solution was transferred to a 20-mL scintillation vial. An internal standard of dodecane (10 μL, 0.044 mmol) was delivered to the vial, and the contents were thoroughly mixed. A sample was analyzed by gas chromatography, and the integral values were used to calculate conversion, alkylpyridine (dimethyl 2-(1-(pyridin-2-yl)ethyl)malonate) yield, and hydrodehalogenation product (pyridine) yield.

III-E. Gas Chromatography Method Conditions:

The gas chromatography system hardware are reported in section I-B, General Analytical Information. The injection volume for each trial is 0.5 μL. The initial oven temperature was set to 50 °C, and the ramp rate was programmed to 20 °C/min until reaching 150 °C. With no hold time, the temperature ramp rate is adjusted to 25 °C/min until reaching the maximum temperature of 325 °C. Maximum temperature is held for one minute before concluding the run.

III-F. Optimization Table:

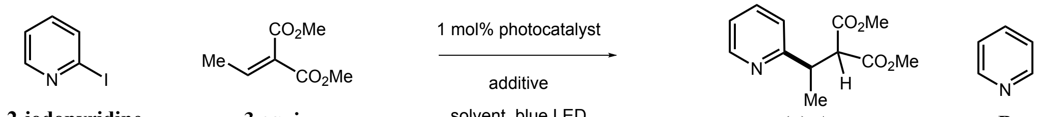

2-iodopyridine **3 equiv** **(±)-A** **B**

entry	photocatalyst	solvent	additive	% yield **A**	% yield **B**	selectivity (**A**:**B**)
1	$Ir(dF(CF_3)ppy)_2dtbpy{\bullet}PF_6$	MeCN	NEt_3 (3 eq)	24	5	5:1
2	$Ir(dF(CF_3)ppy)_2dtbpy{\bullet}PF_6$	MeCN	NBu_3 (3 eq)	29	6	5:1
3	$Ir(dF(CF_3)ppy)_2dtbpy{\bullet}PF_6$	MeCN	ipr_2NEt (3 eq)	12	3	4:1
4	$Ir(dF(CF_3)ppy)_2dtbpy{\bullet}PF_6$	MeCN	NBu_3 (3 eq), HCO_2H (3 eq)	27	5	5:1
5	$Ir(dF(CF_3)ppy)_2dtbpy{\bullet}PF_6$	MeCN	ipr_2NEt (3 eq), HEH (1.5 eq)	43	20	2:1
6	$Ru(bpy)_3Cl_2$	MeCN	HEH (1.3 eq)	24	6	4:1
7	$Ir(ppy)_3$	MeCN	HEH (1.3 eq)	53	13	4:1
8	$Ir(dF(CF_3)ppy)_2dtbpy{\bullet}PF_6$	MeCN	HEH (1.3 eq)	50	11	9:2
9	$Ir(ppy)_2dtbpy{\bullet}PF_6$	MeCN	HEH (1.3 eq)	62	15	4:1
10	$Ru(bpy)_3Cl_2$	DMSO	HEH (1.3 eq)	50	15	7:2
11	$Ir(ppy)_3$	DMSO	HEH (1.3 eq)	68	25	7:2
12	$Ir(dF(CF_3)ppy)_2dtbpy{\bullet}PF_6$	DMSO	HEH (1.3 eq)	50	15	7:2
13	$Ir(ppy)_2dtbpy{\bullet}PF_6$	DMSO	HEH (1.3 eq)	59	25	5:2
14	$Ir(dF(CF_3)ppy)_2dtbpy{\bullet}PF_6$	DMF/H_2O (3:1)	HEH (1.3 eq)	75	15	5:1
15	$Ir(dF(CF_3)ppy)_2dtbpy{\bullet}PF_6$	$MeOH/H_2O$ (3:1)	HEH (1.3 eq)	78	10	8:1
16	$Ir(dF(CF_3)ppy)_2dtbpy{\bullet}PF_6$	$MeCN/H_2O$ (3:1)	HEH (1.3 eq)	81	14	6:1
17[a]	$Ir(dF(CF_3)ppy)_2dtbpy{\bullet}PF_6$	$DMSO/H_2O$ (3:1)	HEH (1.3 eq)	72	8	9:1
18[b]	$Ir(dF(CF_3)ppy)_2dtbpy{\bullet}PF_6$	$DMSO/H_2O$ (3:1)	HEH (1.3 eq)	89	6	15:1
19[c]	$Ir(dF(CF_3)ppy)_2dtbpy{\bullet}PF_6$	$DMSO/H_2O$ (3:1)	HEH (1.3 eq)	0	0	-
20	none	$DMSO/H_2O$ (3:1)	HEH (1.3 eq)	20	2	10:1
21	$Ir(dF(CF_3)ppy)_2dtbpy{\bullet}PF_6$	$DMSO/H_2O$ (3:1)	HEH (1.3 eq)	96	4	48:2
22[d]	$Ir(dF(CF_3)ppy)_2dtbpy{\bullet}PF_6$	$DMSO/H_2O$ (3:1)	HEH (1.3 eq)	92	4	45:2

[a]1.5 equiv dimethyl ethylidenemalonate. [b]2.0 equiv dimethyl ethylidenemalonate. [c]No light. [d]Exposed to open atmosphere.

IV. Preparation of Starting Materials:

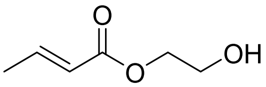

2-hydroxyethyl (E)-but-2-enoate: To a solution of pyridine (1.56 g, 15 mmol, 1.5 equiv) in ethylene glycol (50 mL) and THF (50 mL) at 0 °C was added but-2-enoyl chloride by syringe over 10 minutes. The mixture was allowed to warm to room temperature and continued stirring for 5.5 hours. The THF was removed by rotary evaporation, and the residual solution was partitioned between ethyl acetate (150 mL) and 1M HCl solution

(100 mL). The layers were separated, and the organic phase was washed with 1M HCl solution (2 x 100 mL), saturated sodium bicarbonate solution (100 mL), and brine (100 mL). The organic layer was dried over sodium sulfate, filtered and concentrated by rotary evaporation to afford the title compound (1.10 g, 54% yield) as a clear, colorless oil.

^{1}H NMR (300 MHz, $CDCl_3$) δ 6.93 – 6.66 (m, 1H), 5.68 (d, *J* = 15.7 Hz, 1H), 4.02 (t, *J* = 4.8 Hz, 2H), 3.70 (s, 1H), 3.61 (d, *J* = 4.8 Hz, 2H), 1.68 (d, *J* = 6.9 Hz, 3H).

^{13}C NMR (75 MHz, $CDCl_3$) δ 166.7, 145.3, 122.1, 65.6, 60.4, 17.8.

FTIR (neat) ν_{max}: 3429, 2948, 2917, 2881, 2359, 1716, 1655, 1444, 1375, 1311, 1292, 1263, 1178, 1103, 1080, 1033, and 967 cm^{-1}.

HRMS (NSI) *m/z*: $[M+Na]^+$ calcd. for $C_6H_{10}O_3Na$, 153.0522; found, 153.0522.

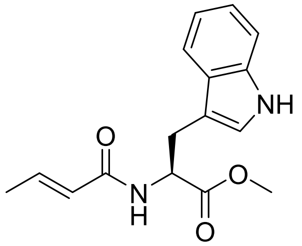

Methyl (E)-but-2-enoyl-L-tryptophanate: To a solution of methyl L-tryptophanate (2.4 g, 9.4 mmol, 1 equiv) and pyridine (1.87 g, 23.6 mmol, 2.5 equiv) in dichloromethane (80 mL) at 0 °C was added but-2-enoyl chloride (1.01 g, 10.34 mmol, 1.1 equiv) by syringe over 10 minutes. The mixture was allowed to warm to room temperature and continued stirring for 3.5 hours. The solvent was removed by rotary evaporation, and the residue was dissolved in ethyl acetate (150 mL). The solution was washed with 1M HCl solution (3 x 100 mL), saturated sodium bicarbonate solution (100 mL), and brine (100 mL). The organic layer was dried over sodium sulfate, filtered, and concentrated by rotary evaporation to afford the title compound (2.22 g, 82% yield) as a yellow solid.

Mp: 118 – 120 °C

^{1}H NMR (300 MHz, $CDCl_3$) δ 8.26 (s, 1H), 7.52 (d, *J* = 7.6 Hz, 1H), 7.35 (d, *J* = 7.8 Hz, 1H), 7.19 (t, *J* = 7.3, 6.7 Hz, 1H), 7.11 (td, *J* = 7.5, 2.9 Hz, 1H), 6.97 (d, *J* = 2.5 Hz, 1H), 6.85 (dq, *J* = 15.4, 6.5, 6.5 Hz, 1H), 6.01 (d, *J* = 7.9 Hz, 1H), 5.81 – 5.70 (m, 1H), 5.03 (dt, J = 7.9, 5.2 Hz, 1H), 3.69 (d, *J* = 0.6 Hz, 3H), 3.35 (d, *J* = 5.3 Hz, 2H), 1.82 (dd, *J* = 6.9, 1.7 Hz, 3H).

^{13}C NMR (75 MHz, $CDCl_3$) δ 172.5, 165.5, 140.9, 136.1, 127.6, 124.6, 122.9, 122.2, 119.6, 118.6, 111.3, 109.9, 53.0, 52.4, 27.7, 17.8.

FTIR (neat) ν_{max}: 3410, 3310, 2358, 2339, 1736, 1669, 1625, 1537, 1458, 1438, 1430, 1210, 1174, 967, and 738 cm^{-1}.

HRMS (NSI) *m/z*: $[M+H]^+$ calcd. for $C_{16}H_{19}N_2O_3$, 287.1390; found, 287.1386.

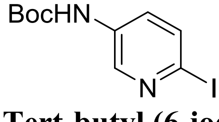

Tert-butyl (6-iodopyridin-3-yl)carbamate:

To a solution of 6-iodopyridin-3-amine (616.1 mg, 2.80 mmol, 1 equiv) in tetrahydrofuran (10 mL) at 0 °C was added a solution of sodium bis(trimethylsilyl)amide in tetrahydrofuran (1.0 M, 5.60 mL, 2 equiv). After stirring the reaction at 0 °C for 30 mins and then room temperature 15 mins, di-tert-butyl dicarbonate (642.1 mg, 2.94 mmol, 1.05 equiv) was

added slowly. The resulting mixture was stirred overnight at room temperature. The reaction was diluted with ethyl acetate and washed with saturated sodium bicarbonate solution, water and brine. After drying over magnesium sulfate, the solid was filtered off and the filtrated was concentrated by rotary evaporation. The crude reaction mixture was purified by flash chromatography (hexane:ethyl acetate = 4:1) to afford the product (841.8 mg, 94% yield) as a white solid.

Mp: 136 – 138 °C

^{1}H NMR (400 MHz, $CDCl_3$) δ 8.21 (dd, J = 2.8, 0.7 Hz, 1H), 7.68 (s, 1H), 7.59 (dd, J = 8.5, 0.6 Hz, 1H), 6.58 (s, 1H), 1.49 (s, 9H).

^{13}C NMR (125 MHz, $CDCl_3$) δ 152.7, 141.2, 135.9, 134.5, 127.9, 108.3, 81.4, 28.3.

FTIR (neat) ν_{max}: 3212, 3149, 2977, 1717, 1589, 1519, 1453, 1362, 1249, and 1150 cm^{-1}.

HRMS (NSI) *m/z*: $[M+H]^+$ calcd. for $C_{10}H_{14}N_2O_2I$, 263.9516; found, 263.9515.

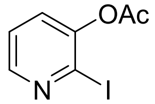

2-iodopyridin-3-yl acetate:

To a solution of 2-iodopyridin-3-ol (1.00 g, 4.54 mmol, 1 equiv) in dichloromethane (10 mL) at 0 °C was added triethylamine (1.30 mL, 9.33 mmol, 2 equiv) and acetyl chloride (0.50 mL, 7.03 mmol, 1.5 equiv) subsequently. The reaction was warmed slowly to room temperature in the ice-water bath and stirred overnight. It was then quenched with saturated sodium bicarbonate solution and extracted with dichloromethane three times. The combined organic layers were washed with saturated sodium bicarbonate solution, water and brine. After drying over magnesium sulfate, the solid was filtered off and the filtrate was concentrated by rotary evaporation. The crude reaction mixture was purified by flash column chromatography (hexane:ethyl acetate = 4:1) to afford the product (1.10 g, 92% yield) as a white solid.

Mp: 44 – 46 °C

^{1}H NMR (400 MHz, $CDCl_3$) δ 8.25 (dd, J = 4.6, 1.7 Hz, 1H), 7.35 (dd, J = 8.0, 1.7 Hz, 1H), 7.30 – 7.06 (m, 1H), 2.38 (s, 3H).

^{13}C NMR (125 MHz, $CDCl_3$) δ 168.1, 148.6, 147.9, 130.1, 123.6, 115.5, 21.3.

FTIR (neat) ν_{max}: 3052, 1766, 1563, 1441, 1400, 1367, and 1173 cm^{-1}.

HRMS (NSI) *m/z*: $[M+H]^+$ calcd. for $C_7H_7NO_2I$, 263.9516; found, 263.9515.

V. Procedure and Characterization Data

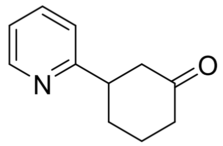

3-(pyridine-2-yl)cyclohexan-1-one (Table 2, entry 1): following the general procedure, the reaction of 2-iodopyridine (205 mg, 1.00 mmol, 1 equiv), cyclohex-2-en-1-one (0.290 mL, 3.00 mmol, 3 equiv), [Ir{dF(CF_3)ppy}$_2$(dtbbpy)]PF_6 (12.0 mg, 0.011 mmol, 0.011 equiv) and Hantzsch ester (329 mg, 1.3 mmol, 1.3 equiv) provided the product (148 mg,

85% yield) as a pale yellow oil after purification by flash column chromatography (hexanes:ethyl acetate = 3:1 then 1:1).
^{1}H NMR (300 MHz, $CDCl_3$) δ 8.41 (d, J = 4.6 Hz, 1H), 7.49 (td, J = 7.7, 1.9 Hz, 1H), 7.00 (dd, J = 7.6, 5.0 Hz, 2H), 3.17 – 2.90 (m, 1H), 2.68 (dd, J = 14.2, 11.9 Hz, 1H), 2.52 – 2.37 (m, 1H), 2.35 – 2.20 (m, 2H), 2.06 – 1.90 (m, 2H), 1.90 – 1.49 (m, 2H).
^{13}C NMR (75 MHz, $CDCl_3$) δ 211.1, 162.6, 149.2, 136.6, 121.7, 121.6, 46.7, 46.2, 41.1, 31.5, 25.1.
FTIR (neat) ν_{max}: 3007, 2937, 2862, 1706, 1588, 1434, 1221, 774, and 748 cm^{-1}.
HRMS (NSI) *m/z*: $[M+H]^+$ calcd. for $C_{11}H_{14}NO$, 176.1070; found, 176.1069.

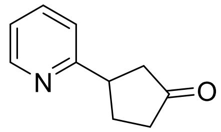

3-(pyridine-2-yl)cyclopentan-1-one (Table 2, entry 2): following the general procedure, the reaction of 2-iodopyridine (205 mg, 1.00 mmol, 1 equiv), cyclopent-2-en-1-one (0.251 mL, 3.00 mmol, 3 equiv), $[Ir\{dF(CF_3)ppy\}_2(dtbbpy)]PF_6$ (11.0 mg, 0.010 mmol, 0.01 equiv) and Hantzsch ester (329 mg, 1.3 mmol, 1.3 equiv) provided the product (134 mg, 84% yield) as a pale yellow oil after purification by flash column chromatography (hexanes:ethyl acetate = 3:1 then 1:1).
^{1}H NMR (300 MHz, $CDCl_3$) δ 8.44 (dd, J = 4.9, 0.9 Hz, 1H), 7.53 (td, J = 7.7, 1.9 Hz, 1H), 7.11 (d, J = 7.8 Hz, 1H), 7.08 – 6.97 (m, 1H), 3.46 (tdd, J = 9.5, 7.9, 6.1 Hz, 1H), 2.68 – 2.45 (m, 2H), 2.44 – 1.94 (m, 4H).
^{13}C NMR (75 MHz, $CDCl_3$) δ 218.6, 162.1, 149.4, 136.5, 122.0, 121.7, 44.4, 43.9, 38.3, 30.2.
FTIR (neat) ν_{max}: 3008, 2961, 2900, 1735, 1590, 1473, 1436, 1150, 1134, 785, and 749 cm^{-1}.
HRMS (NSI) *m/z*: $[M+H]^+$ calcd. for $C_{10}H_{12}NO$, 162.0913; found, 162.0912.

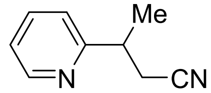

3-pyridin-2-yl(butanenitrile (Table 2, entry 3): following the general procedure, the reaction of 2-iodopyridine (205 mg, 1.00 mmol, 1 equiv), crotononitrile (0.245 mL, 3.00 mmol, 3 equiv), $[Ir\{dF(CF_3)ppy\}_2(dtbbpy)]PF_6$ (11.0 mg, 0.010 mmol, 0.010 equiv) and Hantzsch ester (329 mg, 1.30 mmol, 1.3 equiv) provided the product (101 mg, 72% yield) as a pale yellow oil after purification by flash column chromatography (hexanes:ethyl acetate = 5:1).
^{1}H NMR (300 MHz, $CDCl_3$) δ 8.52 (d, J = 4.9 Hz, 1H), 7.62 (td, J = 7.7, 1.8 Hz, 1H), 7.24 – 7.06 (m, 2H), 3.34 – 3.18 (m, 1H), 2.80 (dd, J = 16.7, 6.7 Hz, 1H), 2.69 (dd, J = 16.6, 7.4 Hz, 1H), 1.41 (d, J = 6.9 Hz, 3H).
^{13}C NMR (75 MHz, $CDCl_3$) δ 161.6, 149.5, 136.8, 122.2, 121.8, 119.0, 38.3, 23.9, 20.1.
FTIR (neat) ν_{max}: 3053, 3010, 2971, 2931, 2875, 2246, 1590, 1570, 1474, 1435, 991, 785, and 749 cm^{-1}.
HRMS (NSI) *m/z*: $[M+H]^+$ calcd. for $C_9H_{11}N_2$, 147.0917; found, 147.0916.

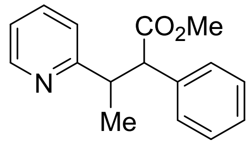

Methyl 2-phenyl-3-(pyridin-2-yl)butanoate (Table 2, entry 4): following the general procedure, the reaction of 2-iodopyridine (125 mg, 0.61 mmol, 1 equiv), methyl 2-phenylbut-2-enoate (320 mg, 1.81 mmol, 3 equiv), [Ir{dF(CF_3)ppy}$_2$(dtbbpy)]PF_6 (8.0 mg, 0.007 mmol, 0.012 equiv) and Hantzsch ester (179 mg, 0.71 mmol, 1.2 equiv) provided the product in an inseparable 4:3 mixture of diastereomers (128 mg, 84% yield) as colorless oil after purification by flash column chromatography (hexane:ethyl acetate = 20:1 then 10:1).

^{1}H NMR Major Diastereomers (300 MHz, Benzene-d6) δ 8.34 (dt, J = 4.7, 1.4 Hz, 1H), 7.18 – 6.15 (m, ArH), 3.69 (ddd, J = 11.1, 6.8, 2.3 Hz, 2H), 3.24 (s, 3H), 1.54 (d, J = 6.7 Hz, 3H).

^{1}H NMR Minor Diastereomers, characteristic signals (300 MHz, Benzene-d6) δ 8.45 (dt, J = 5.0, 1.3 Hz, 1H), 7.60 – 7.39 (m, 1H), 4.49 (d, J = 11.2 Hz, 1H), 3.00 (s, 3H), 1.03 (d, J = 7.0 Hz, 3H).

For the mixture of diastereomers:

^{13}C NMR (75 MHz, $CDCl_3$) δ 174.1, 173.8, 164.2, 162.6, 149.2, 149.1, 137.7, 137.6, 136.4, 135.9, 128.7, 128.6, 128.3, 128.1, 127.5, 126.9, 123.2, 123.0, 121.4, 121.2, 57.4, 56.7, 52.0, 51.7, 45.7, 44.7, 19.8, 19.2.

FTIR (neat) ν_{max}: 3063, 3029, 3006, 2968, 2950, 2873, 2842, 1731, 1589, 1454, 1158, 733, and 698 cm^{-1}.

HRMS (NSI) *m/z*: $[M+H]^+$ calcd. for $C_{16}H_{18}NO_2$, 256.1334; found, 256.1332.

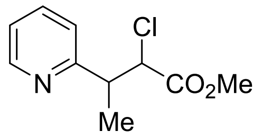

Methyl 2-chloro-3-(pyridine-2-yl)butanoate (Table 2, entry 5): following the general procedure, the reaction of 2-iodopyridine (205 mg, 1.00 mmol, 1 equiv), methyl 2-chlorobut-2-enoate (420 mg, 3.12 mmol, 3 equiv), [Ir{dF(CF_3)ppy}$_2$(dtbbpy)]PF_6 (11.0 mg, 0.010 mmol, 0.01 equiv) and Hantzsch ester (331 mg, 1.31 mmol, 1.3 equiv) provided the product in an inseparable 4:1 mixture of diastereomers (185 mg, 87% yield) as a colorless oil after purification by flash column chromatography (hexane:ethyl acetate = 10:1 then 5:1).

^{1}H NMR Major Diastereomers (300 MHz, $CDCl_3$) δ 8.54 – 8.46 (m, 1H), 7.62 – 7.49 (m, 1H), 7.17 – 7.02 (m, 2H), 4.66 (d, J = 9.5 Hz, 1H), 3.74 (s, 3H), 3.46-3.35 (m, 1H), 1.28 (d, J = 7.0 Hz, 3H).

^{1}H NMR Minor Diastereomers, characteristic signals (300 MHz, $CDCl_3$) δ 8.44 (ddd, J = 4.9, 1.9, 0.9 Hz, 1H), 4.77 (d, J = 8.2 Hz, 1H), 3.56 (s, 3H), 1.40 (d, J = 7.0 Hz, 3H).

For the mixture of diastereomers:

^{13}C NMR (75 MHz, $CDCl_3$) δ 169.8, 169.7, 161.1, 160.5, 149.4, 149.1, 136.6, 136.4, 123.4, 122.5, 122.1, 122.0, 61.6, 60.4, 52.8, 52.6, 46.0, 45.0, 18.0, 16.7.

FTIR (neat) ν_{max}: 2976, 2953, 2877, 1744 1593, 1570, 1435, 1273, 1194, 1162, 991, 786, and 748 cm^{-1}.

HRMS (NSI) *m/z*: $[M+H]^+$ calcd. for $C_{10}H_{13}NO_2Cl$, 214.0629; found, 214.0629.

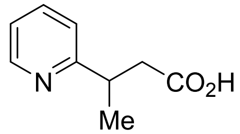

3-(pyridin-2-yl)butanoic acid (Table 2, entry 6):
A 30-mL screw-top test tube equipped with a stir bar was charged with Hantzsch ester (83.0 mg, 0.33 mmol, 1.3 equiv), [Ir{dF(CF_3)ppy}$_2$(dtbbpy)]PF_6 (2.8 mg, 0.010 mmol, 0.010 equiv), but-2-enoic acid (64.5 mg, 0.75 mmol, 3 equiv), and 2-iodopyridine (53 mg, 0.25 mmol, 1 equiv). The tube was sealed with PTFE/silicon septum and connected to a vacuum line. The atmosphere was exchanged by applying vacuum and backfilling with N_2 (this process was conducted a total of three times). Under N_2 atmosphere, the tube was charged with degassed solvent (3:1 DMSO:H_2O, 0.1 M) by syringe. The resulting suspension was stirred under irradiation with blue LEDs for 18 hours. The water was removed from the crude mixture by rotary evaporation. An internal standard of 1,3,5-trimethoxybenzene (43.8 mg, 0.26 mmol, 1.04 equiv) was added. Crude NMR of the mixture was taken (d1=10 s), and integration of the aromatic protons in the resultant ^{1}H spectrum indicated 74 % yield of the title compound.
^{1}H NMR, characteristic signals: (300 MHz, DMSO-d6) δ 8.81 (td, *J* = 5.9, 1.0 Hz, 1H), 8.06 (d, *J* = 8.0 Hz, 1H), 7.89 (ddd, *J* = 7.6, 5.8, 1.2 Hz, 1H), 3.59 – 3.43 (m, 1H), 2.90 (dd, *J* = 17.1, 8.6 Hz, 1H), 2.77 (dd, J = 17.1, 6.3 Hz, *1*H).

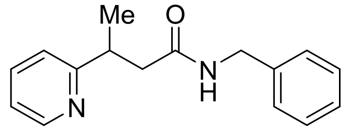

N-benzyl-3-(pyridine-2yl)butanamide (Table 2, entry 7): following the general procedure, the reaction of 2-iodopyridine (205 mg, 1.00 mmol, 1 equiv), N-benzylbut-2-enamide (526 mg, 3.01mmol, 3 equiv), [Ir{dF(CF_3)ppy}$_2$(dtbbpy)]PF_6 (11.0 mg, 0.010 mmol, 0.010 equiv) and Hantzsch ester (331 mg, 1.31 mmol, 1.3 equiv) provided the product (171 mg, 70% yield) as a pale yellow oil after purification by flash column chromatography (hexanes:ethyl acetate = 1:1 then 1:2).
Mp: 70 – 72 °C
^{1}H NMR (300 MHz, $CDCl_3$) δ 8.43 – 8.33 (m, 1H), 7.53 (tdd, *J* = 7.6, 1.9, 0.9 Hz, 1H), 7.24 – 7.10 (m, 4H), 7.09 – 7.01 (m, 1H), 7.00 – 6.94 (m, 2H), 6.70 (s, 1H), 4.33 (dd, *J* = 14.9, 6.1 Hz, 1H), 4.19 (dd, *J* = 14.9, 5.4 Hz, 1H), 3.49 – 3.33 (m, 1H), 2.73 (dd, *J* = 13.9, 8.8 Hz, 1H), 2.49 (dd, *J* = 14.2, 6.0 Hz, 1H), 1.26 (d, *J* = 6.9 Hz, 3H).
^{13}C NMR (75 MHz, Chloroform-d) δ 171.87, 164.46, 148.90, 138.38, 136.66, 128.42, 127.38, 127.08, 122.71, 121.51, 43.19, 43.08, 38.64, 21.15.
FTIR (neat) ν_{max}: 3206, 3045, 2974, 2962, 2909, 1667, 1567, 1551, 1474, 1291, 1241, 792, 753, 736, and 705 cm^{-1}.
HRMS (NSI) *m/z*: [M+H]$^+$ calcd. for $C_{16}H_{19}N_2O$, 255.1492; found, 255.1491.

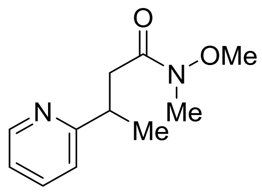

N-methoxy-N-methyl-3-(pyridin-2-yl)butanamide (Table 2, entry 8): following the general procedure, the reaction of 2-iodopyridine (205 mg, 1.00 mmol, 1 equiv), N-methoxy-N-methylbut-2-enamide (387 mg, 3.00 mmol, 3 equiv), [Ir{dF(CF_3)ppy}$_2$(dtbbpy)]PF_6 (11.4 mg, 0.010 mmol, 0.010 equiv) and Hantzsch ester (334 mg, 1.32 mmol, 1.3 equiv) provided the product (121 mg, 63% yield) as a pale yellow oil after purification by flash column chromatography (hexane:ethyl acetate = 1:1 then ethyl acetate).

^{1}H NMR (300 MHz, $CDCl_3$) δ 8.44 (dt, J = 4.9, 0.9 Hz, 1H), 7.50 (td, J = 7.6, 1.9 Hz, 1H), 7.13 (d, J = 7.8 Hz, 1H), 7.01 (ddt, J = 7.5, 4.8, 1.0 Hz, 1H), 3.56 (s, 3H), 3.41 (dt, J = 14.1, 7.0 Hz, 1H), 3.04 (s, 3H), 2.97 (dd, J = 16.0, 7.6 Hz, 1H), 2.62 (dd, J = 16.1, 6.8 Hz, 1H), 1.25 (d, J = 7.0 Hz, 3H).

^{13}C NMR (75 MHz, $CDCl_3$) δ 165.0, 149.0, 136.4, 122.4, 121.2, 61.1, 38.2, 37.4, 21.0 (2xC).

FTIR (neat) ν_{max}: 3349, 2965, 2936, 2873, 1655, 1590, 1568, 1473, 1433, 1415, 1348, 1176, 1149, 1120, 997, 784, and 749 cm^{-1}.

HRMS (NSI) *m/z*: [M+H]$^+$ calcd. for $C_{11}H_{17}N_2O_2$, 209.1285; found, 209.1283.

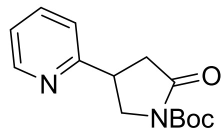

Tert-butyl 2-oxo-4-(pyridine-2-yl)pyrrolidine-1-carboxylate (Table 2, entry 9): following the general procedure, the reaction of 2-iodopyridine (205.0 mg, 1.00 mmol, 1 equiv), tert-butyl 2-oxo-4-pyridin-2-yl)pyrrolidine-1-carboxylate (560 mg, 3.06 mmol, 3 equiv), [Ir{dF(CF_3)ppy}$_2$(dtbbpy)]PF_6 (11.0 mg, 0.010 mmol, 0.01 equiv) and Hantzsch ester (329.0 mg, 1.3 mmol, 1.3 equiv) provided the product (159 mg, 61% yield) as a colorless oil after purification by flash column chromatography (hexane:ethyl acetate = 5:1 then 1:1).

^{1}H NMR (300 MHz, $CDCl_3$) δ 8.53 – 8.48 (m, 1H), 7.65 – 7.53 (m, 1H), 7.13 (ddd, J = 7.6, 4.2, 1.0 Hz, 2H), 4.07 (dtd, J = 10.7, 8.6, 0.9 Hz, 1H), 3.84 (dt, J = 10.7, 8.6 Hz, 1H), 3.68 – 3.52 (m, 1H), 2.93 (dt, J = 17.4, 9.7 Hz, 1H), 2.76 (dt, J = 17.2, 8.7 Hz, 1H), 1.44 (s, 9H).

^{13}C NMR (75 MHz, $CDCl_3$) δ 173.2, 159.4, 149.8, 136.8, 122.4, 122.3, 82.8, 51.8, 39.1, 38.0, 28.0.

FTIR (neat) ν_{max}: 2978, 2931, 1779, 1746, 1711, 1367, 1350, 1300, 1285, 1255, 1147, 777, and 748 cm^{-1}.

HRMS (NSI) *m/z*: [M+H]$^+$ calcd. for $C_{14}H_{19}N_2O_3$, 263.1390; found, 263.1389.

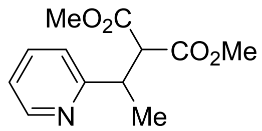

Dimethyl 2-(1-pyridin-2-yl)ethyl)malonate (Table 2, entry 10): following the general procedure, the reaction of 2-iodopyridine (205 mg, 1.00 mmol, 1 equiv), dimethyl 2-ethylidinemalonate (478 mg, 3.02 mmol, 3 equiv), [Ir{dF(CF_3)ppy}$_2$(dtbbpy)]PF_6 (11.1 mg, 0.010 mmol, 0.01 equiv) and Hantzsch ester (330 mg, 1.30 mmol, 1.3 equiv) provided the product (211 mg, 89% yield) as a pale yellow oil after purification by flash column chromatography (hexanes:diethyl ether = 5:3).

^{1}H NMR (300 MHz, $CDCl_3$) δ 8.38 (ddd, J = 4.9, 1.9, 0.9 Hz, 1H), 7.50 (td, J = 7.6, 1.9 Hz, 1H), 7.12 (d, J = 7.8 Hz, 1H), 7.00 (ddd, J = 7.5, 4.9, 1.2 Hz, 1H), 4.01 (d, J = 10.4 Hz, 1H), 3.66 (s, 3H), 3.60 – 3.51 (m, 1H), 3.42 (s, 3H), 1.20 (d, J = 7.0 Hz, 3H).

^{13}C NMR (75 MHz, $CDCl_3$) δ 169.2, 168.7, 149.0, 136.4, 122.8, 121.6, 56.5, 52.4, 52.1, 41.1, 19.1.

FTIR (neat) ν_{max}: 2954, 2846, 1751, 1731, 1434, 1287, 1271, 1252, 1218, 1192, 1146, 787, and 749 cm^{-1}.

HRMS (NSI) *m/z*: $[M+H]^+$ calcd. for $C_{12}H_{16}NO_4$, 238.1074; found, 238.1073.

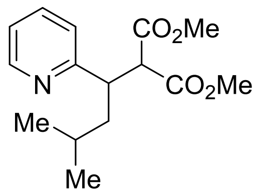

Dimethyl 2-(2-methyl-1-(pyridin-2-yl)propyl)malonate (Table 2, entry 11): following the general procedure, the reaction of 2-iodopyridine (205 mg, 1.00 mmol, 1 equiv), dimethyl 2-(3-methylbutylidene)malonate (606 mg, 3.03 mmol, 3 equiv), [Ir{dF(CF_3)ppy}$_2$(dtbbpy)]PF_6 (11.0 mg, 0.010 mmol, 0.010 equiv) and Hantzsch ester (329 mg, 1.30 mmol, 1.3 equiv) provided the product (186 mg, 67% yield) as a pale yellow oil after purification by flash column chromatography (hexane:ethyl acetate = 10:1 then 5:1).

^{1}H NMR (300 MHz, $CDCl_3$) δ 8.49 (d, J = 4.8 Hz, 1H), 7.60 – 7.47 (m, 1H), 7.16 (d, J = 7.8 Hz, 1H), 7.10 – 7.01 (m, 1H), 3.96 (d, J = 10.5 Hz, 1H), 3.73 (s, 3H), 3.55 (td, J = 11.1, 3.2 Hz, 1H), 3.41 (s, 3H), 1.82 (td, J = 12.2, 3.2 Hz, 1H), 1.24 (ddd, J = 13.1, 10.1, 3.3 Hz, 1H), 1.17 -1.02 (m, 1H), 0.85 (d, J = 6.3 Hz, 3H), 0.72 (d, J = 6.5 Hz, 3H).

^{13}C NMR (75 MHz, $CDCl_3$) δ 169.1, 168.6, 160.8, 149.4, 135.9, 124.5, 121.6, 57.2, 52.5, 52.2, 44.8, 42.0, 25.1, 23.8, 21.0.

FTIR (neat) ν_{max}: 3007, 2954, 2869, 2947, 1753, 1734, 1570, 1434, 1258, 1243, 1145, 749, and 732 cm^{-1}.

HRMS (NSI) *m/z*: $[M+H]^+$ calcd. for $C_{15}H_{22}NO_4$, 280.1541; found, 280.1543.

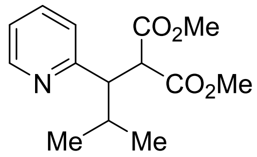

Dimethyl 2-(2-methyl-1-(pyridin-2-yl)propyl)malonate (Table 2, entry 12): following the general procedure, the reaction of 2-iodopyridine (164 mg, 0.80 mmol, 1 equiv), dimethyl 2-(2-methylpropylidene)malonate (446 mg, 2.40 mmol, 3 equiv), [Ir{dF(CF_3)ppy}$_2$(dtbbpy)]PF_6 (11.0 mg, 0.010 mmol, 0.013 equiv) and Hantzsch ester (263 mg, 1.04 mmol, 1.3 equiv) provided the product (105 mg, 50% yield) as a pale yellow oil after purification by flash column chromatography (hexane:ethyl acetate = 10:1 then 5:1).

^{1}H NMR (300 MHz, $CDCl_3$) δ 8.51 – 8.41 (m, 1H), 7.55 (td, J = 7.7, 1.9 Hz, 1H), 7.15 (d, J = 7.8 Hz, 1H), 7.07 (dd, J = 7.5, 5.0 Hz, 1H), 4.38 (d, J = 11.1 Hz, 1H), 3.75 (s, 3H), 3.52 (d, J = 4.4 Hz, 1H), 3.45 (s, 3H), 2.06 – 1.86 (m, 1H), 0.87 (d, J = 6.9 Hz, 3H), 0.75 (d, J = 6.8 Hz, 3H).

^{13}C NMR (75 MHz, $CDCl_3$) δ 169.7, 168.9, 159.1, 148.5, 135.5 125.3, 121.5, 54.1, 52.6, 52.2, 51.6, 30.2, 21.5, 17.4.

FTIR (neat) ν_{max}: 2957, 2933, 2876, 2847, 1754, 1732, 1434, 1264, 1168, 1144, 750, and

HRMS (NSI) *m/z*: $[M+H]^+$ calcd. for $C_{14}H_{20}NO_4$, 266.1383; found, 266.1387.

731 cm^{-1}.

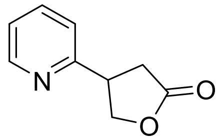

4-(pyridine-2-yl)dihydrofuran-2(3H)-one (Table 2, entry 13): following the general procedure, the reaction of 2-iodopyridine (205 mg, 1.00 mmol, 1 equiv), furan-2(5H)-one (0.252 mg, 3.00 mmol, 3 equiv), [Ir{dF(CF_3)ppy}$_2$(dtbbpy)]PF_6 (11.0 mg, 0.010 mmol, 0.01 equiv) and Hantzsch ester (329 mg, 1.3 mmol, 1.3 equiv) provided the product (116 mg, 71% yield) as a yellow oil after purification by flash column chromatography (hexanes:ethyl acetate = 1:1 then 1:2).

^{1}H NMR (400 MHz, $CDCl_3$) δ 8.49 (d, J = 4.8 Hz, 1H), 7.59 (td, J = 7.7, 1.9 Hz, 1H), 7.14 (t, J = 6.5 Hz, 2H), 4.58 (t, J = 8.4 Hz, 1H), 4.36 (t, J = 8.3 Hz, 1H), 3.85 (p, J = 8.3 Hz, 1H), 2.91 (dd, J = 17.4, 8.6 Hz, 1H), 2.78 (dd, J = 17.4, 8.8 Hz, 1H).

^{13}C NMR (100 MHz, $CDCl_3$) δ 176.6, 158.5, 149.8, 136.9, 122.6, 122.4, 72.8, 42.6, 34.3.

FTIR (neat) ν_{max}: 3054, 3009, 2911, 1770, 1592, 1160, 1019, 994, and 731 cm^{-1}.

HRMS (NSI) *m/z*: $[M+H]^+$ calcd. for $C_9H_{10}NO_2$, 164.0706; found, 164.0706.

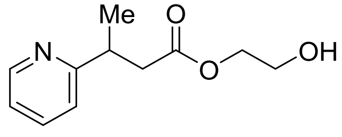

2-hydroxyethyl 3-(pyridin-2-yl)butanoate (Table 2, entry 14): following the general procedure, the reaction of 2-iodopyridine (205 mg, 1.00 mmol, 1 equiv), 2-hydroxyethylbut-2-enoate (395 mg, 3.04 mmol, 3 equiv), [Ir{dF(CF_3)ppy}$_2$(dtbbpy)]PF_6 (11.1 mg, 0.010 mmol, 0.010 equiv) and Hantzsch ester (333 mg, 1.32 mmol, 1.3 equiv)

provided the product (142 mg, 68% yield) as a pale yellow oil after purification by flash column chromatography (hexane:ethyl acetate = 1:1 then ethyl acetate).

^{1}H NMR (300 MHz, $CDCl_3$) δ 8.40 (dt, J = 4.9, 0.8 Hz, 1H), 7.56 (td, J = 7.7, 1.8 Hz, 1H), 7.15 (d, J = 7.9 Hz, 1H), 7.07 (td, J = 4.9, 4.9, 1.4 Hz, 1H), 4.15 (dt, J = 11.5, 4.6 Hz, 1H), 4.02 (dt, J = 11.5, 4.7 Hz, 1H), 3.66 (t, J = 4.7 Hz, 2H), 3.48 – 3.29 (m, 1H), 2.71 (dd, J = 15.3, 8.5 Hz, 1H), 2.58 (dd, J = 15.3, 6.3 Hz, 1H), 1.27 (d, J = 7.0 Hz, 3H).

^{13}C NMR (75 MHz, $CDCl_3$) δ 172.4, 164.0, 148.8, 136.9, 121.70, 121.65, 65.9, 60.3, 41.3, 38.1, 20.5.

FTIR (neat) ν_{max}: 3356, 2965, 2874, 1729, 1593, 1435, 1205, 1166, 786, and 750 cm^{-1}.

HRMS (NSI) *m/z*: $[M+H]^+$ calcd. for $C_{11}H_{16}NO_3$, 210.1125; found, 210.1124.

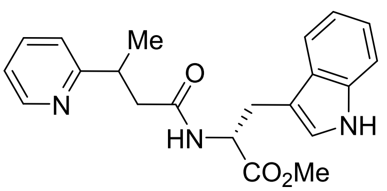

Methyl (3-(pyridin-2-yl)butanoyl)-D-tryptophanate (Table 2, entry 15): following the general procedure, the reaction of 2-iodopyridine (205 mg, 1.00 mmol, 1 equiv), methylbut-2-enoyl-D-tryptophanate (860 mg, 3.01 mmol, 3 equiv), [Ir{dF(CF_3)ppy}$_2$(dtbbpy)]PF_6 (11.0 mg, 0.010 mmol, 0.010 equiv) and Hantzsch ester (329 mg, 1.30 mmol, 1.3 equiv) provided the product in an inseparable 1:1 mixture of diastereomers (147 mg, 41% yield) as a yellow solid after purification by flash column chromatography (hexane:ethyl acetate = 1:1 then ethyl acetate).

For the mixture of diastereomers:

^{1}H NMR (300 MHz, Benzene-d_6) δ 8.81 (s, $1H_{dr1}+1H_{dr2}$), 8.33 – 8.21 (m, $1H_{dr1}+1H_{dr2}$), 7.64 – 7.51 (m, $1H_{dr1}+1H_{dr2}$), 7.35 – 7.21 (m, $1H_{dr1}+1H_{dr2}$), 7.20 – 7.03 (m, $2H_{dr1}+2H_{dr2}$), 7.03 – 6.89 (m, $1H_{dr1}+1H_{dr2}$), 6.83 – 6.68 (m, $3H_{dr1}+3H_{dr2}$), 6.58 – 6.43 (m, $1H_{dr1}+1H_{dr2}$), 5.21 – 4.88 (m, $1H_{dr1}+1H_{dr2}$), 3.54 – 3.35 (m, $1H_{dr1}+1H_{dr2}$), 3.36 – 3.03 (m, $5H_{dr1}+5H_{dr2}$), 2.73 – 2.56 (m, $1H_{dr1}+1H_{dr2}$), 2.45 – 2.19 (m, $1H_{dr1}+1H_{dr2}$), 1.20 (d, J = 7.1 Hz, 3H), 1.20 (d, J = 6.9 Hz, 3H).

^{13}C NMR (75 MHz, Benzene-d_6) δ 172.5, 172.4, 171.9, 164.7, 164.6, 148.73, 148.70, 136.7, 136.1, 127.9, 127.9, 123.3, 122.2, 122.1, 121.8, 121.2, 121.1, 119.3, 118.6, 111.6, 109.8, 109.7, 53.3, 53.2, 51.5, 42.4, 38.4, 27.7, 27.7, 20.8, 20.7.

FTIR (neat) ν_{max}: 3284, 3055, 3009, 2953, 2957, 2871, 1736, 1648, 1592, 1518, 1434, 1354, 1340, 1211, and 740 cm^{-1}.

HRMS (NSI) *m/z*: $[M+H]^+$ calcd. for $C_{21}H_{24}N_3O_3$, 366.1812; found, 366.1812.

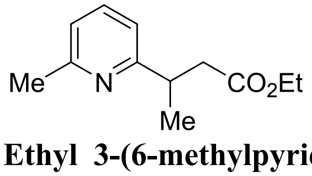

Ethyl 3-(6-methylpyridin-2-yl)butanoate (Table 3, entry 1): following the general procedure (A), the reaction of 2-bromo-6-methylpyridine (169.5 mg, 0.99 mmol, 1 equiv), ethyl crotonate (0.62 mL, 4.99 mmol, 5 equiv), [Ir{dF(CF_3)ppy}$_2$(dtbbpy)]PF_6 (6.0 mg, 0.0053 mmol, 0.005 equiv) and Hantzsch ester (324.6 mg, 1.28 mmol, 1.3 equiv) provided the product (155.3 mg, 76% yield) as a pale yellow oil after purification by flash column chromatography (dichloromethane:diethyl ether = 50:1 then 10:1).

^{1}**H NMR** (600 MHz, $CDCl_3$) δ 7.45 (t, J = 7.7 Hz, 1H), 6.94 (t, J = 7.6 Hz, 2H), 4.06 (q, J = 7.1 Hz, 2H), 3.41 – 3.30 (m, 1H), 2.82 (dd, J = 15.5, 7.2 Hz, 1H), 2.54 (dd, J = 15.5, 7.6 Hz, 1H), 2.49 (s, 3H), 1.29 (d, J = 7.0 Hz, 3H), 1.17 (t, J = 7.1 Hz, 3H).
^{13}**C NMR** (125 MHz, $CDCl_3$) δ 172.4, 163.5, 157.4, 136.3, 120.6, 118.0, 60.0, 40.8, 37.9, 24.3, 20.5, 14.0.
FTIR (neat): 2976, 1731, 1591, 1576, 1463, 1370, 1347, 1284, 1200, 1162 cm^{-1}.
HRMS (NSI) *m/z*: $[M+H]^+$ calcd. for $C_{12}H_{18}NO_2$, 208.1332; found, 208.1331.

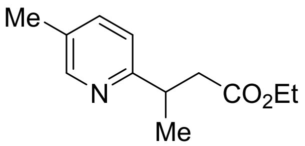

Ethyl 3-(5-methylpyridin-2-yl)butanoate (Table 3, entry 2): following the general procedure (A), the reaction of 2-bromo-5-methylpyridine (172.3 mg, 1.00 mmol, 1 equiv), ethyl crotonate (0.62 mL, 4.99 mmol, 5 equiv), [Ir{dF(CF_3)ppy}$_2$(dtbbpy)]PF_6 (5.7 mg, 0.0051 mmol, 0.005 equiv) and Hantzsch ester (310.3 mg, 1.26 mmol, 1.2 equiv) provided the product (127.9 mg, 62% yield) as a pale yellow oil after purification by flash column chromatography (hexane:diethyl ether = 4:1 then 2:1).
^{1}**H NMR** (600 MHz, $CDCl_3$) δ 8.33 (s, 1H), 7.38 (dd, J = 8.2, 1.9 Hz, 1H), 7.05 (d, J = 7.9 Hz, 1H), 4.08 – 4.01 (m, 2H), 3.39 – 3.31 (m, 1H), 2.82 (dd, J = 15.6, 7.6 Hz, 1H), 2.55 (dd, J = 15.6, 7.2 Hz, 1H), 2.27 (s, 3H), 1.29 (d, J = 7.0 Hz, 3H), 1.16 (t, J = 7.1 Hz, 3H).
^{13}**C NMR** (125 MHz, $CDCl_3$) δ 172.4, 161.2, 149.3, 136.8, 130.4, 121.1, 59.9, 40.8, 37.4, 20.6, 17.8, 13.9.
FTIR (neat): 2974, 1731, 1602, 1570, 1488, 1369, 1160 cm^{-1}.
HRMS (NSI) *m/z*: $[M+H]^+$ calcd. for $C_{12}H_{18}NO_2$, 208.1332; found, 208.1332.

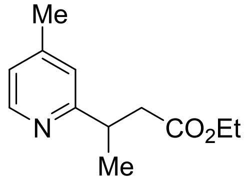

Ethyl 3-(4-methylpyridin-2-yl)butanoate (Table 3, entry 3): following the general procedure (A), the reaction of 2-bromo-4-methylpyridine (171.1 mg, 0.99 mmol, 1 equiv), ethyl crotonate (0.62 mL, 4.99 mmol, 5 equiv), [Ir{dF(CF_3)ppy}$_2$(dtbbpy)]PF_6 (22.0 mg, 0.020 mmol, 0.02 equiv) and Hantzsch ester (318.8 mg, 1.26 mmol, 1.3 equiv) provided the product (169.0 mg, 82% yield) as a pale yellow oil after purification by flash column chromatography (hexane:ethyl acetate = 9:1 then 8:1).
^{1}**H NMR** (600 MHz, $CDCl_3$) δ 8.35 (d, J = 5.0 Hz, 1H), 6.97 (s, 1H), 6.90 (d, J = 5.0 Hz, 1H), 4.08 – 4.00 (m, 2H), 3.36 – 3.29 (m, 1H), 2.82 (dd, J = 15.6, 7.6 Hz, 1H), 2.54 (dd, J = 15.6, 7.2 Hz, 1H), 2.29 (s, 3H), 1.28 (d, J = 6.9 Hz, 3H), 1.15 (t, J = 7.1 Hz, 3H).
^{13}**C NMR** (125 MHz, $CDCl_3$) δ 172.3, 163.9, 148.6, 147.1, 122.5, 122.1, 59.8, 40.6, 37.7, 20.7, 20.5, 13.9.
FTIR (neat): 2975, 1731, 1605, 1561, 1460, 1369, 1177, 1158 cm^{-1}.
HRMS (NSI) *m/z*: $[M+H]^+$ calcd. for $C_{12}H_{18}NO_2$, 208.1332; found, 208.1331.

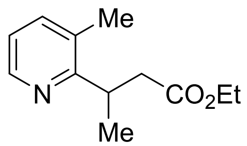

Ethyl 3-(3-methylpyridin-2-yl)butanoate (Table 3, entry 4): following the general procedure (A), the reaction of 2-iodo-3-methylpyridine (215.6 mg, 0.98 mmol, 1 equiv), ethyl crotonate (0.62 mL, 4.99 mmol, 5 equiv), [Ir{dF(CF_3)ppy}$_2$(dtbbpy)]PF_6 (5.7 mg, 0.0051 mmol, 0.005 equiv) and Hantzsch ester (321.6 mg, 1.28 mmol, 1.3 equiv) provided the product (107.5 mg, 53% yield) as a pale yellow oil after purification by flash column chromatography (dichloromethane:diethyl ether = 50:1 then 10:1).

^{1}H NMR (600 MHz, $CDCl_3$) δ 8.35 (d, J = 4.7 Hz, 1H), 7.37 (d, J = 7.6 Hz, 1H), 6.98 (dd, J = 7.6, 4.8 Hz, 1H), 4.06 – 3.96 (m, 2H), 3.63 – 3.55 (m, 1H), 2.96 (dd, J = 16.1, 8.3 Hz, 1H), 2.60 (dd, J = 16.1, 6.4 Hz, 1H), 2.36 (s, 3H), 1.22 (d, J = 6.9 Hz, 3H), 1.13 (t, J = 7.1 Hz, 3H).

^{13}C NMR (125 MHz, $CDCl_3$) δ 172.9, 162.5, 146.6, 137.6, 130.3, 121.0, 60.0, 40.1, 33.1, 20.1, 18.5, 14.0.

FTIR (neat): 2977, 1731, 1586, 1574, 1450, 1369, 1181, 1161 cm^{-1}.

HRMS (NSI) *m/z*: $[M+H]^+$ calcd. for $C_{12}H_{18}NO_2$, 208.1332; found, 208.1331.

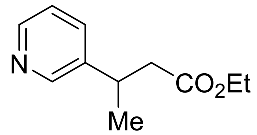

Ethyl 3-(pyridin-3-yl)butanoate (Table 3, entry 5): following the general procedure (A), the reaction of 3-iodopyridine (194.9 mg, 0.95 mmol, 1 equiv), ethyl crotonate (0.31 mL, 2.49 mmol, 5 equiv), [Ir{dF(CF_3)ppy}$_2$(dtbbpy)]PF_6 (5.9 mg, 0.0051 mmol, 0.006 equiv) and Hantzsch ester (317.2 mg, 1.25 mmol, 1.3 equiv) provided the product (95.7 mg, 52% yield) as a pale yellow solid after purification by flash column chromatography (hexane:diethyl ether = 9:1 then 1:1). The physical property and spectrum data match the reported value.[8]

^{1}H NMR (600 MHz, $CDCl_3$) δ 8.47 (d, J = 2.0 Hz, 1H), 8.44 – 8.42 (m, 1H), 7.52 (dt, J = 7.9, 1.9 Hz, 1H), 7.20 (dd, J = 7.8, 4.8 Hz, 1H), 4.04 (q, J = 7.1 Hz, 2H), 3.32 – 3.24 (m, 1H), 2.58 – 2.55 (m, 2H), 1.30 (d, J = 7.0 Hz, 3H), 1.15 (t, J = 7.1 Hz, 3H).

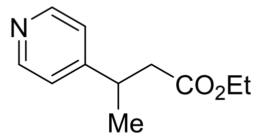

Ethyl 3-(pyridin-4-yl)butanoate (Table 3, entry 6): following the general procedure (A), the reaction of 4-iodopyridine (203.3 mg, 0.99 mmol, 1 equiv), ethyl crotonate (0.62 mL, 4.99 mmol, 5 equiv), [Ir{dF(CF_3)ppy}$_2$(dtbbpy)]PF_6 (5.8 mg, 0.0052 mmol, 0.005 equiv) and Hantzsch ester (313.1 mg, 1.24 mmol, 1.2 equiv) provided the product (92.3 mg, 48% yield) as a pale yellow oil after purification by flash column chromatography (dichloromethane:diethyl ether = 50:1 then 10:1).

[8] Sainsbury, M.; Weerasinghe, D.; Dolman, D. *J. Chem Soc., Perkin Trans. 1.* **1982**, 587-590.

^{1}H NMR (600 MHz, $CDCl_3$) δ 8.49 (d, J = 5.5 Hz, 2H), 7.12 (d, J = 6.0 Hz, 2H), 4.05 (q, J = 7.1 Hz, 2H), 3.30 – 3.19 (m, 1H), 2.59 (dd, J = 15.5, 7.5 Hz, 1H), 2.53 (dd, J = 15.4, 7.6 Hz, 1H), 1.28 (d, J = 7.0 Hz, 3H), 1.16 (t, J = 7.1 Hz, 3H).
^{13}C NMR (125 MHz, $CDCl_3$) δ 171.5, 154.5, 149.7, 122.1, 60.4, 41.7, 35.7, 21.0, 14.0.
FTIR (neat): 2976, 1731, 1599, 1559, 1457, 1415, 1371, 1284, 1173 cm^{-1}.
HRMS (NSI) *m/z*: $[M+H]^+$ calcd. for $C_{11}H_{12}NO_2$, 194.1176; found, 196.1174.

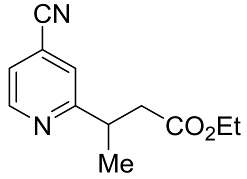

Ethyl 3-(4-cyanopyridin-2-yl)butanoate (Table 3, entry 7): following the general procedure (B), the reaction of 4-cyano-2-iodopyridine (228.6 mg, 0.99 mmol, 1 equiv), ethyl crotonate (0.37 mL, 2.98 mmol, 3 equiv), $[Ir\{dF(CF_3)ppy\}_2(dtbbpy)]PF_6$ (11.3 mg, 0.010 mmol, 0.01 equiv), 2,4,6-trimethylaniline (0.14 mL, 1.0 mmol, 1 equiv) and sodium formate (208.6 mg, 3.07 mmol, 3.1 equiv) provided the product (112.5 mg, 52% yield) as a yellow oil after purification by flash column chromatography (hexane:ethyl acetate = 9:1 then 8:2).
^{1}H NMR (400 MHz, $CDCl_3$) δ 8.68 (dd, J = 5.0, 0.9 Hz, 1H), 7.42 – 7.41 (m, 1H), 7.33 (dd, J = 5.0, 1.5 Hz, 1H), 4.08 – 3.99 (m, 2H), 3.49 – 3.39 (m, 1H), 2.88 (dd, J = 16.1, 8.3 Hz, 1H), 2.59 (dd, J = 16.1, 6.3 Hz, 1H), 1.31 (d, J = 7.0 Hz, 3H), 1.16 (t, J = 7.1 Hz, 3H).
^{13}C NMR (125 MHz, $CDCl_3$) δ 171.9, 166.1, 150.0, 123.7, 122.7, 120.5, 116.5, 60.2, 40.1, 37.9, 20.5, 14.0.
FTIR (neat): 2978, 2238, 1729, 1595, 1551, 1474, 1398, 1370, 1279, 1179 cm^{-1}.
HRMS (NSI) *m/z*: $[M+H]^+$ calcd. for $C_{12}H_{15}N_2O_2$, 219.1128; found, 219.1127.

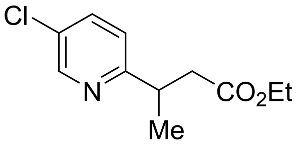

Ethyl 3-(5-chloropyridin-2-yl)butanoate (Table 3, entry 8): following the general procedure (A), the reaction of 5-chloro-2-iodopyridine (233.7 mg, 0.98 mmol, 1 equiv), ethyl crotonate (0.62 mL, 4.99 mmol, 5 equiv), $[Ir\{dF(CF_3)ppy\}_2(dtbbpy)]PF_6$ (6.2 mg, 0.0055 mmol, 0.006 equiv) and Hantzsch ester (314.2 mg, 1.24 mmol, 1.3 equiv) provided the product (129.4 mg, 58% yield) as a pale yellow oil after purification by flash column chromatography (dichloromethane:diethyl ether = 100:1).
^{1}H NMR (600 MHz, $CDCl_3$) δ 8.46 (d, J = 2.5 Hz, 1H), 7.55 (dd, J = 8.3, 2.5 Hz, 1H), 7.13 (d, J = 8.3 Hz, 1H), 4.08 – 4.01 (m, 2H), 3.42 – 3.33 (m, 1H), 2.82 (dd, J = 15.9, 8.0 Hz, 1H), 2.56 (dd, J = 15.9, 6.7 Hz, 1H), 1.28 (d, J = 8.2 Hz, 3H), 1.16 (t, J = 7.1 Hz, 3H).
^{13}C NMR (125 MHz, $CDCl_3$) δ 171.2, 157.9, 151.6, 149.6, 122.6, 121.0, 60.5, 41.5, 35.5, 20.9, 14.0.
FTIR (neat): 2976, 1731, 1579, 1560, 1471, 1369, 1349, 1275, 1161, 1112, 1013 cm^{-1}.
HRMS (NSI) *m/z*: $[M+H]^+$ calcd. for $C_{11}H_{15}Cl_1N_1O_3$, 228.0786; found, 228.0786.

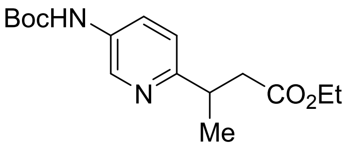

Ethyl 3-(5-((*tert*-butoxycarbonyl)amino)pyridin-2-yl)butanoate (Table 3, entry 9): following the general procedure (A), the reaction of *tert*-butyl (6-iodopyridin-3-yl)carbamate (320.2 mg, 1.00 mmol, 1 equiv), ethyl crotonate (0.62 mL, 4.99 mmol, 5 equiv), [Ir{dF(CF_3)ppy}$_2$(dtbbpy)]PF_6 (5.3 mg, 0.0047 mmol, 0.005 equiv) and Hantzsch ester (315.5 mg, 1.25 mmol, 1.3 equiv) provided the product (148.9 mg, 48% yield) as a pale yellow oil after purification by flash column chromatography (hexane:ethyl acetate = 4:1 then 1:1).

^{1}H NMR (600 MHz, $CDCl_3$) δ 8.29 (d, J = 2.5 Hz, 1H), 7.88 (s, 1H), 7.11 (d, J = 8.5 Hz, 1H), 6.49 (s, 1H), 4.08 – 4.01 (m, 2H), 3.41 – 3.29 (m, 1H), 2.79 (dd, J = 16.0, 8.0 Hz, 1H), 2.53 (dd, J = 15.6, 7.2 Hz, 1H), 1.49 (s, 9H), 1.27 (d, J = 7.8 Hz, 3H), 1.16 (t, J = 7.1 Hz, 3H).

^{13}C NMR (125 MHz, $CDCl_3$) δ 172.5, 158.4, 152.9, 139.6, 133.3, 126.4, 121.5, 80.7, 60.1, 40.9, 37.3, 28.1, 20.6, 14.0.

FTIR (neat): 3333, 2977, 1724, 1588, 1526, 1491, 1390, 1367, 1246, 1154 cm^{-1}.

HRMS (NSI) *m/z*: $[M+H]^+$ calcd. for $C_{16}H_{25}N_2O_4$, 309.1809; found, 309.1807.

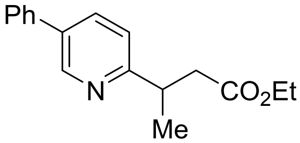

Ethyl 3-(5-phenylpyridin-2-yl)butanoate (Table 3, entry 10): following the general procedure (A), the reaction of 2-bromo-5-phenylpyridine (227.1 mg, 0.97 mmol, 1 equiv), ethyl crotonate (0.62 mL, 4.99 mmol, 5 equiv), [Ir{dF(CF_3)ppy}$_2$(dtbbpy)]PF_6 (5.7 mg, 0.0051 mmol, 0.005 equiv) and Hantzsch ester (320.4 mg, 1.26 mmol, 1.3 equiv) provided the product (159.0 mg, 61% yield) as a pale yellow oil after purification by flash column chromatography (hexane:diethyl ether = 4:1 then 2:1).

^{1}H NMR (600 MHz, $CDCl_3$) δ 8.75 (d, J = 2.4 Hz, 1H), 7.78 (ddd, J = 8.0, 2.4, 0.7 Hz, 1H), 7.54 (d, J = 8.1 Hz, 2H), 7.45 (t, J = 7.6 Hz, 2H), 7.37 (t, J = 7.4 Hz, 1H), 7.26 – 7.23 (m, 1H), 4.10 – 4.04 (m, 2H), 3.49 – 3.42 (m, 1H), 2.90 (dd, J = 15.7, 7.7 Hz, 1H), 2.61 (dd, J = 15.7, 7.1 Hz, 1H), 1.35 (d, J = 7.0 Hz, 3H), 1.17 (t, J = 7.1 Hz, 3H).

^{13}C NMR (125 MHz, $CDCl_3$) δ 172.5, 163.2, 147.5, 137.8, 134.8, 134.3, 128.9, 127.7, 126.9, 121.7, 60.2, 40.8, 37.7, 20.7, 14.1.

FTIR (neat): 2975, 1731, 1596, 1558, 1476, 1449, 1369, 1278, 1161 cm^{-1}.

HRMS (NSI) *m/z*: $[M+H]^+$ calcd. for $C_{17}H_{20}NO_2$, 270.1489; found, 270.1487.

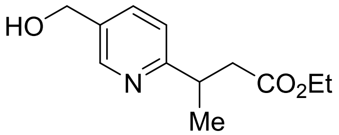

Ethyl 3-(5-(hydroxymethyl)pyridin-2-yl)butanoate (Table 3, entry 11): following the general procedure (A), the reaction of (6-bromo-pyridin-3-yl)methanol (190.0 mg, 1.01 mmol, 1 equiv), ethyl crotonate (0.62 mL, 4.99 mmol, 5 equiv), Ir(dtbbpy)(ppy)$_2$]PF_6 (4.6 mg, 0.0050 mmol, 0.005 equiv) and Hantzsch ester (333.9 mg, 1.32 mmol, 1.3 equiv)

provided the product (167.9 mg, 74% yield) as a pale yellow oil after purification by flash column chromatography (hexane:ethyl acetate = 1:1 then 1:2).
^{1}H NMR (400 MHz, $CDCl_3$) δ 8.47 (d, J = 1.9 Hz, 1H), 7.62 (dd, J = 8.0, 2.3 Hz, 1H), 7.17 (d, J = 8.0 Hz, 1H), 4.66 (s, 2H), 4.10 – 3.98 (m, 2H), 3.46 – 3.34 (m, 1H), 2.84 (dd, J = 15.7, 7.7 Hz, 1H), 2.57 (dd, J = 15.7, 7.0 Hz, 1H), 1.77 (br.s, 1H), 1.29 (d, J = 7.0 Hz, 3H), 1.16 (t, J = 7.1 Hz, 3H).
^{13}C NMR (125 MHz, $CDCl_3$) δ 172.4, 163.1, 147.5, 135.7, 134.4, 121.5, 61.7, 60.2, 40.7, 37.6, 20.5, 13.9.
FTIR (neat): 3366 (br.), 2976, 1729, 1602, 1571, 1489, 1458, 1370, 1278, 1163 cm^{-1}.
HRMS (NSI) *m/z*: $[M+H]^+$ calcd. for $C_{12}H_{18}NO_3$, 224.1281; found, 224.1281.

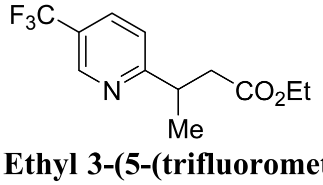

Ethyl 3-(5-(trifluoromethyl)pyridin-2-yl)butanoate (Table 3, entry 12): following the general procedure (A), the reaction of 2-iodo-5-trifluoromethylpyridine (271.3 mg, 0.99 mmol, 1 equiv), ethyl crotonate (0.62 mL, 4.99 mmol, 5 equiv), $[Ir\{dF(CF_3)ppy\}_2(dtbbpy)]PF_6$ (5.7 mg, 0.0051 mmol, 0.005 equiv) and Hantzsch ester (306.8 mg, 1.21 mmol, 1.2 equiv) provided the product (176.4 mg, 68% yield) as a pale yellow oil after purification by flash column chromatography (dichloromethane:diethyl ether = 100:1 then 50:1).
^{1}H NMR (600 MHz, $CDCl_3$) δ 8.77 (br.s, 1H), 7.81 (dd, J = 8.2, 2.4 Hz, 1H), 7.31 (d, J = 8.2 Hz, 1H), 4.08 – 4.01 (m, 2H), 3.51 – 3.40 (m, 1H), 2.90 (dd, J = 16.1, 8.2 Hz, 1H), 2.60 (dd, J = 16.0, 6.4 Hz, 1H), 1.32 (d, J = 7.0 Hz, 3H), 1.17 (d, J = 7.1 Hz, 3H).
^{13}C NMR (125 MHz, $CDCl_3$) δ 172.1, 168.4, 146.0 (q, J = 4.1 Hz, δ = 146.06, 146.02, 145.99, 145.96), 133.3 (q, J = 3.5 Hz, δ = 133.35, 133.33, 133.30, 133.27), 124.3 (q, J = 3.3 Hz, δ = 126.82, 124.66, 122.50, 120.34), 123.6 (q, J = 270.4 Hz, δ = 124.66, 124.41, 124.15, 123.89), 121.8, 60.2, 40.3, 38.0, 20.5, 13.9.
^{19}F NMR ($CDCl_3$) δ -62.28.
FTIR (neat): 2979, 1734, 1607, 1574, 1460, 1400, 1371, 1328, 1160, 1130 cm^{-1}.
HRMS (NSI) *m/z*: $[M+H]^+$ calcd. for $C_{12}H_{15}NO_2F_3$, 262.1049; found, 262.1047.

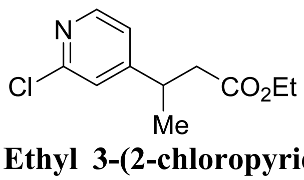

Ethyl 3-(2-chloropyridin-4-yl)butanoate (Table 3, entry 13): following the general procedure (A), the reaction of 2-chloro-4-iodopyridine (240.1 mg, 1.00 mmol, 1 equiv), ethyl crotonate (0.62 mL, 4.99 mmol, 5 equiv), $[Ir\{dF(CF_3)ppy\}_2(dtbbpy)]PF_6$ (7.5 mg, 0.0067 mmol, 0.007 equiv) and Hantzsch ester (317.2 mg, 1.25 mmol, 1.3 equiv) provided the product (120.2 mg, 53% yield) as a pale yellow oil after purification by flash column chromatography (hexane:ethyl acetate = 9:1 then 4:1).
^{1}H NMR (600 MHz, $CDCl_3$) δ 8.27 (d, J = 5.1 Hz, 1H), 7.17 (s, 1H), 7.06 (d, J = 5.1 Hz, 1H), 4.07 (q, J = 7.1 Hz, 2H), 3.30 – 3.19 (m, 1H), 2.60 – 2.51 (m, 2H), 1.28 (d, J = 7.0 Hz, 3H), 1.17 (t, J = 7.1 Hz, 3H).

^{13}C NMR (125 MHz, $CDCl_3$) δ 171.1, 157.9, 151.6, 149.6, 122.6, 121.0, 60.5, 41.4, 35.5, 20.9, 14.0.
FTIR (neat): 2976, 1729, 1592, 1545, 1466, 1392, 1370, 1278, 1203, 1131 cm^{-1}.
HRMS (NSI) *m/z*: $[M+H]^+$ calcd. for $C_{11}H_{15}Cl_1N_1O_3$, 228.0786; found, 228.0787.

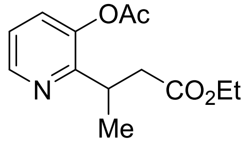

Ethyl 3-(3-acetoxypyridin-2-yl)butanoate (Table 3, entry 14): following the general procedure (A), the reaction of 2-iodopyridin-3-yl acetate (260.6 mg, 0.99 mmol, 1 equiv), ethyl crotonate (0.62 mL, 4.99 mmol, 5 equiv), [Ir{dF(CF_3)ppy}$_2$(dtbbpy)]PF_6 (5.6 mg, 0.0050 mmol, 0.005 equiv) and Hantzsch ester (330.2 mg, 1.30 mmol, 1.3 equiv) provided the product (145.8 mg, 58% yield) as a pale yellow oil after purification by flash column chromatography (hexane:diethyl ether = 4:1 then 2:1).
^{1}H NMR (400 MHz, $CDCl_3$) δ 8.41 (dd, J = 4.7, 1.5 Hz, 1H), 7.36 (dd, J = 8.2, 1.5 Hz, 1H), 7.15 (dd, J = 8.1, 4.7 Hz, 1H), 4.10 – 3.98 (m, 2H), 3.63 – 3.53 (m, 1H), 2.85 (dd, J = 15.9, 7.6 Hz, 1H), 2.57 (dd, J = 15.9, 7.0 Hz, 1H), 2.35 (s, 3H), 1.23 (d, J = 6.9 Hz, 3H), 1.15 (t, J = 7.1 Hz, 3H).
^{13}C NMR (100 MHz, $CDCl_3$) δ 172.4, 168.9, 156.4, 146.5, 144.3, 130.0, 121.8, 60.1, 39.8, 31.1, 20.8, 19.6, 13.9.
FTIR (neat): 2979, 1768, 1730, 1593, 1545, 1441, 1370, 1290, 1192, 1156, 1091 cm^{-1}.
HRMS (NSI) *m/z*: $[M+H]^+$ calcd. for $C_{13}H_{18}NO_4$, 252.1230; found, 252.1229.

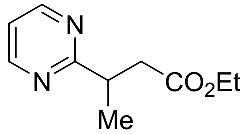

Ehyl 3-(pyrimidin-2-yl)butanoate (Table 3, entry 15): following the general procedure (B), the reaction of 2-bromopyrimidine (160.5 mg, 1.01 mmol, 1 equiv), ethyl crotonate (0.37 mL, 2.98 mmol, 3 equiv), [Ir{dF(CF_3)ppy}$_2$(dtbbpy)]PF_6 (11.7 mg, 0.010 mmol, 0.01 equiv), 2,4,6-trimethylaniline (0.14 mL, 1.0 mmol, 1 equiv) and sodium formate (223.7 mg, 3.29 mmol, 3.3 equiv) provided the product (77.4 mg, 39% yield) as a pale yellow oil after purification by flash column chromatography (hexane:ethyl acetate = 4:1 then 1:1).
^{1}H NMR (600 MHz, $CDCl_3$) δ 8.65 (d, J = 4.9 Hz, 2H), 7.09 (t, J = 4.9 Hz, 1H), 4.08 – 4.02 (m, 2H), 3.60 – 3.51 (m, 1H), 2.98 (dd, J = 16.0, 8.3 Hz, 1H), 2.61 (dd, J = 16.0, 6.5 Hz, 1H), 1.35 (d, J = 7.1 Hz, 3H), 1.15 (t, J = 7.1 Hz, 3H).
^{13}C NMR (125MHz, $CDCl_3$) δ 173.1, 172.3, 156.8, 118.5, 60.1, 39.7, 39.2, 20.0, 14.0.
FTIR (neat): 2976, 1731, 1571, 1561, 1463, 1424, 1369, 1282, 1258, 1177 cm^{-1}.
HRMS (NSI) *m/z*: $[M+H]^+$ calcd. for $C_{10}H_{15}N_2O_2$, 195.1128; found, 195.1128.

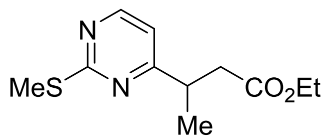

Ethyl 3-(2-(methylthio)pyrimidin-4-yl)butanoate (Table 3, entry 16): following the general procedure (A), the reaction of 4-iodo-2-(methylthio)pyrimidine (248.0 mg, 0.98

mmol, 1 equiv), ethyl crotonate (0.62 mL, 4.99 mmol, 5 equiv), [Ir{dF(CF_3)ppy}$_2$(dtbbpy)]PF_6 (6.4 mg, 0.0057 mmol, 0.006 equiv) and Hantzsch ester (318.5 mg, 1.26 mmol, 1.3 equiv) provided the product (160.9 mg, 68% yield) as a pale yellow oil after purification by flash column chromatography (hexane:diethyl ether = 4:1 then 2:1).

^{1}H NMR (600 MHz, $CDCl_3$) δ 8.37 (d, J = 5.1 Hz, 1H), 6.83 (d, J = 5.1 Hz, 1H), 4.09 – 4.03 (m, 2H), 3.33 – 3.25 (m, 1H), 2.88 (dd, J = 16.1, 7.9 Hz, 1H), 2.56 – 2.51 (m, 4H), 1.28 (d, J = 7.0 Hz, 3H), 1.18 (t, J = 7.1 Hz, 3H).

^{13}C NMR (125 MHz, $CDCl_3$) δ 172.8, 171.9, 171.8, 156.8, 114.3, 60.1, 39.3, 37.2, 19.8, 13.9, 13.7.

FTIR (neat): 2976, 1730, 1562, 1542, 1424, 1367, 1342, 1326, 1200, 1182, 1160 cm^{-1}.

HRMS (NSI) *m/z*: $[M+H]^+$ calcd. for $C_{11}H_{17}N_2O_2S$, 241.1005; found, 241.1005.

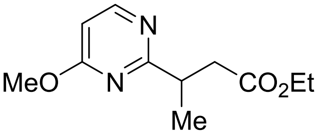

Ethyl 3-(4-methoxypyrimidin-2-yl)butanoate (Table 3, entry 17): following the general procedure (A), the reaction of 2-iodo-4-methoxypyrimidine (233.8 mg, 0.99 mmol, 1 equiv), ethyl crotonate (0.62 mL, 4.99 mmol, 5 equiv), Ir(dtbbpy)(ppy)$_2$]PF_6 (4.7 mg, 0.0051 mmol, 0.005 equiv) and Hantzsch ester (310.2 mg, 1.22 mmol, 1.2 equiv) provided the product (152.2 mg, 68% yield) as a pale yellow oil after purification by flash column chromatography (hexane:ethyl acetate = 1:1 then 1:2).

^{1}H NMR (400 MHz, $CDCl_3$) δ 8.32 (d, J = 5.8 Hz, 1H), 6.51 (d, J = 5.8 Hz, 1H), 4.10 – 4.03 (m, 2H), 3.93 (s, 3H), 3.53 – 3.39 (m, 1H), 2.94 (dd, J = 15.9, 8.1 Hz, 1H), 2.56 (dd, J = 15.9, 6.7 Hz, 1H), 1.33 (d, J = 7.0 Hz, 3H), 1.17 (t, J = 7.1 Hz, 3H).

^{13}C NMR (125 MHz, $CDCl_3$) δ 172.8, 172.4, 169.1, 156.8, 105.3, 59.9, 53.1, 39.5, 38.9, 19.7, 14.0.

FTIR (neat): 2979, 1732, 1568, 1473, 1418, 1367, 1327, 1312, 1276, 1174 cm^{-1}.

HRMS (NSI) *m/z*: $[M+H]^+$ calcd. for $C_{11}H_{17}N2O_3$, 225.1234; found, 225.1235.

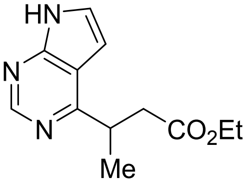

Ethyl 3-(7*H*-pyrrolo[2,3-*d*]pyrimidin-4-yl)butanoate (Table 3, entry 18): following the general procedure (A), the reaction of 4-iodo-7-h-pyrrolo[2,3-d]pyrimidine (119.6 mg, 0.49 mmol, 1 equiv), ethyl crotonate (0.31 mL, 2.49 mmol, 5 equiv), [Ir{dF(CF_3)ppy}$_2$(dtbbpy)]PF_6 (3.1 mg, 0.0051 mmol, 0.006 equiv) and Hantzsch ester (156.2 mg, 0.62 mmol, 1.3 equiv) provided the product (86.5 mg, 76% yield) as a pale yellow solid after purification by flash column chromatography (hexane:diethyl ether = 4:1 then 2:1).

M.P. : 62-64 °C.

^{1}H NMR (600 MHz, $CDCl_3$) δ 10.25 (br.s, 1H), 8.81 (s, 1H), 7.31 (dd, J = 3.4, 2.3 Hz, 1H), 6.66 (dd, J = 3.3, 1.7 Hz, 1H), 4.07 – 3.98 (m, 2H), 3.87 – 3.79 (m, 1H), 3.04 (dd, J

= 16.1, 7.9 Hz, 1H), 2.71 (dd, J = 16.1, 6.8 Hz, 1H), 1.40 (d, J = 7.0 Hz, 3H), 1.13 (t, J = 7.1 Hz, 3H).
^{13}C NMR (125 MHz, $CDCl_3$) δ 172.4, 165.4, 151.5, 150.7, 125.2, 116.7, 99.5, 60.3, 39.6, 35.2, 20.0, 14.0.
FTIR (neat): 3200 (br.), 3133 (br.), 2978, 1731, 1582, 1505, 1465, 1418, 1350, 1286, 1254, 1184 cm^{-1}.
HRMS (NSI) *m/z*: $[M+H]^+$ calcd. for $C_{12}H_{16}N_3O_2$, 234.1237; found, 234.1236.

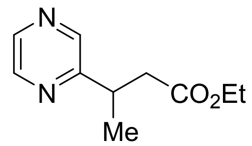

Ethyl 3-(pyrazin-2-yl)butanoate (Table 3, entry 19): following the general procedure (B), the reaction of 2-iodopyrazine (207.0 mg, 1.00 mmol, 1 equiv), ethyl crotonate (0.37 mL, 2.98 mmol, 3 equiv), $[Ir\{dF(CF_3)ppy\}_2(dtbbpy)]PF_6$ (11.1 mg, 0.010 mmol, 0.01 equiv), 2,4,6-trimethylaniline (0.14 mL, 1.0 mmol, 1 equiv) and sodium formate (204.8 mg, 3.01 mmol, 3.0 equiv) provided the product (102.1 mg, 52% yield) as a yellow oil after purification by flash column chromatography (hexane:ethyl acetate = 8:2 then 7:3).
^{1}H NMR (600 MHz, $CDCl_3$) δ 8.48 (d, J = 1.4 Hz, 1H), 8.47 – 8.45 (m, 1H), 8.38 (d, J = 2.5 Hz, 1H), 4.11 – 3.96 (m, 2H), 3.53 – 3.39 (m, 1H), 2.85 (dd, J = 16.0, 8.2 Hz, 1H), 2.60 (dd, J = 16.0, 6.5 Hz, 1H), 1.32 (d, J = 7.0 Hz, 3H), 1.15 (t, J = 7.1 Hz, 3H).
^{13}C NMR (125 MHz, $CDCl_3$) δ 171.8, 159.7, 143.9, 143.8, 142.4, 60.2, 40.1, 35.3, 20.4, 13.9.
FTIR (neat): 2977, 1731, 1526, 1473, 1407, 1371, 1346, 1279, 1179, 1140, 1033 cm^{-1}.
HRMS (NSI) *m/z*: $[M+H]^+$ calcd. for $C_{10}H_{15}N_2O_2$, 195.1128; found, 195.1126.

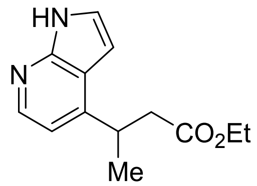

Ethyl 3-(1*H*-pyrrolo[2,3-*b*]pyridin-4-yl)butanoate (Table 3, entry 20): following the general procedure (A), the reaction of 4-bromo-7-azaindole (195.4 mg, 0.99 mmol, 1 equiv), ethyl crotonate (0.62 mL, 4.99 mmol, 5 equiv), $[Ir\{dF(CF_3)ppy\}_2(dtbbpy)]PF_6$ (5.9 mg, 0.0053 mmol, 0.005 equiv) and Hantzsch ester (317.6 mg, 1.25 mmol, 1.3 equiv) provided the product (122.5 mg, 53% yield) as a pale yellow oil after purification by flash column chromatography (hexane:diethyl ether = 1:3 then diethyl ether).
^{1}H NMR (600 MHz, $CDCl_3$) δ 10.45 (br.s, 1H), 8.24 (d, J = 5.0 Hz, 1H), 7.32 (dd, J = 3.6, 1.5 Hz, 1H), 6.93 (d, J = 5.0 Hz, 1H), 6.58 (d, J = 3.3 Hz, 1H), 4.06 (q, J = 7.1 Hz, 2H), 3.75 – 3.68 (m, 1H), 2.79 (dd, J = 15.3, 6.6 Hz, 1H), 2.66 (dd, J = 15.3, 8.4 Hz, 1H), 1.41 (d, J = 7.0 Hz, 3H), 1.15 (t, J = 7.1 Hz, 3H).
^{13}C NMR (100 MHz, $CDCl_3$) δ 172.2, 148.9, 147.4, 142.6, 124.7, 119.1, 112.6, 98.9, 60.4, 41.5, 33.8, 20.5, 14.1.
FTIR (neat): 3143 (br.), 2976, 1730, 1589, 1501, 1445, 1408, 1370, 1273, 1177 cm-1.
HRMS (NSI) *m/z*: $[M+H]^+$ calcd. for $C_{13}H_{17}NO_2$, 233.1285; found, 233.1286.

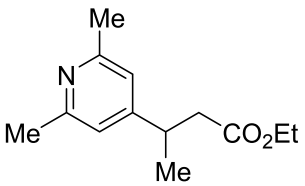

Ethyl 3-(2,6-dimethylpyridin-4-yl)butanoate (Table 3, entry 21): following the general procedure (A), the reaction of 4-bromo-2,6-dimethylpyridine (189.8 mg, 1.02 mmol, 1 equiv), ethyl crotonate (0.62 mL, 4.99 mmol, 5 equiv), Ir(dtbbpy)(ppy)$_2$]PF$_6$ (4.8 mg, 0.0053 mmol, 0.005 equiv) and Hantzsch ester (304.7 mg, 1.20 mmol, 1.2 equiv) provided the product (119.6 mg, 53% yield) as a pale yellow oil after purification by flash column chromatography (hexane:diethyl ether = 1:1 then 1:2).

^{1}H-NMR (600 MHz, CDCl$_3$) δ 6.79 (s, 2H), 4.06 (q, J = 7.1 Hz, 2H), 3.19 – 3.11 (m, 1H), 2.56 (dd, J = 15.4, 7.3 Hz, 1H), 2.52 – 2.43 (m, 7H), 1.24 (d, J = 7.0 Hz, 3H), 1.17 (t, J = 7.1 Hz, 3H).

^{13}C NMR (125 MHz, CDCl$_3$) δ 171.7, 157.5, 155.0, 116.1, 60.2, 41.7, 35.6, 24.2, 21.0, 14.0.

FTIR (neat): 2968, 1732, 1606, 1567, 1426, 1370, 1283, 1252, 1226, 1177, 1160 cm^{-1}.

HRMS (NSI) *m/z*: [M+H]$^+$ calcd. for $C_{13}H_{20}NO_2$, 222.1489; found, 222.1487.

VI. Procedure for determination of Hantzsch ester solubility:

A 1,000 µL aliquot of DMSO or DMSO:H_2O (1:1, 2:1, 3:1, 4:1, 5:1, or 10:1) was delivered to a test tube. The solution was saturated with excess Hantzsch ester (200 mg, 0.79 mmol), and collidine (10 µL, 10.9 mg, 0.090 mmol) was added as an internal standard. The saturated solution was drawn into a syringe through a syringe filter. The syringe filter was removed, and the solution was delivered to a fresh test tube. The Hantzsch ester was oxidized to Hantzsch pyridine by sparging the solution with air for 2 hours. The sample was analyzed by gas chromatography, and integral values were used to calculate the mass of Hantzsch ester dissolved in solution.

VII. equation 1:

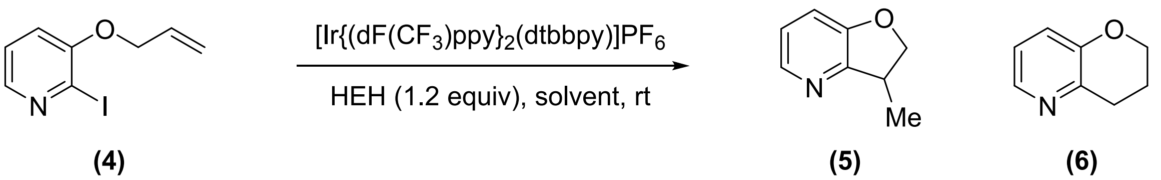

Following the general procedure (A), the reaction of 3-(allyloxy)-2-iodopyridine[9] (compound **4**, 129.8 mg, 0.50 mmol, 1 equiv), [Ir{dF(CF$_3$)ppy}$_2$(dtbbpy)]PF$_6$ (5.1 mg, 0.0045 mmol, 0.01 equiv) and Hantzsch ester (153.3 mg, 0.61 mmol, 1.2 equiv) provided the known cyclization products 3-methyl-2,3-dihydrofuro[3,2-b]pyridine (**5**)[10] and 3,4-dihydro-2H-pyrano[3,2-b]pyridine (**6**)[11] (13% and 33% yield, respectively; yields were

[9] Fantasia, S.; Windisch, J.; Scalone, M. *Adv. Synth. Catal.* **2013**, *355*, 627-631.

[10] Dahlen, A.; Petersson, A.; Hilmersson, G. *Org. Biomol. Chem.* **2003**, *1*, 2423-2426.

determined by ^{1}H NMR using 1,3,5-trimethoxybenzene as internal standard). The compounds were isolated by preparative TLC with (hexanes:ethyl acetate = 1:1). The spectra and physical properties were consistent with previously reported data.[10,11]

3-methyl-2,3-dihydrofuro[3,2-b]pyridine (5):

^{1}H NMR (400 MHz, $CDCl_3$) δ 8.07 – 8.00 (m, 1H), 7.02 – 6.95 (m, 2H), 4.76 (t, J = 9.1 Hz, 1H), 4.15 (dd, J = 8.8, 7.4 Hz, 1H), 3.59 – 3.48 (m, 1H), 1.39 (d, J = 7.0 Hz, 3H).

3,4-dihydro-2H-pyrano[3,2-b]pyridine (6):

^{1}H NMR (400 MHz, $CDCl_3$) δ 8.08 (dd, J = 4.5, 1.3 Hz, 1H), 7.17 – 6.77 (m, 2H), 4.20 – 4.12 (m, 2H), 2.93 (t, J = 6.6 Hz, 2H), 2.12 – 2.06 (m, 2H).

[11] Sliwa, H.; Blondeau, D.; Rydzkowski, R. *J. Heterocyclic Chem.* **1983**, *20*, 1613-161.

VIII. ^{1}H NMR and ^{13}C NMR Spectra

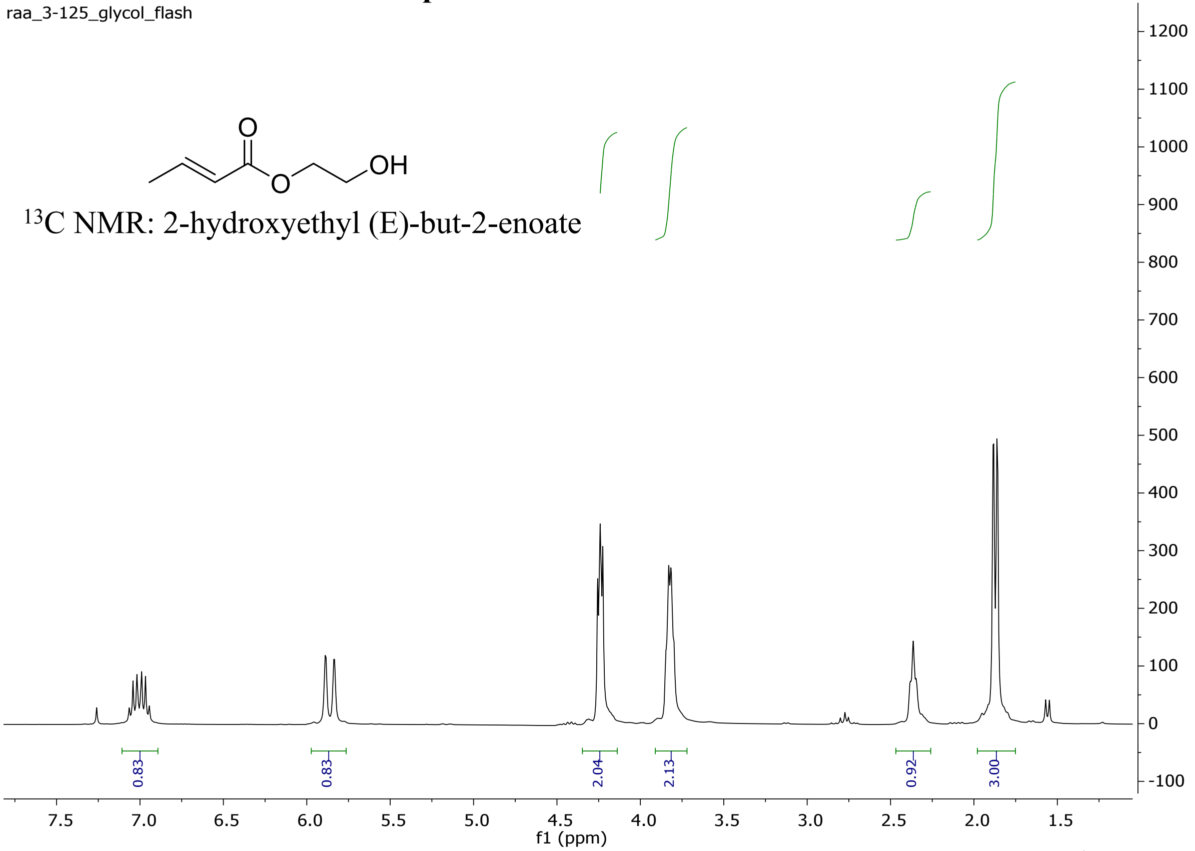

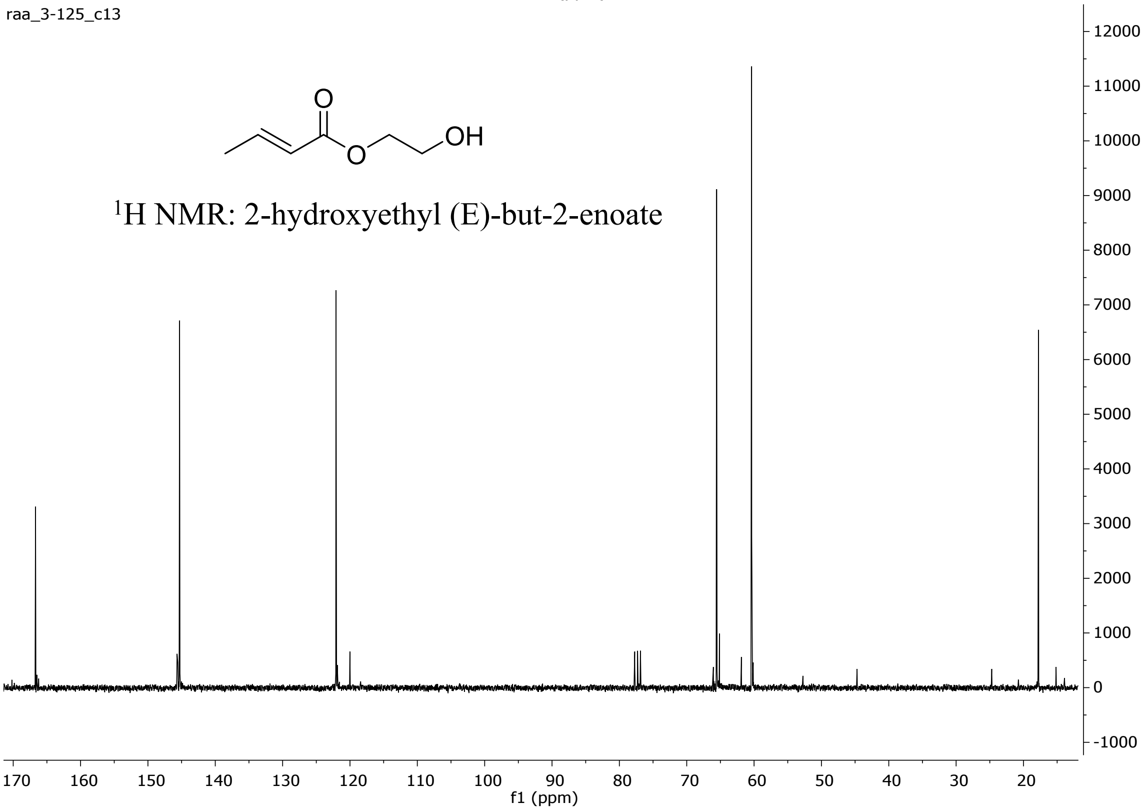

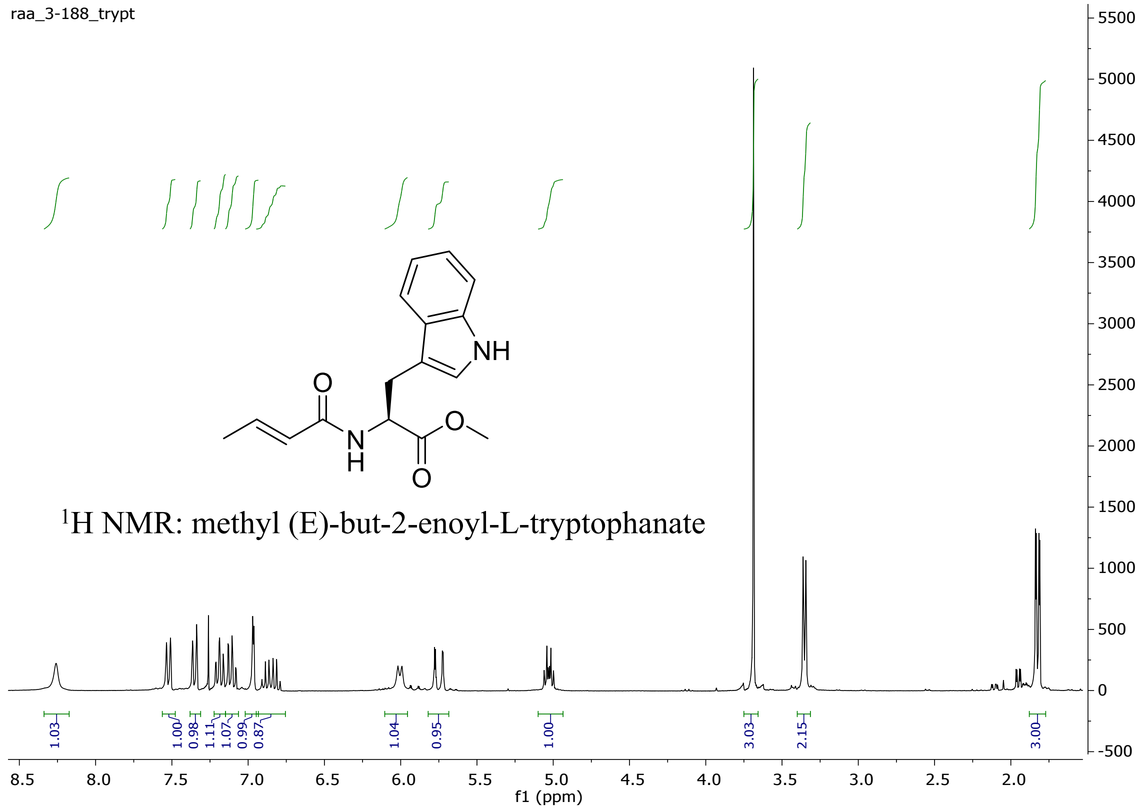
raa_3-188_trypt
NH
O
N
H
O
O
1H NMR: methyl (E)-but-2-enoyl-L-tryptophanate
1.03
1.00
0.98
1.11
1.07
0.99
0.87
1.04
0.95
1.00
3.03
2.15
3.00
f1 (ppm)

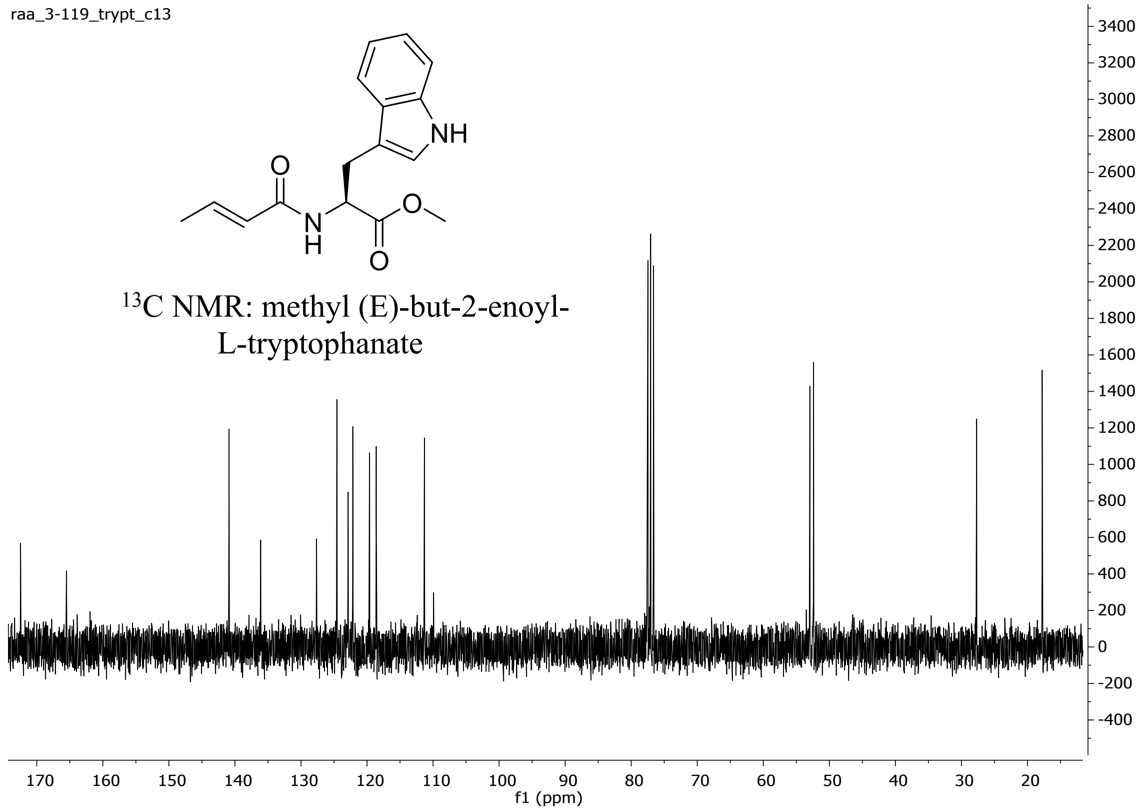
raa_3-119_trypt_c13
NH
O
N
H
O
O
13C NMR: methyl (E)-but-2-enoyl-L-tryptophanate
f1 (ppm)

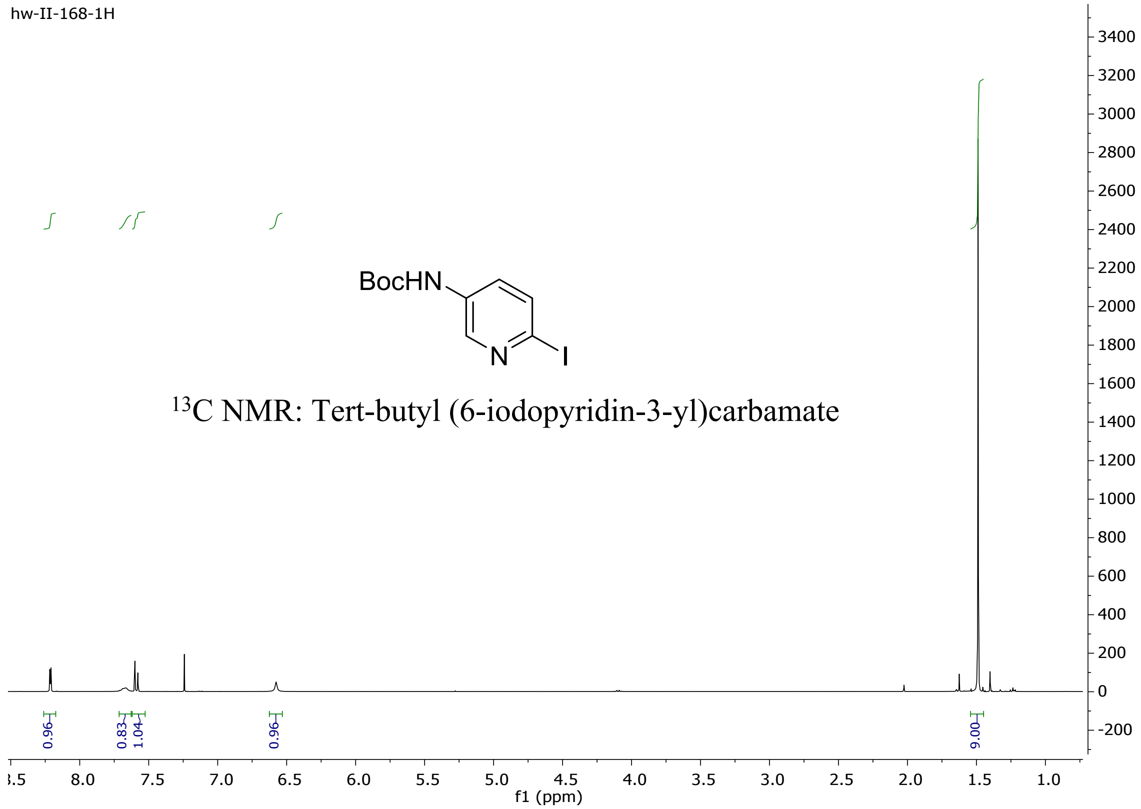
hw-II-168-1H
BocHN
N
I
13C NMR: Tert-butyl (6-iodopyridin-3-yl)carbamate
0.96
0.83
1.04
0.96
9.00
f1 (ppm)

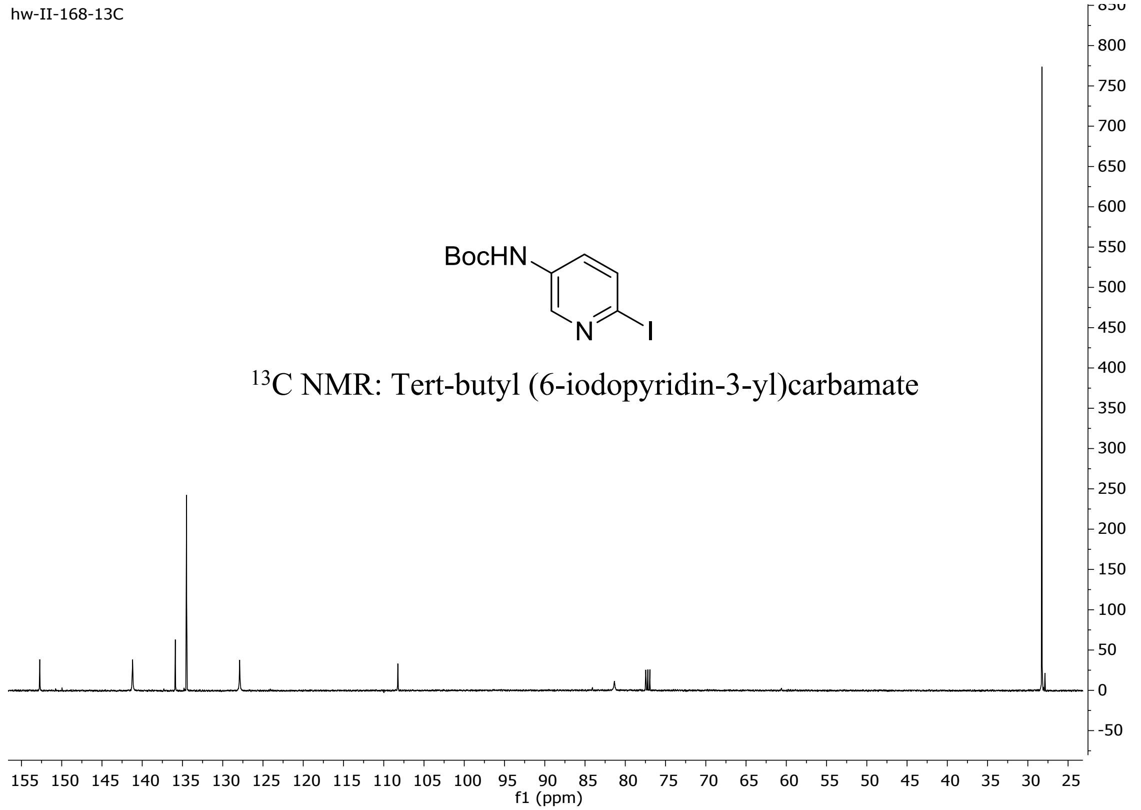
hw-II-168-13C
BocHN
N
I
13C NMR: Tert-butyl (6-iodopyridin-3-yl)carbamate
f1 (ppm)

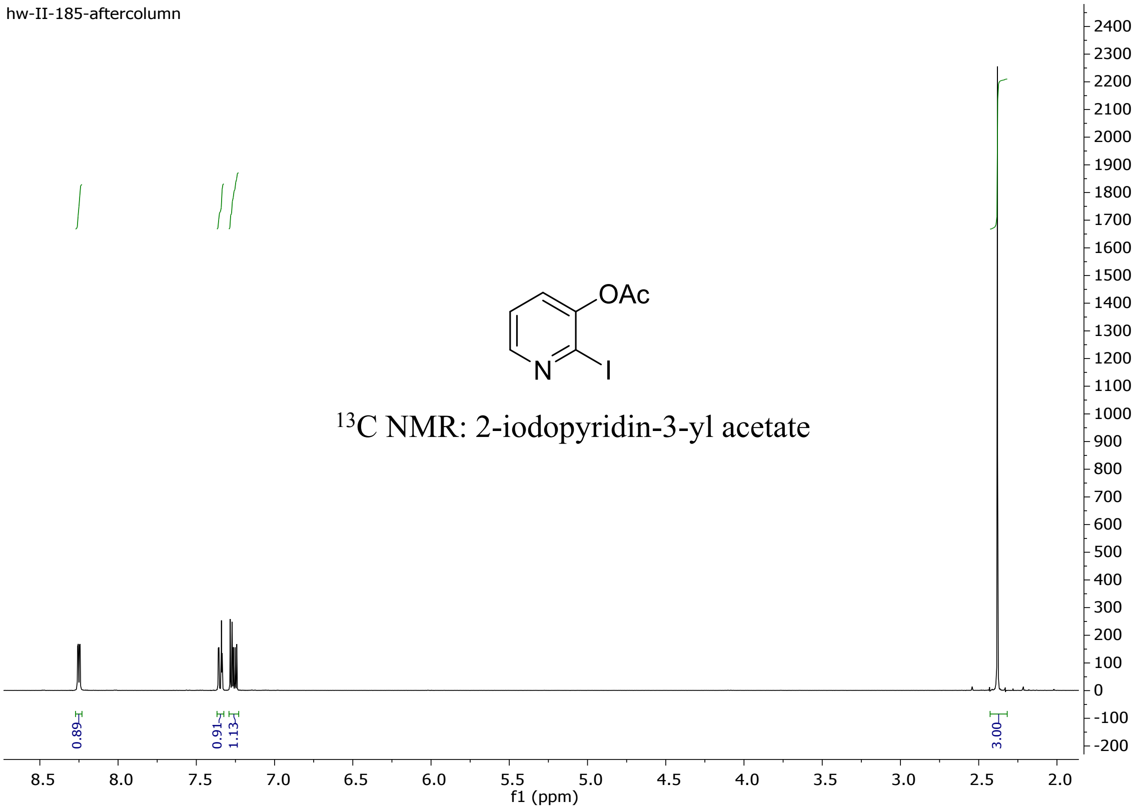
hw-II-185-aftercolumn
OAc
N
I
13C NMR: 2-iodopyridin-3-yl acetate
0.89
0.91
1.13
3.00
f1 (ppm)

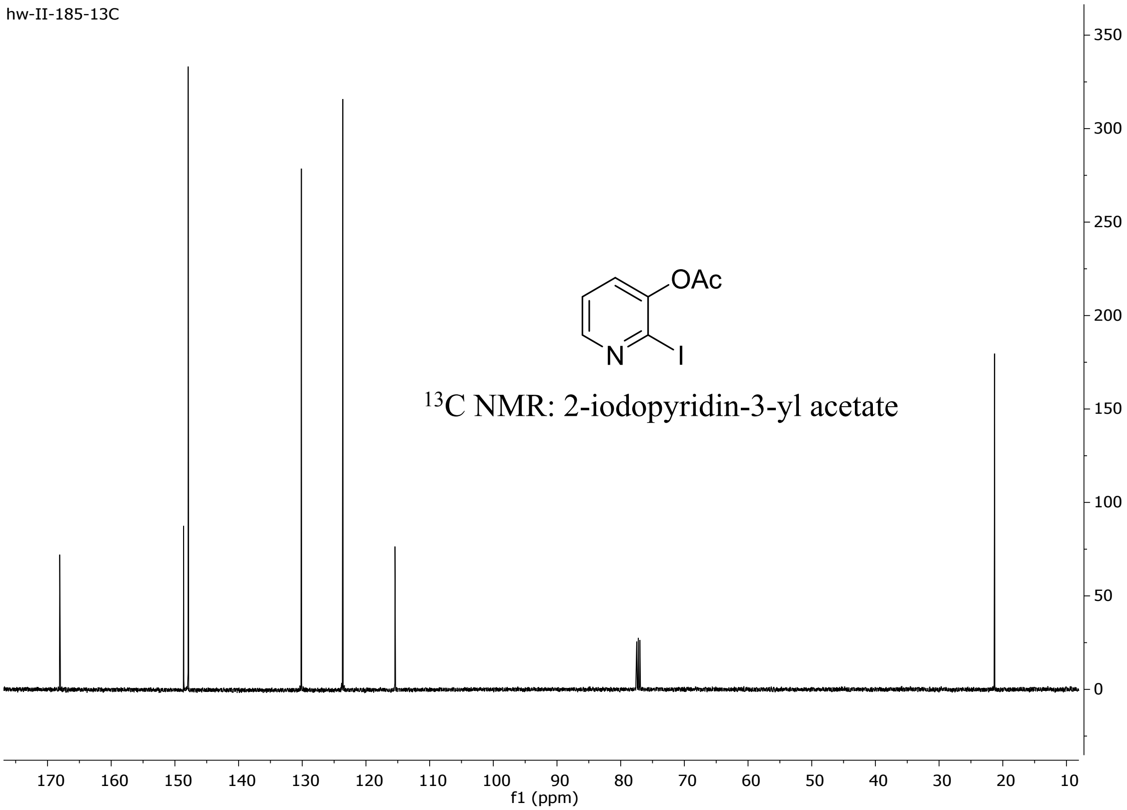
hw-II-185-13C
OAc
N
I
13C NMR: 2-iodopyridin-3-yl acetate
f1 (ppm)

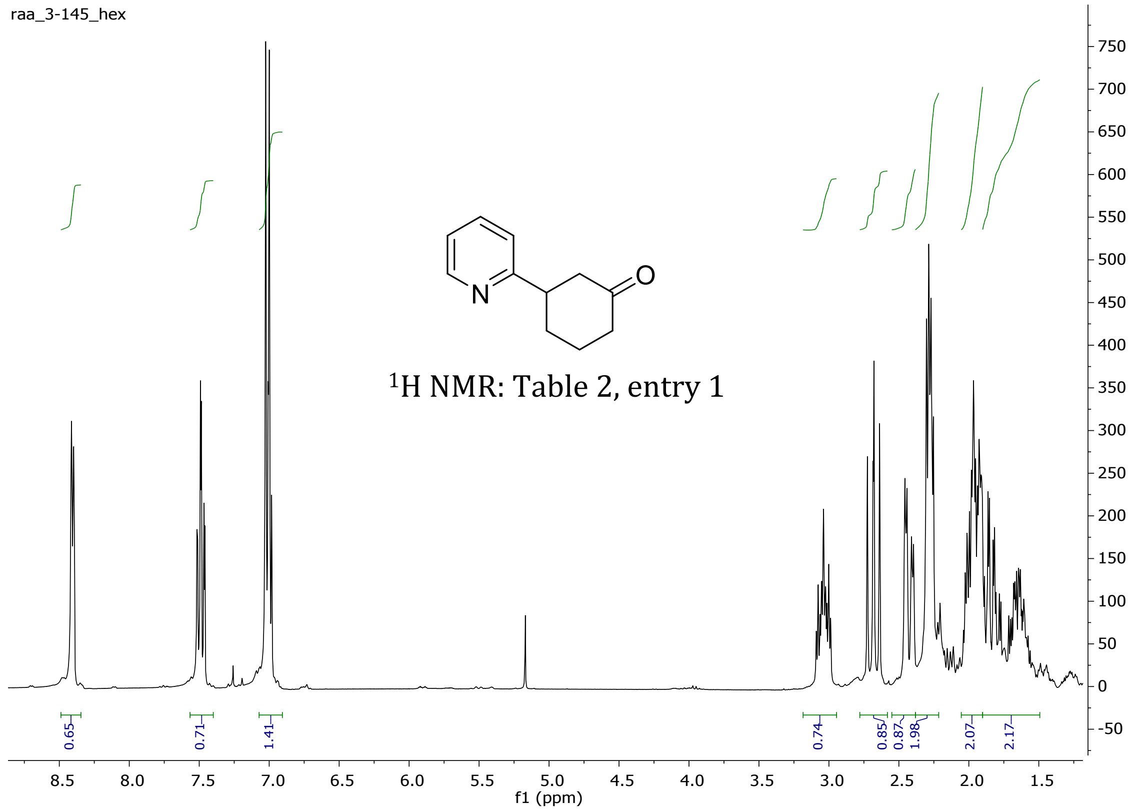
raa_3-145_hex
N
O
1H NMR: Table 2, entry 1
0.65
0.71
1.41
0.74
0.85
0.87
1.98
2.07
2.17
f1 (ppm)

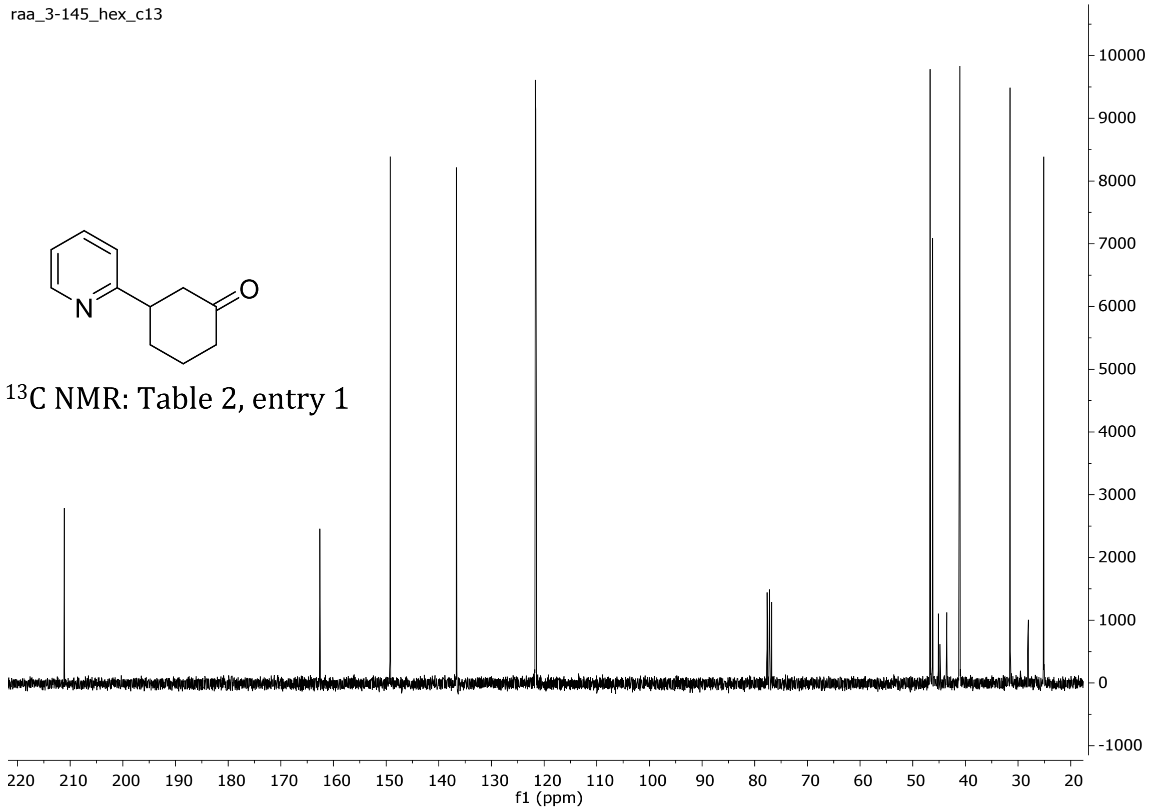
raa_3-145_hex_c13
N
O
13C NMR: Table 2, entry 1
f1 (ppm)

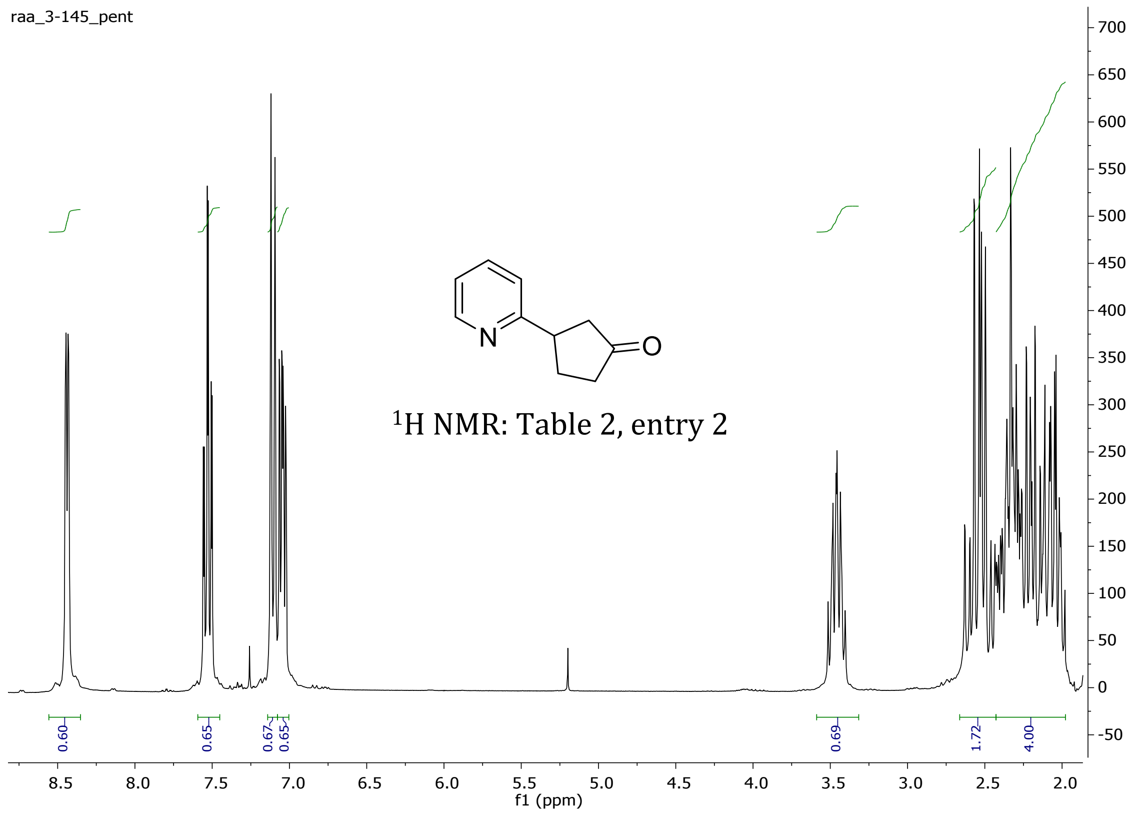
raa_3-145_pent
N
O
¹H NMR: Table 2, entry 2
0.60
0.65
0.67
0.65
0.69
1.72
4.00
f1 (ppm)

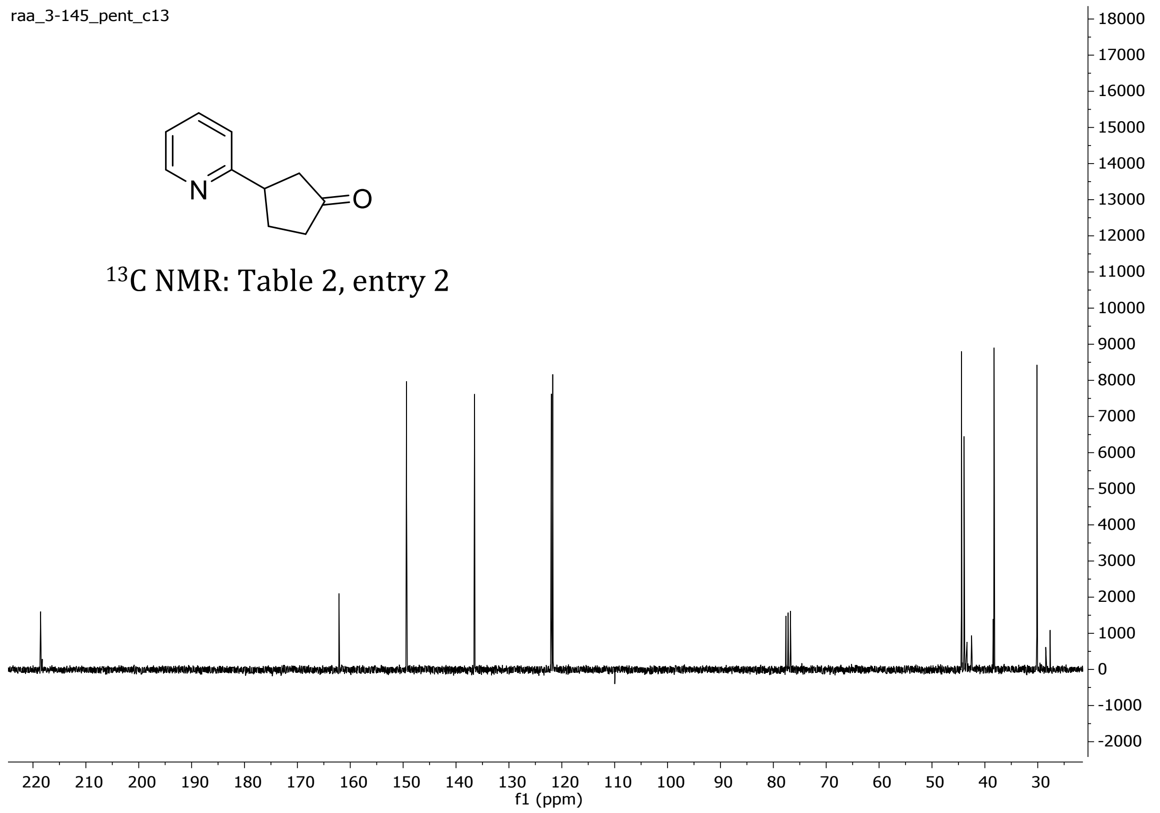
raa_3-145_pent_c13
N
O
¹³C NMR: Table 2, entry 2
f1 (ppm)

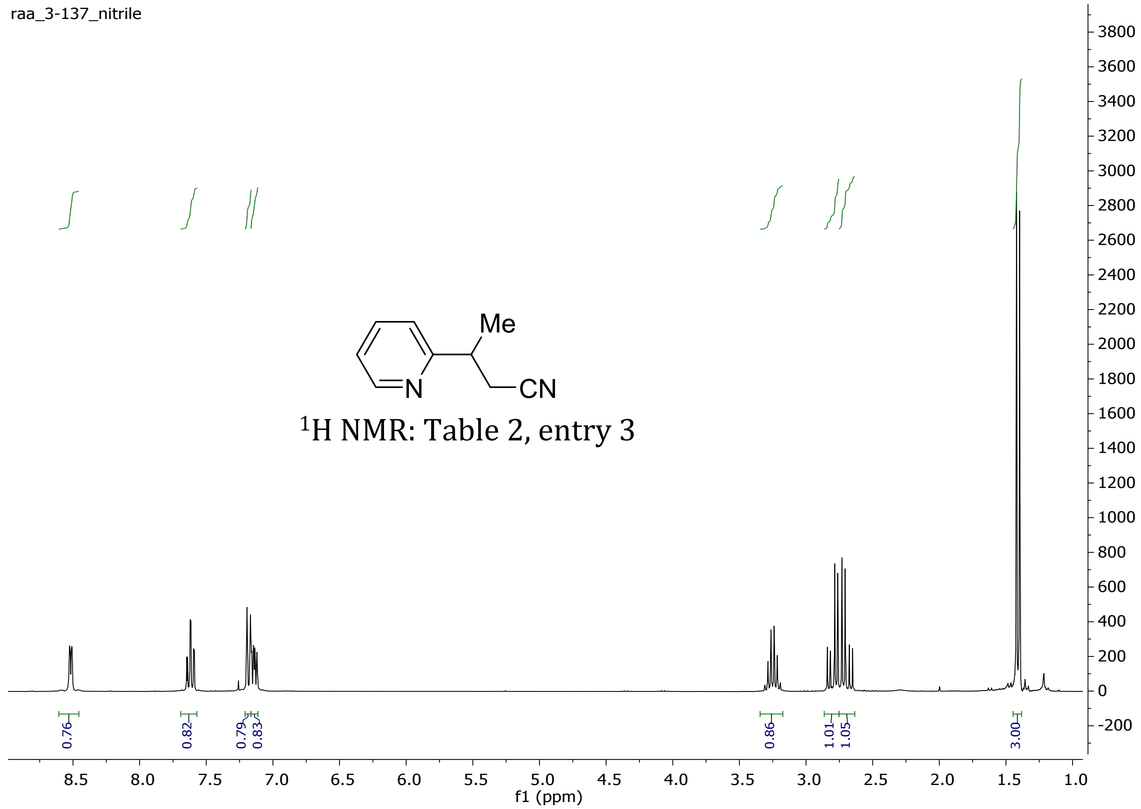
raa_3-137_nitrile
Me
N
CN
0.76
0.82
0.79
0.83
0.86
1.01
1.05
3.00
f1 (ppm)

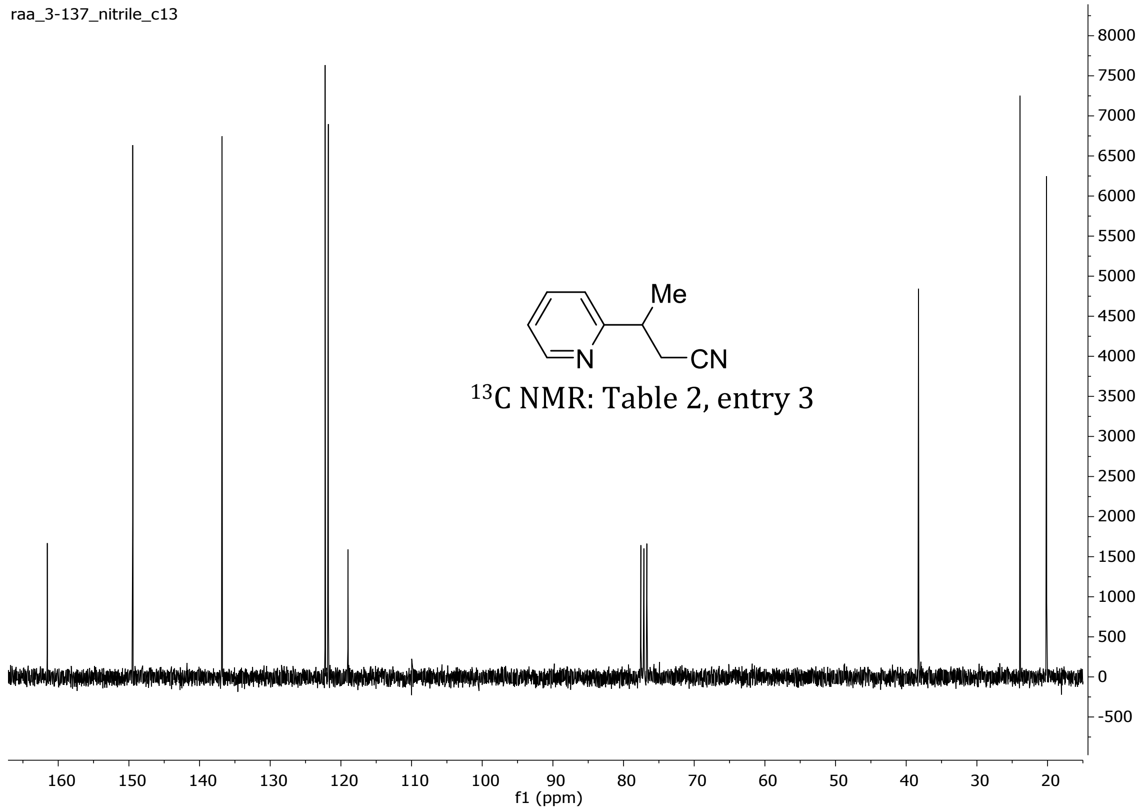
raa_3-137_nitrile_c13
Me
N
CN
f1 (ppm)

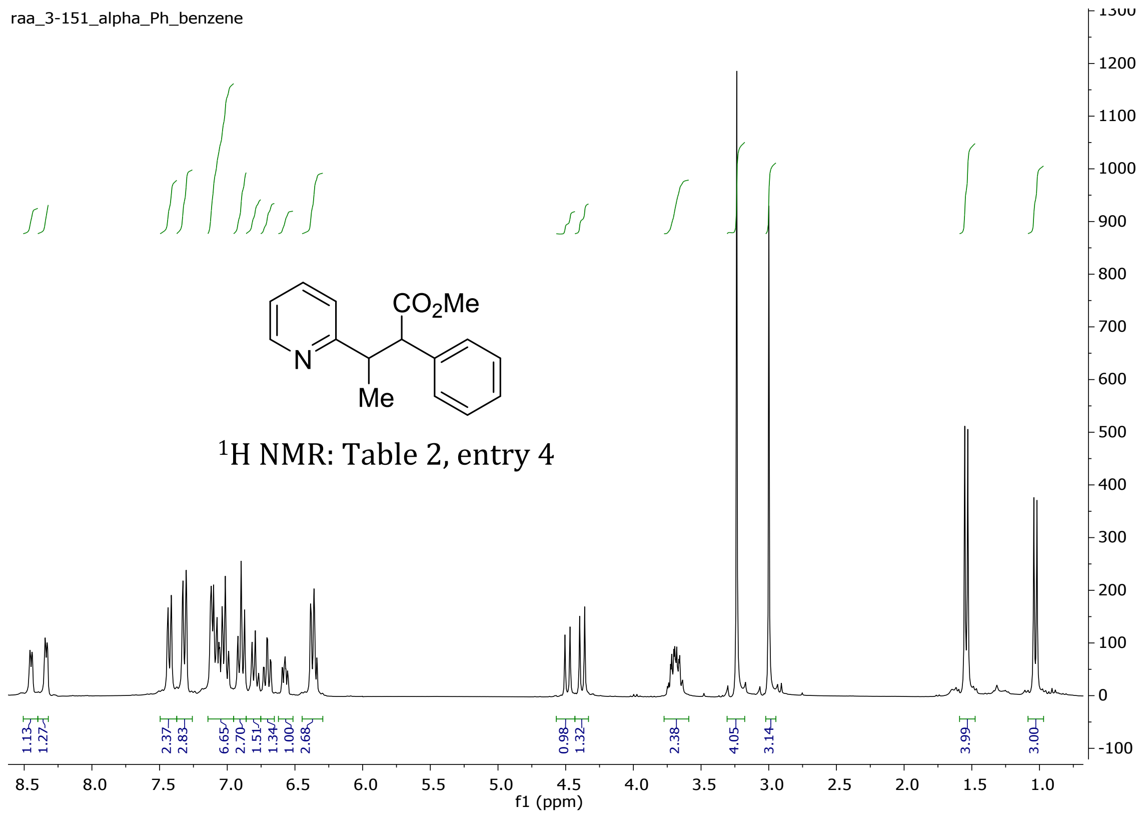
raa_3-151_alpha_Ph_benzene
CO2Me
N
Me
1H NMR: Table 2, entry 4
f1 (ppm)

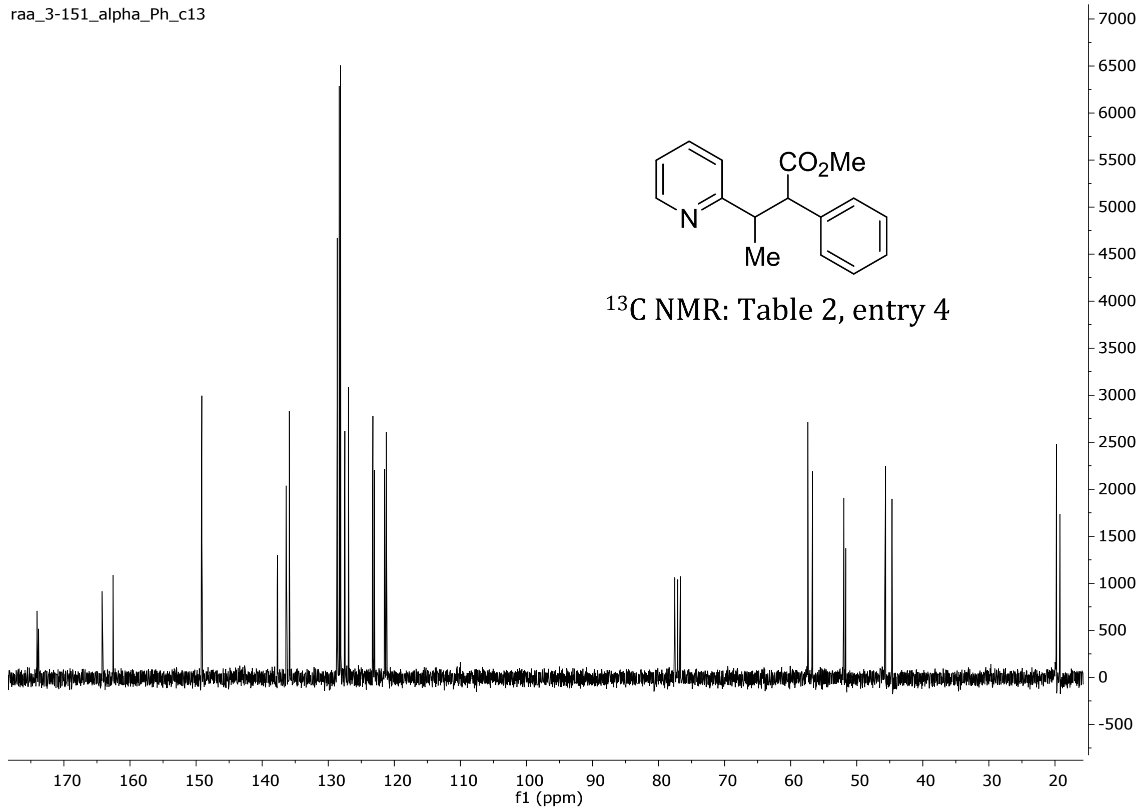
raa_3-151_alpha_Ph_c13
CO2Me
N
Me
13C NMR: Table 2, entry 4
f1 (ppm)

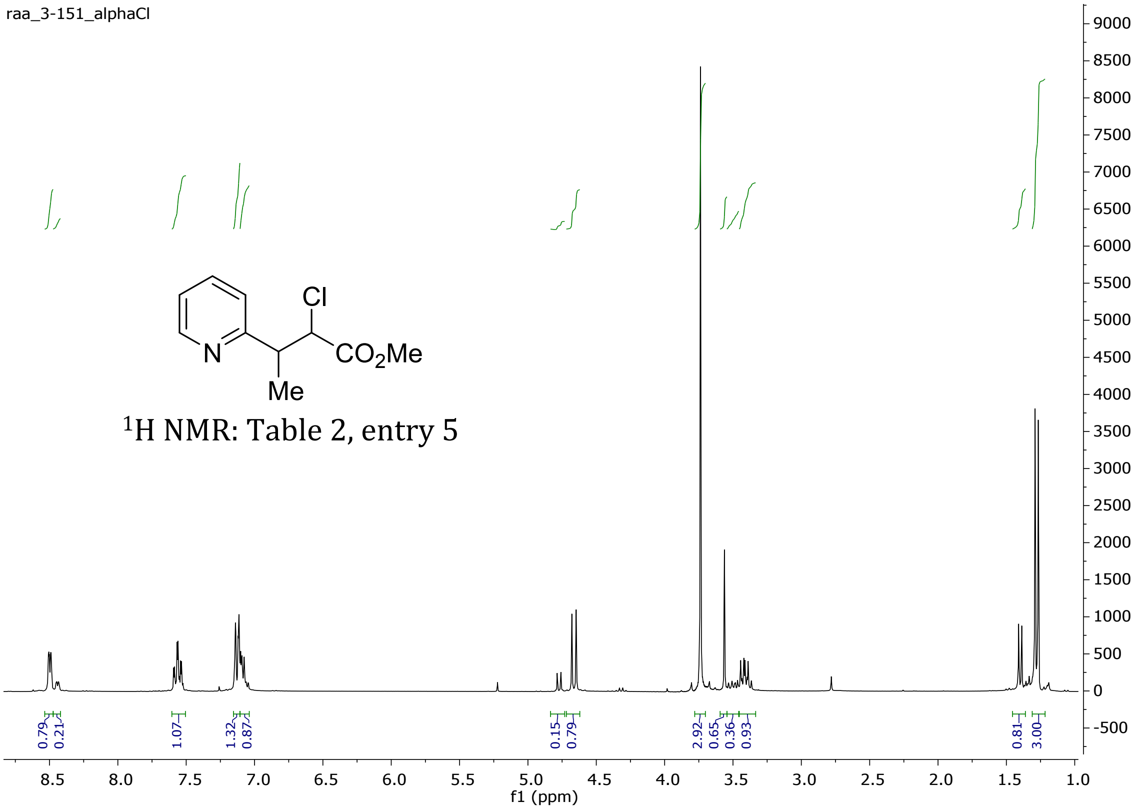
raa_3-151_alphaCl
Cl
N
CO2Me
Me
^{1}H NMR: Table 2, entry 5
f1 (ppm)

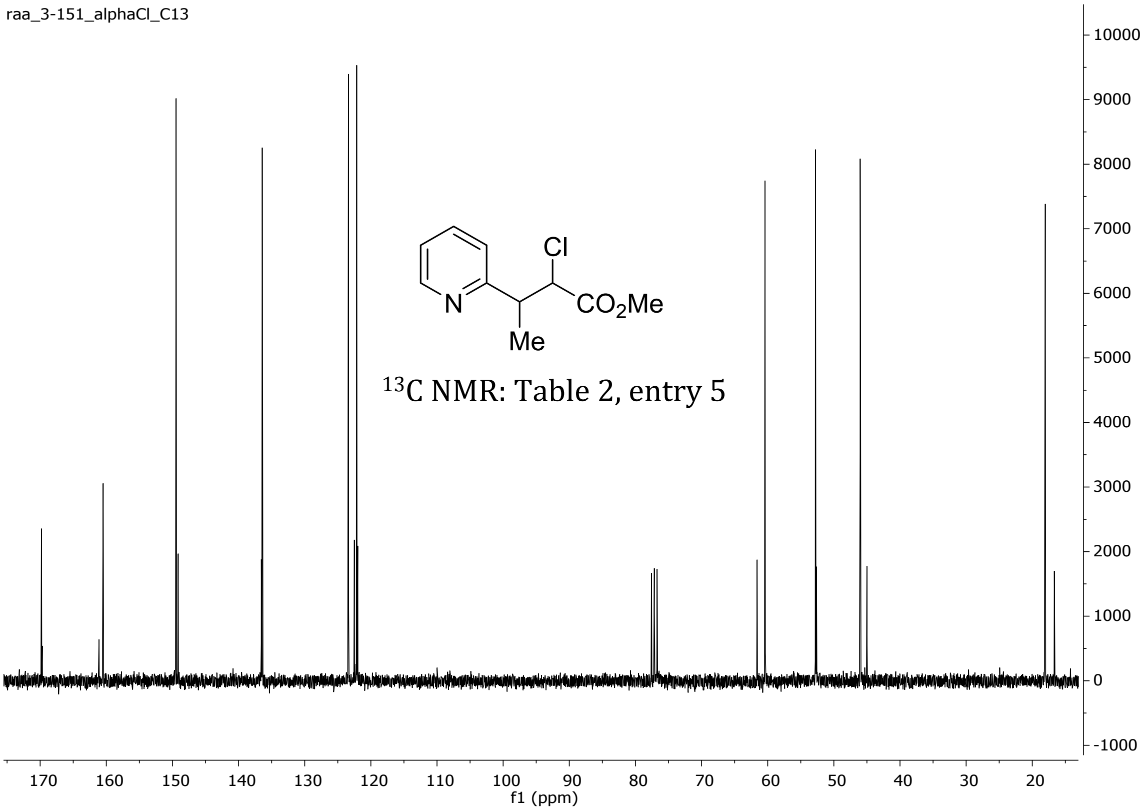
raa_3-151_alphaCl_C13
Cl
N
CO2Me
Me
^{13}C NMR: Table 2, entry 5
f1 (ppm)

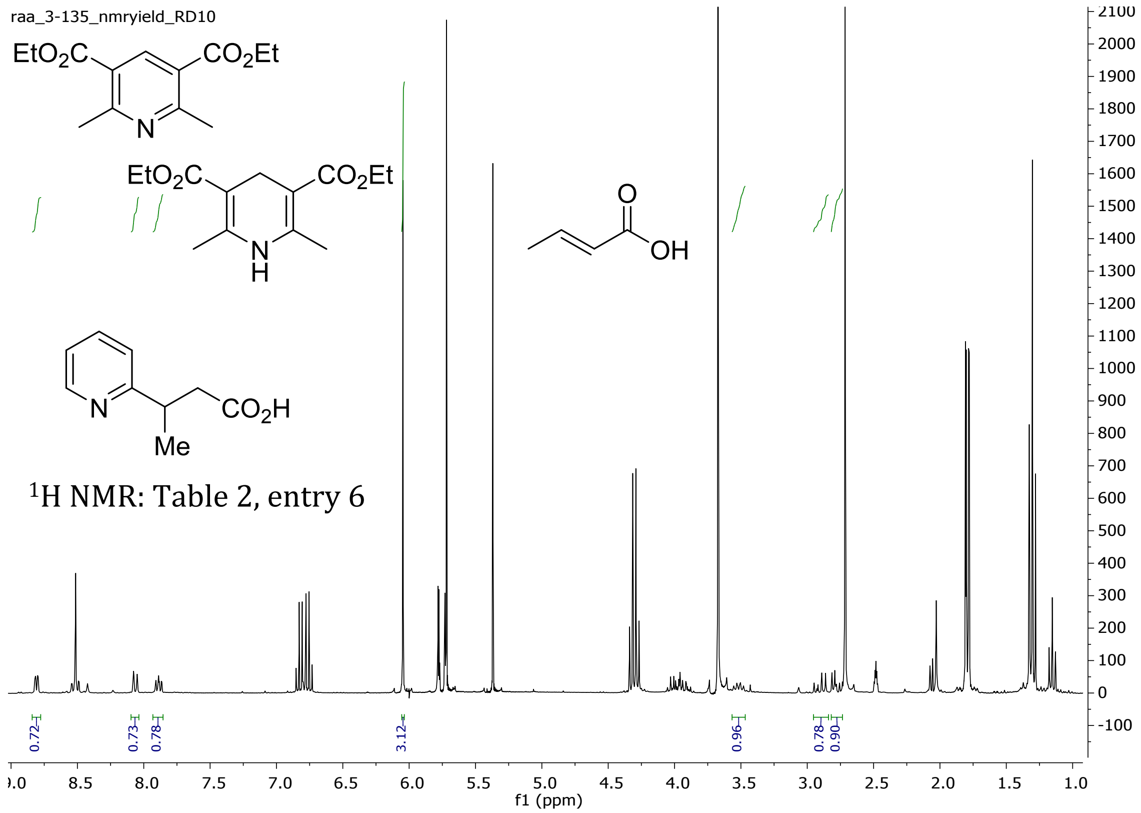
raa_3-135_nmryield_RD10
EtO2C
CO2Et
N
EtO2C
CO2Et
N
H
O
OH
N
CO2H
Me
1H NMR: Table 2, entry 6
0.72
0.73
0.78
3.12
0.96
0.78
0.90
f1 (ppm)

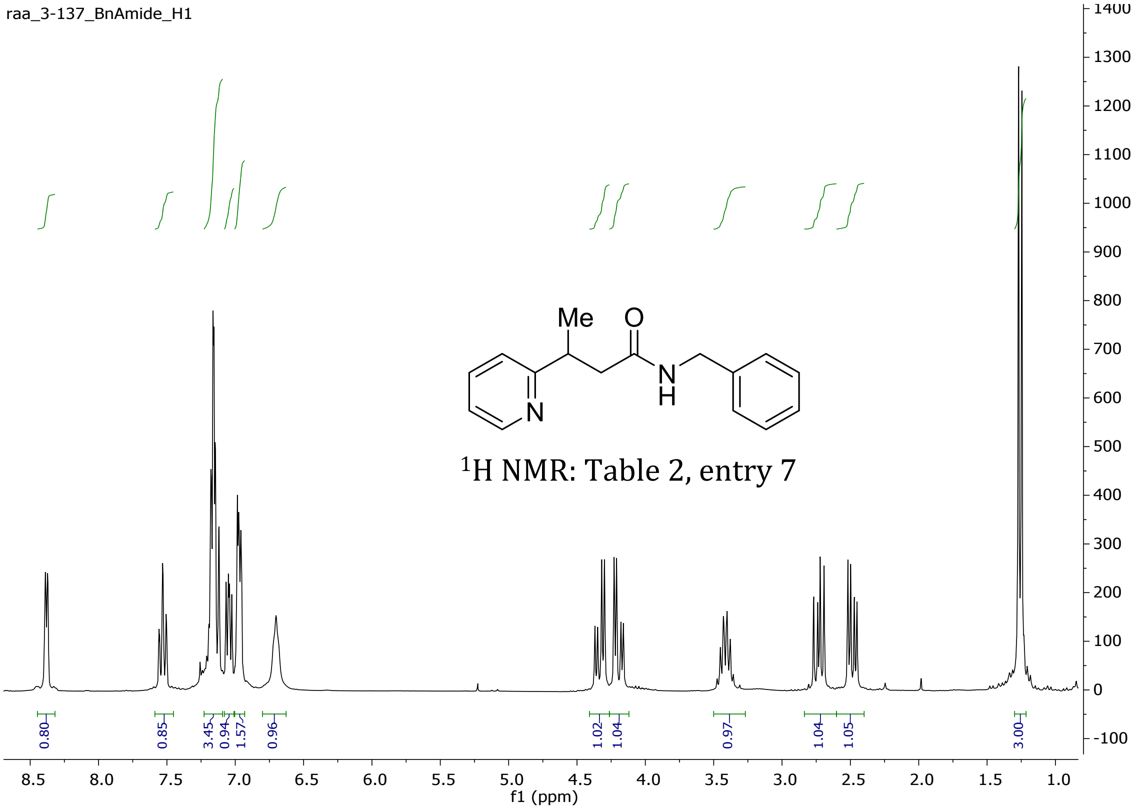
raa_3-137_BnAmide_H1
Me
O
N
H
N
¹H NMR: Table 2, entry 7
f1 (ppm)

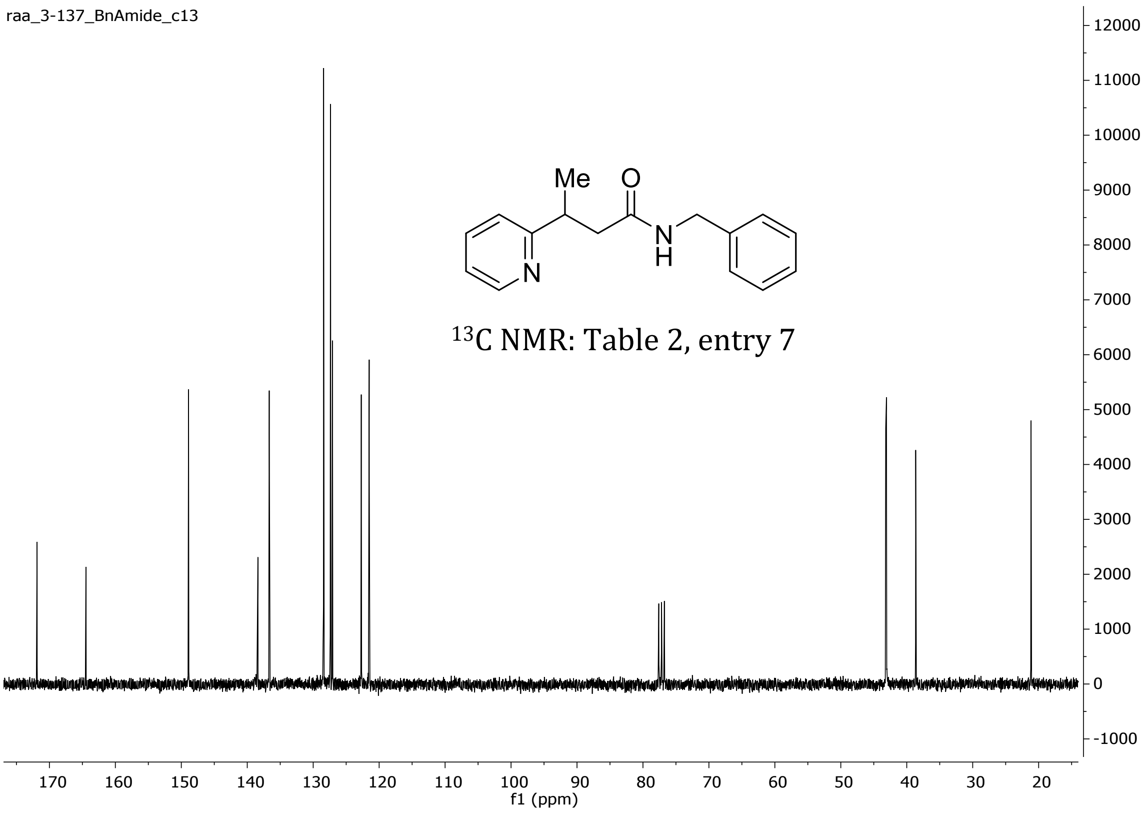
raa_3-137_BnAmide_c13
Me
O
N
H
N
¹³C NMR: Table 2, entry 7
f1 (ppm)

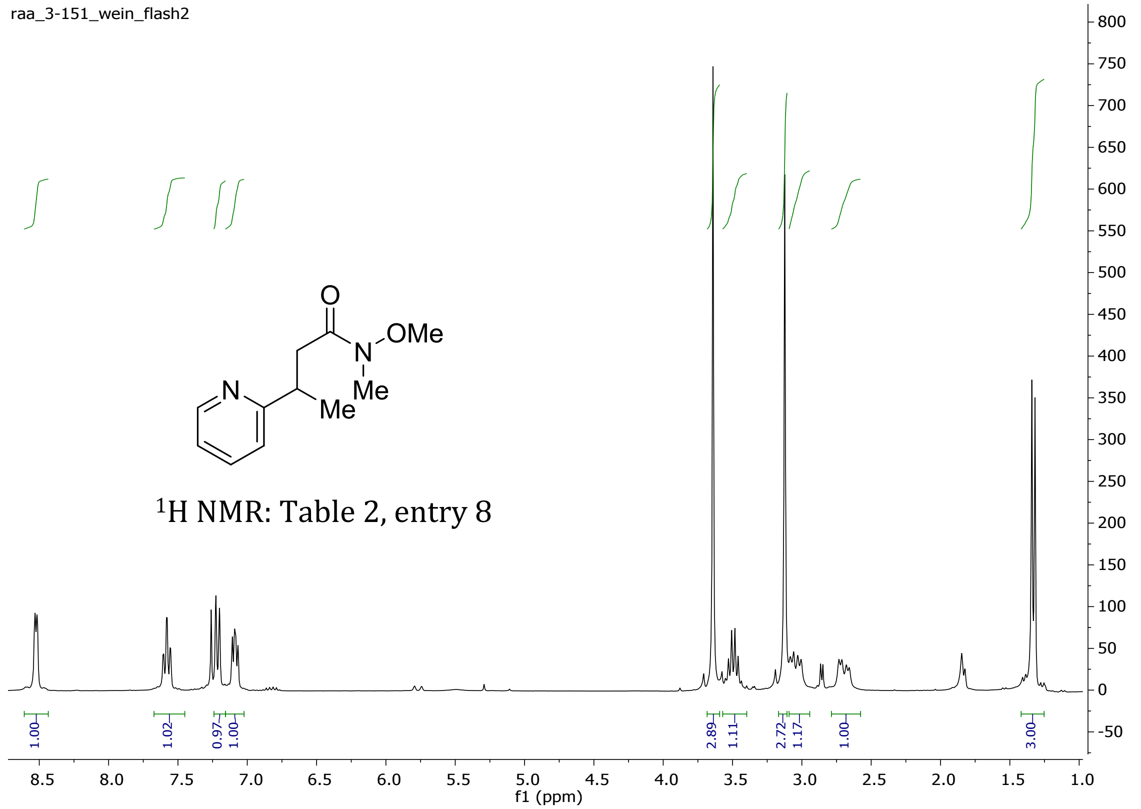
raa_3-151_wein_flash2
¹H NMR: Table 2, entry 8
f1 (ppm)

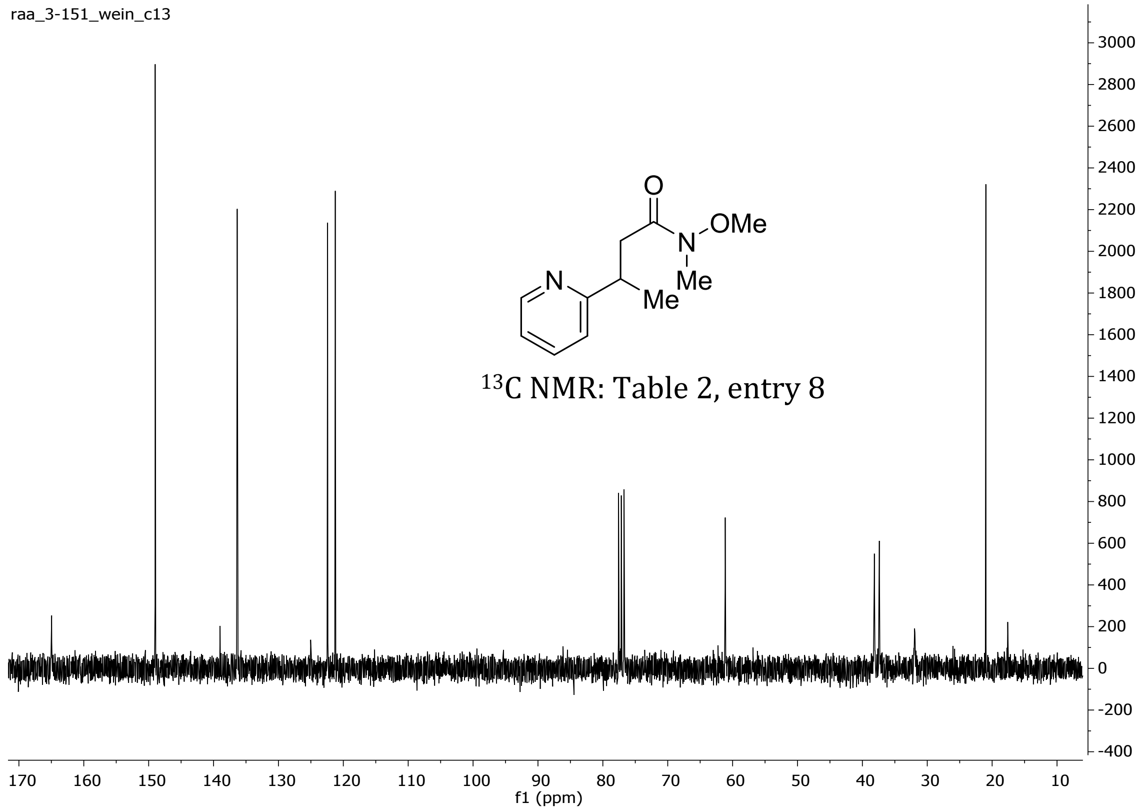
raa_3-151_wein_c13
¹³C NMR: Table 2, entry 8
f1 (ppm)

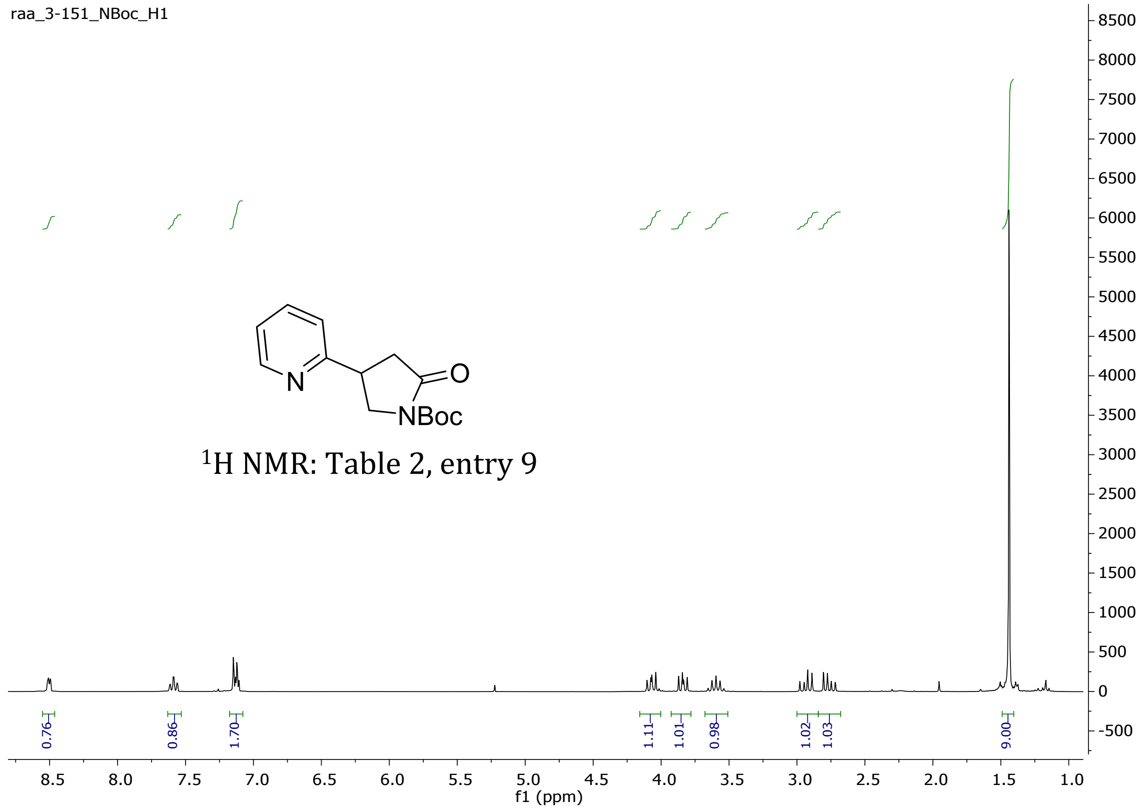
raa_3-151_NBoc_H1
N
O
NBoc
^{1}H NMR: Table 2, entry 9
f1 (ppm)

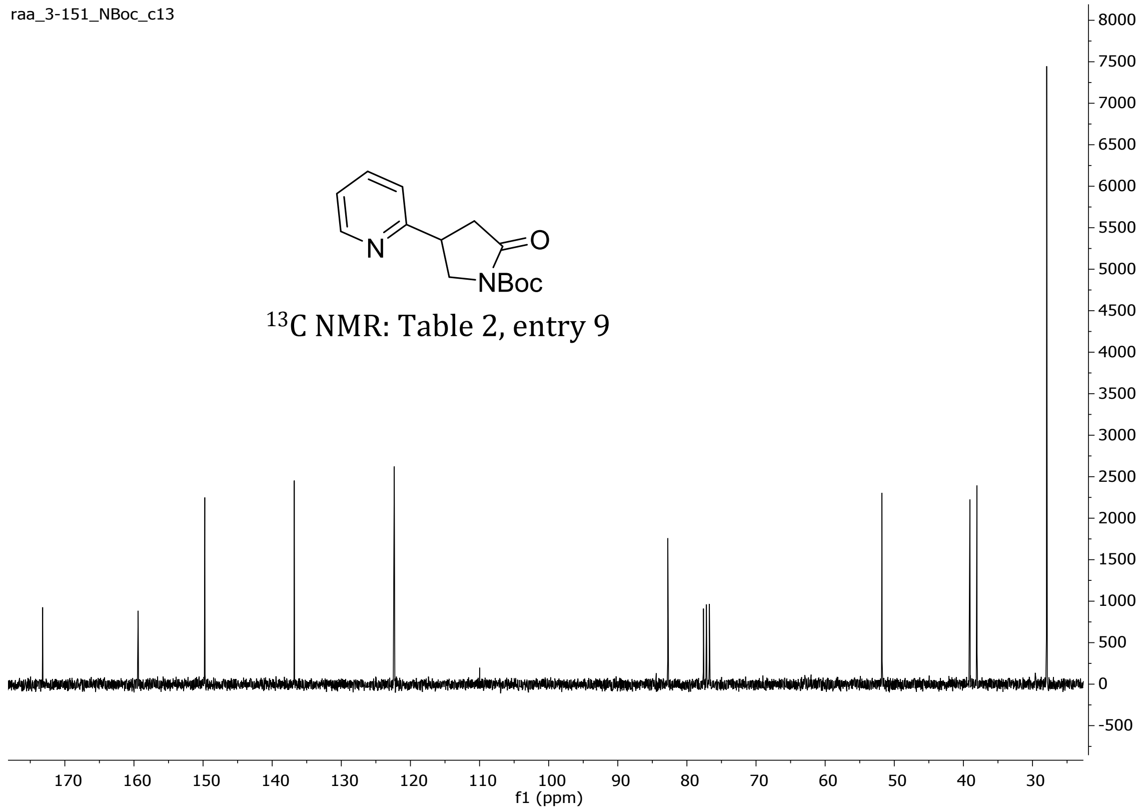
raa_3-151_NBoc_c13
N
O
NBoc
^{13}C NMR: Table 2, entry 9
f1 (ppm)

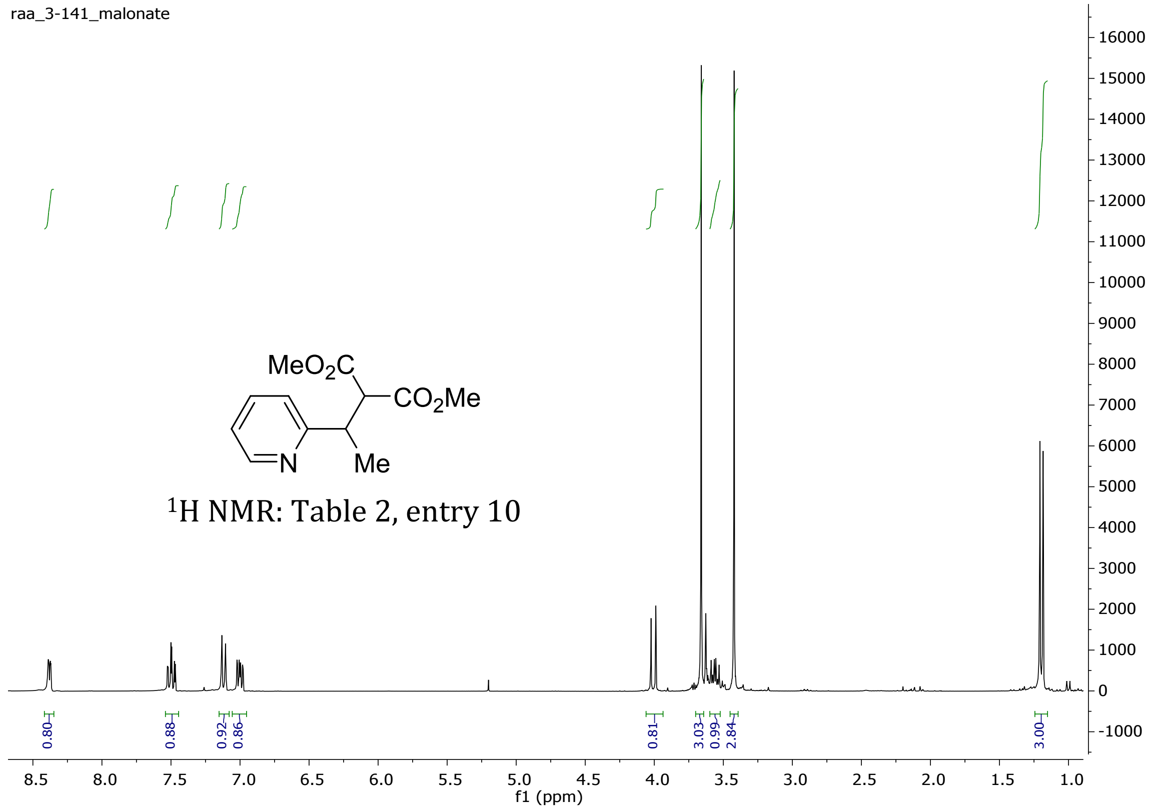
raa_3-141_malonate
MeO2C
CO2Me
N
Me
1H NMR: Table 2, entry 10
f1 (ppm)

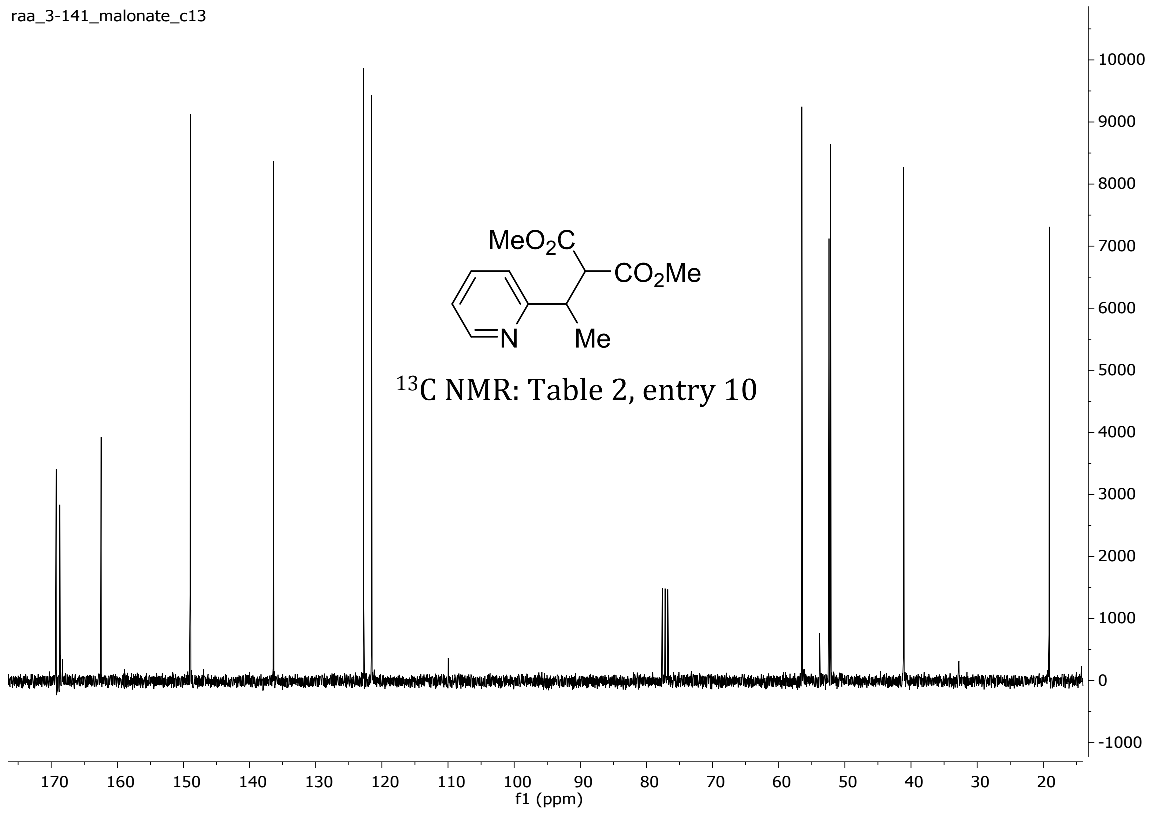
raa_3-141_malonate_c13
MeO2C
CO2Me
N
Me
13C NMR: Table 2, entry 10
f1 (ppm)

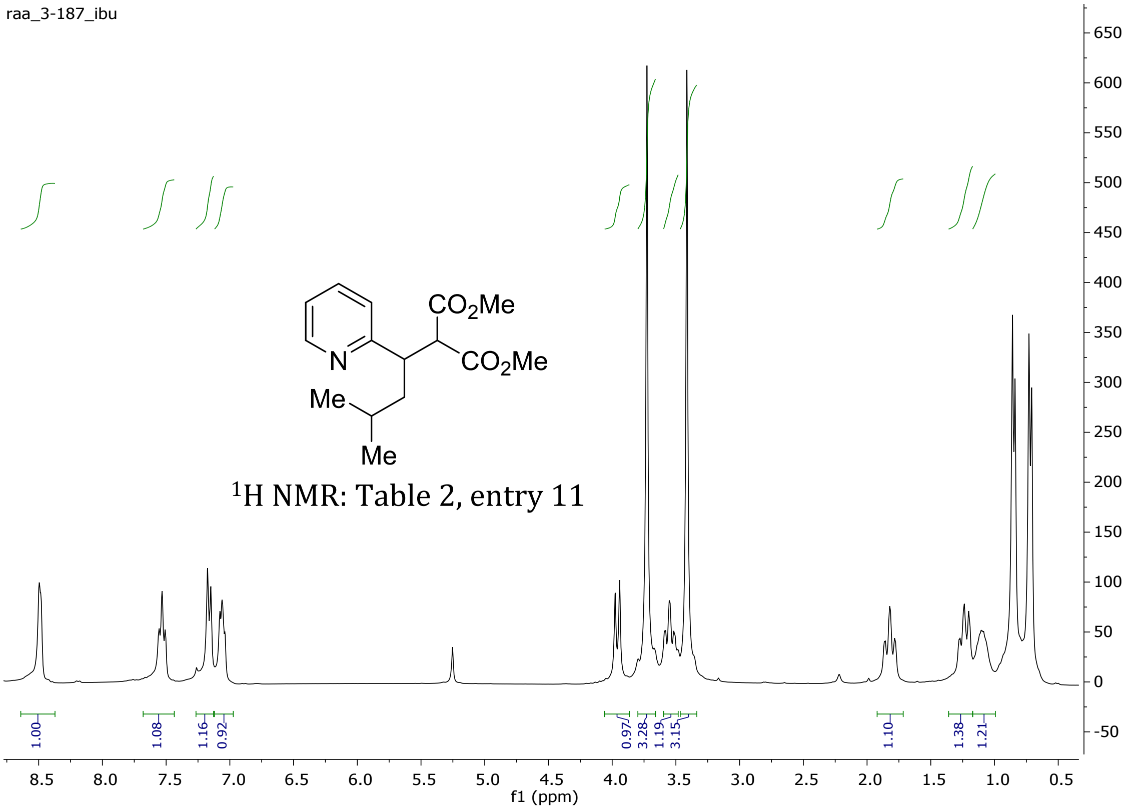
raa_3-187_ibu
CO2Me
N
CO2Me
Me
Me
1H NMR: Table 2, entry 11
1.00
1.08
1.16
0.92
0.97
3.28
1.19
3.15
1.10
1.38
1.21
f1 (ppm)

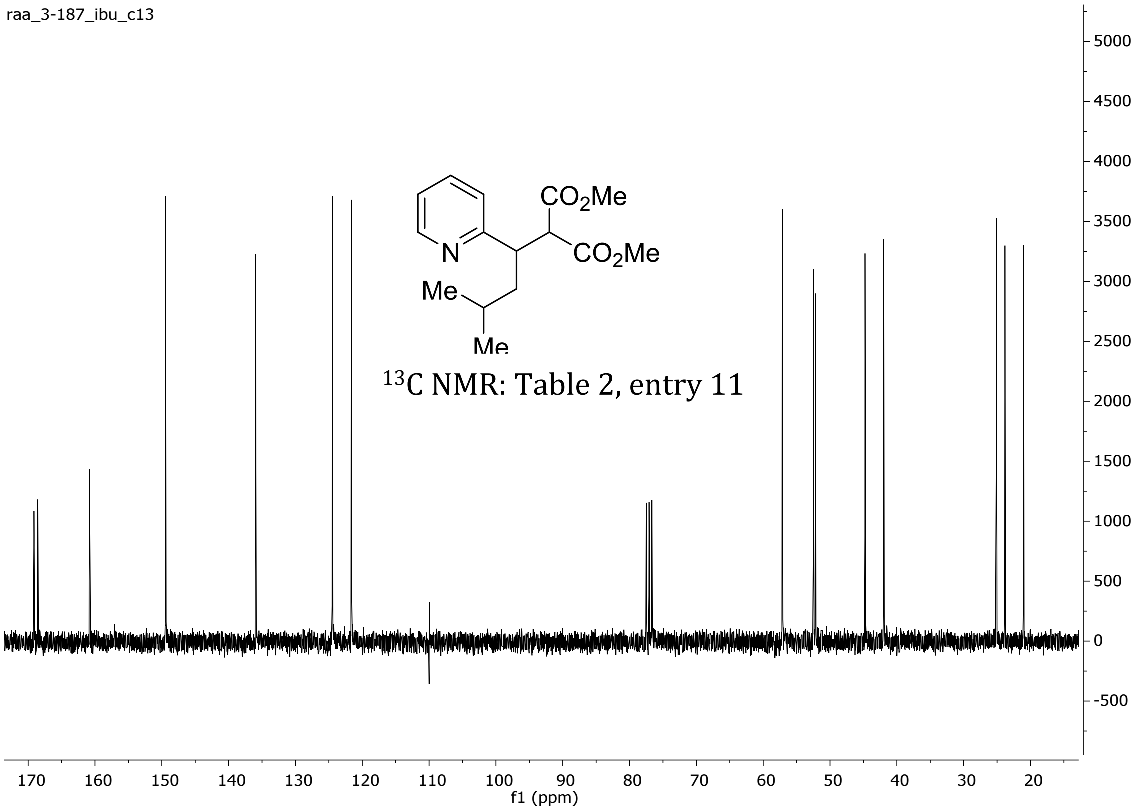
raa_3-187_ibu_c13
CO2Me
N
CO2Me
Me
Me
13C NMR: Table 2, entry 11
f1 (ppm)

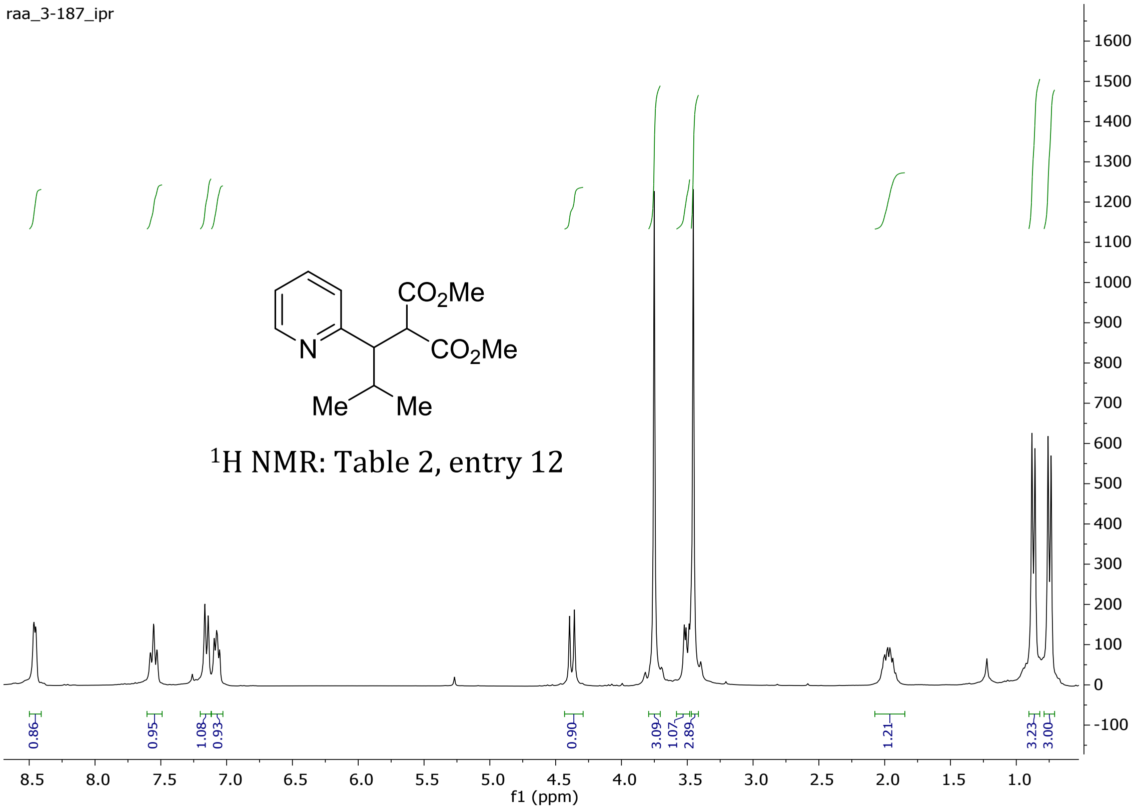
raa_3-187_ipr
CO2Me
CO2Me
N
Me
Me
1H NMR: Table 2, entry 12
f1 (ppm)

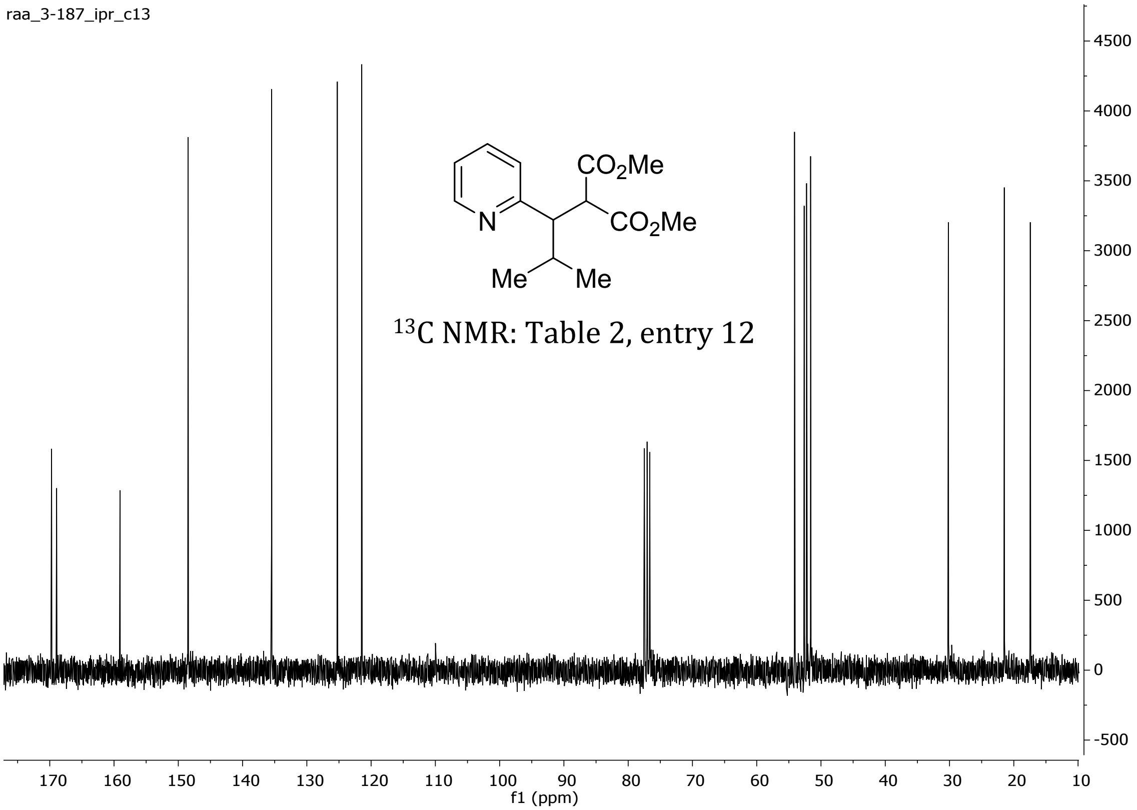
raa_3-187_ipr_c13
CO2Me
CO2Me
N
Me
Me
13C NMR: Table 2, entry 12
f1 (ppm)

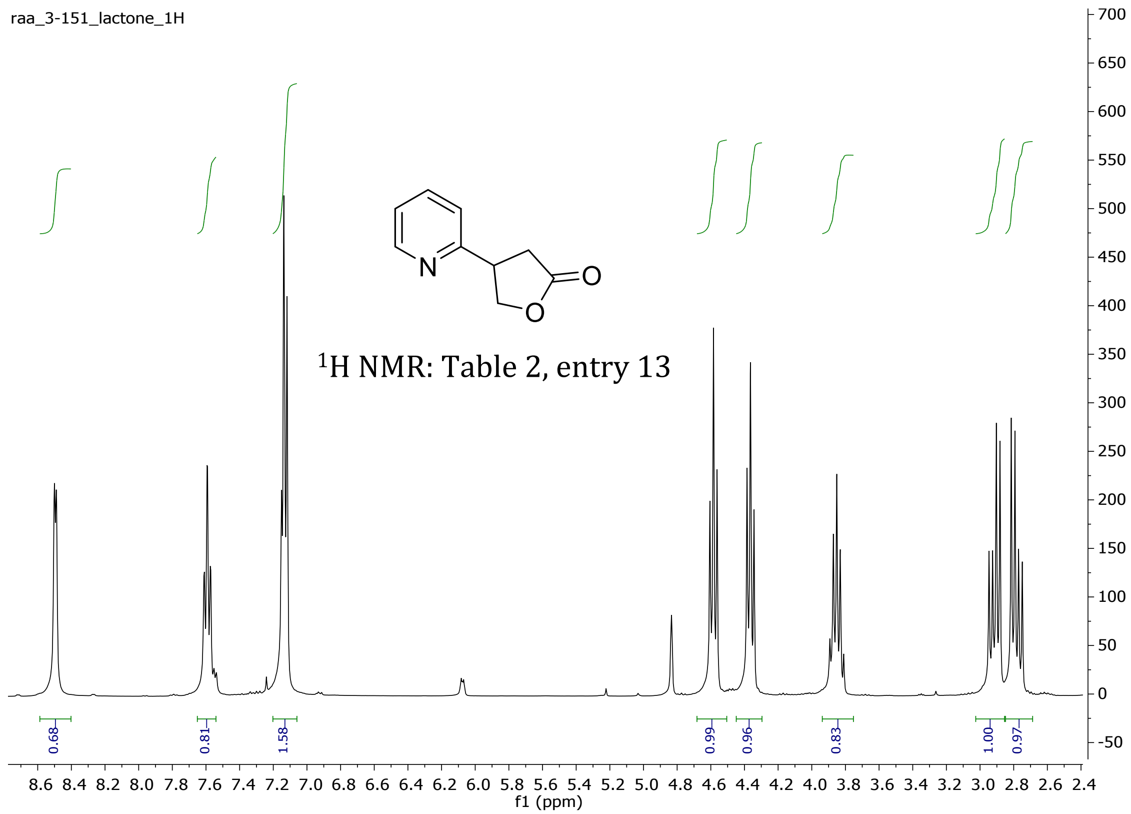
raa_3-151_lactone_1H
N
O
O
^{1}H NMR: Table 2, entry 13
f1 (ppm)

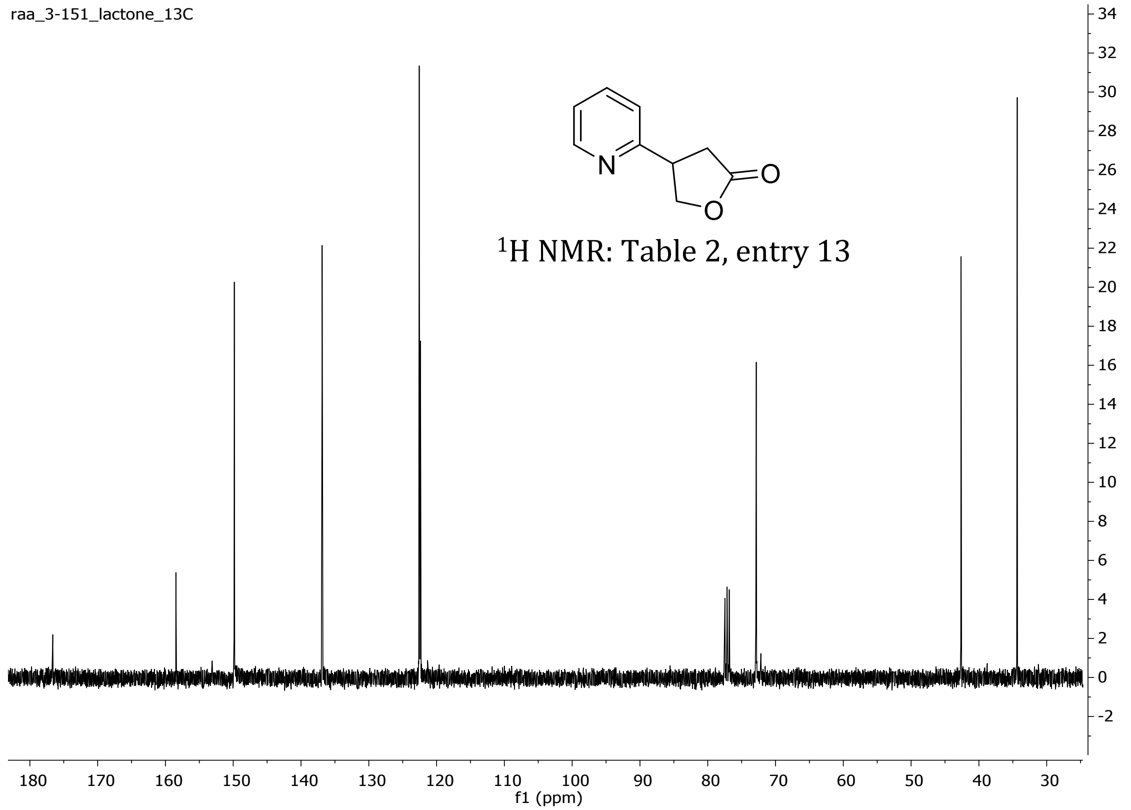
raa_3-151_lactone_13C
N
O
O
^{1}H NMR: Table 2, entry 13
f1 (ppm)

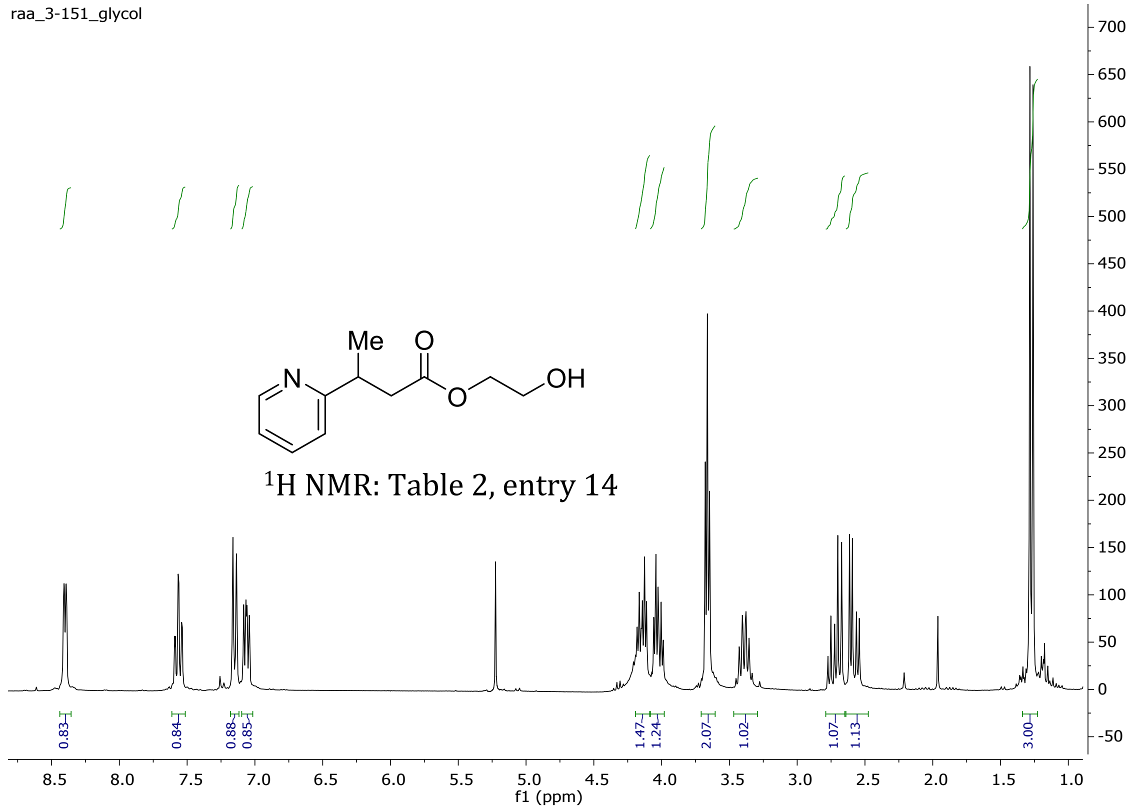

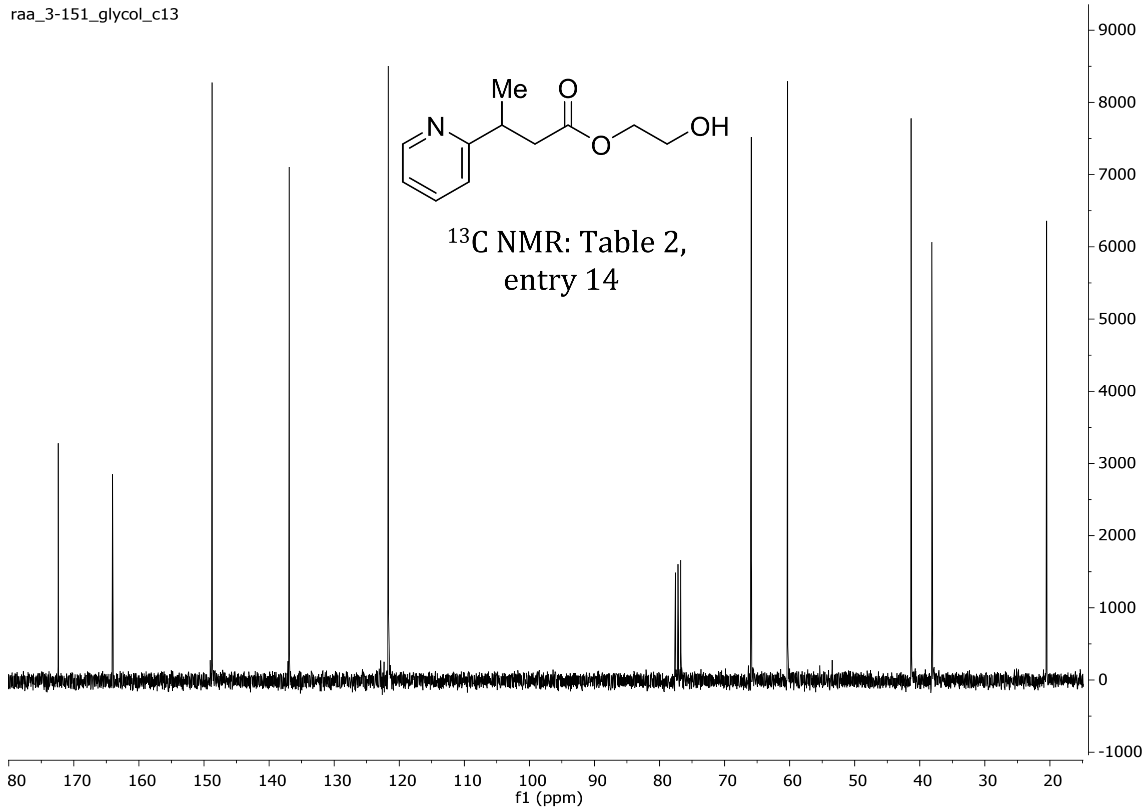

^{1}H NMR: Table 2, entry 14

^{13}C NMR: Table 2, entry 14

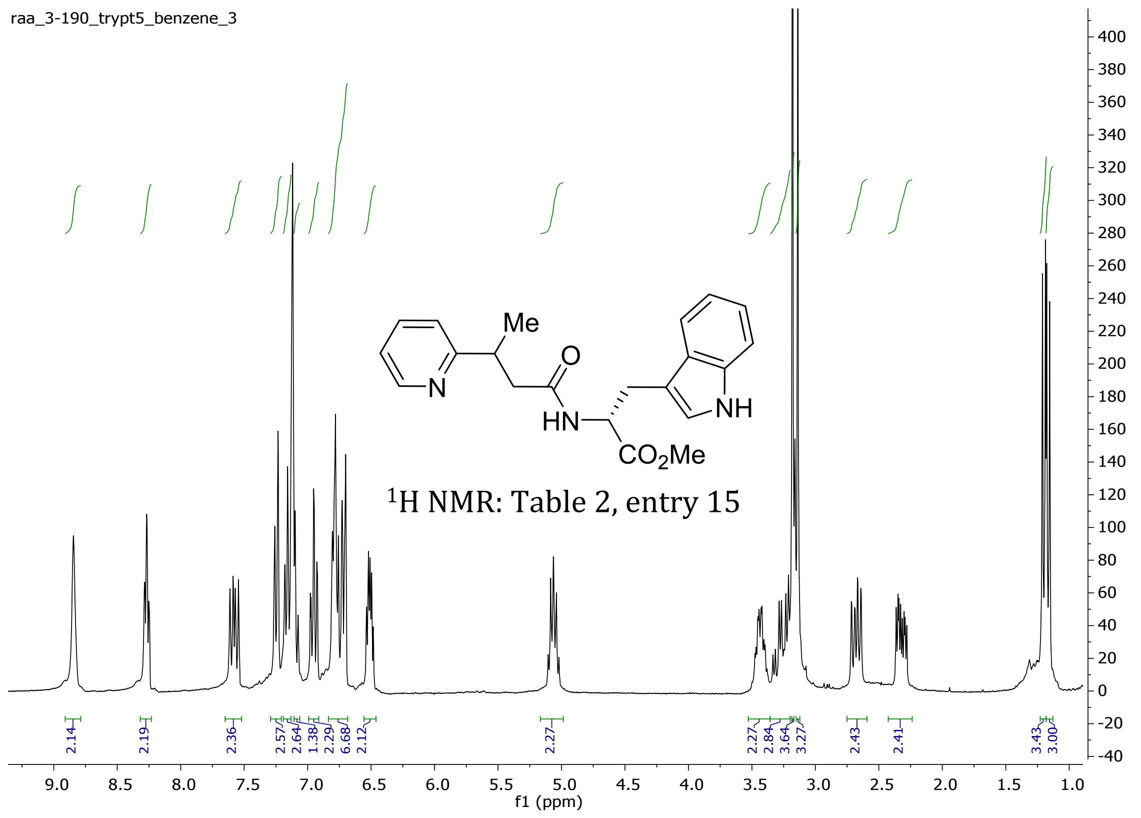
raa_3-190_trypt5_benzene_3
Me
O
N
HN
NH
CO2Me
1H NMR: Table 2, entry 15
f1 (ppm)

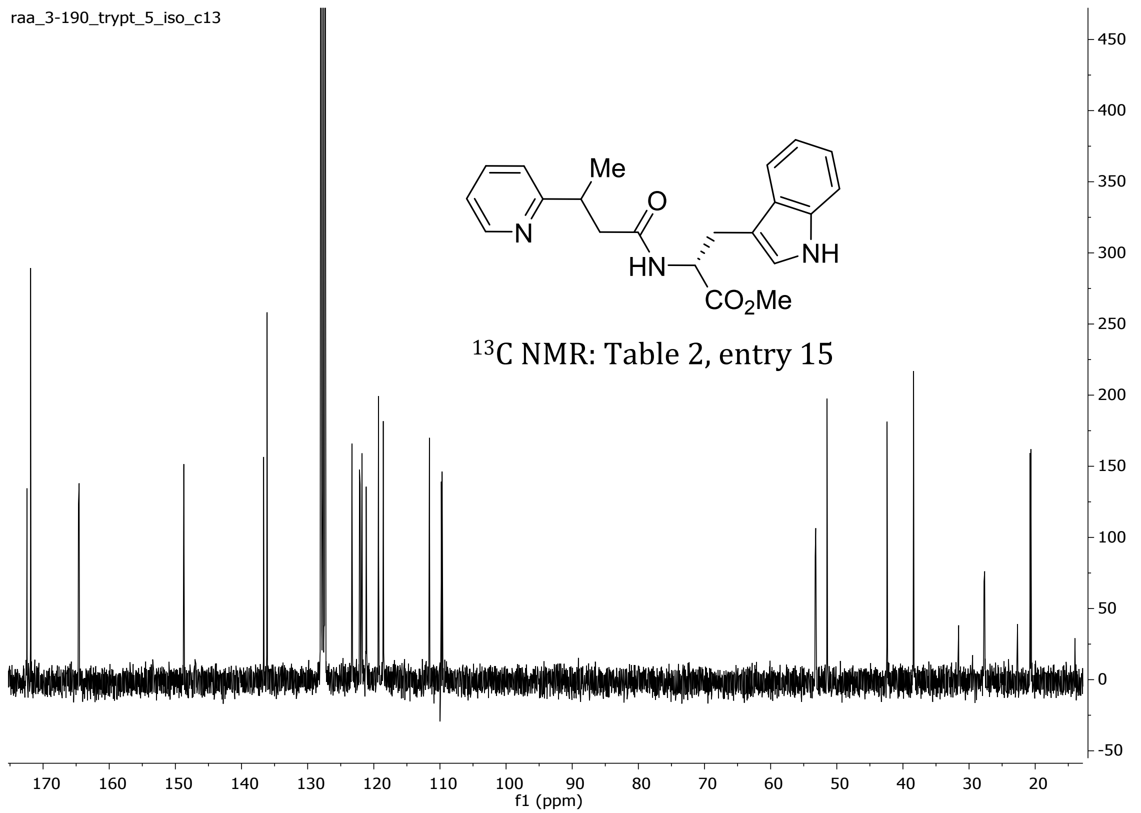
raa_3-190_trypt_5_iso_c13
Me
O
N
HN
NH
CO2Me
13C NMR: Table 2, entry 15
f1 (ppm)

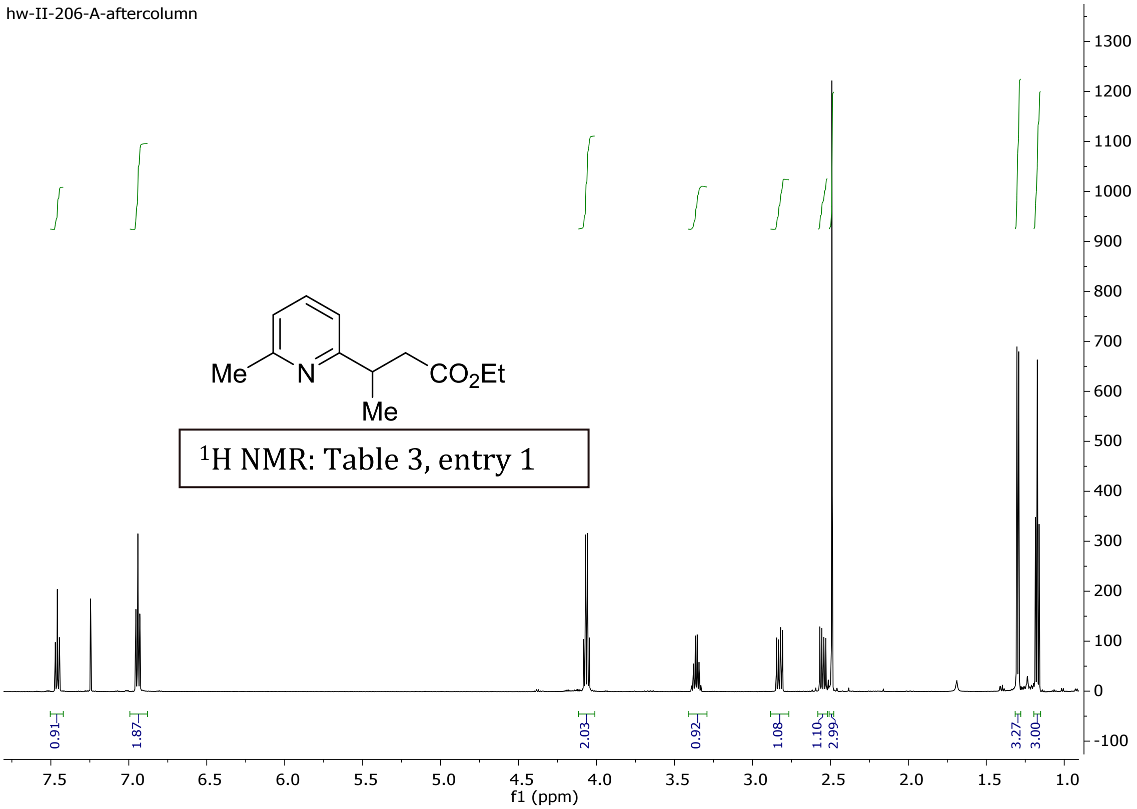
hw-II-206-A-aftercolumn
Me
N
CO2Et
Me
1H NMR: Table 3, entry 1
0.91
1.87
2.03
0.92
1.08
1.10
2.99
3.27
3.00
f1 (ppm)

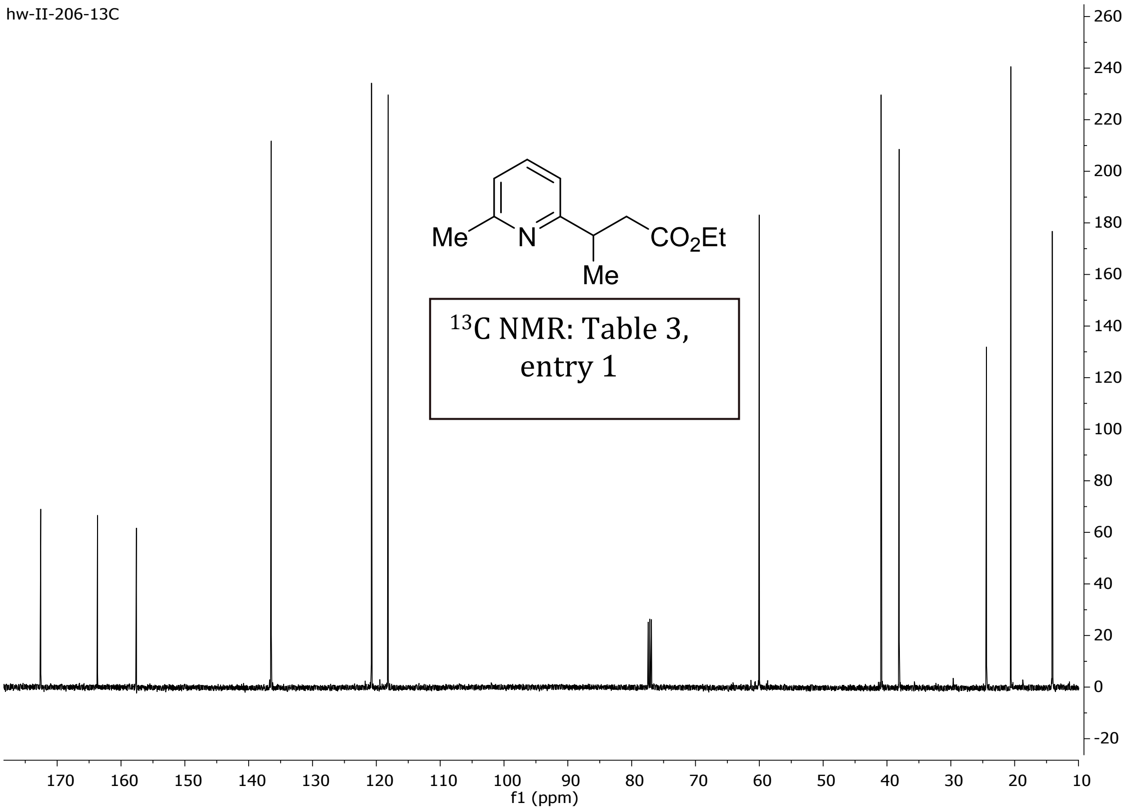
hw-II-206-13C
Me
N
CO2Et
Me
13C NMR: Table 3, entry 1
f1 (ppm)

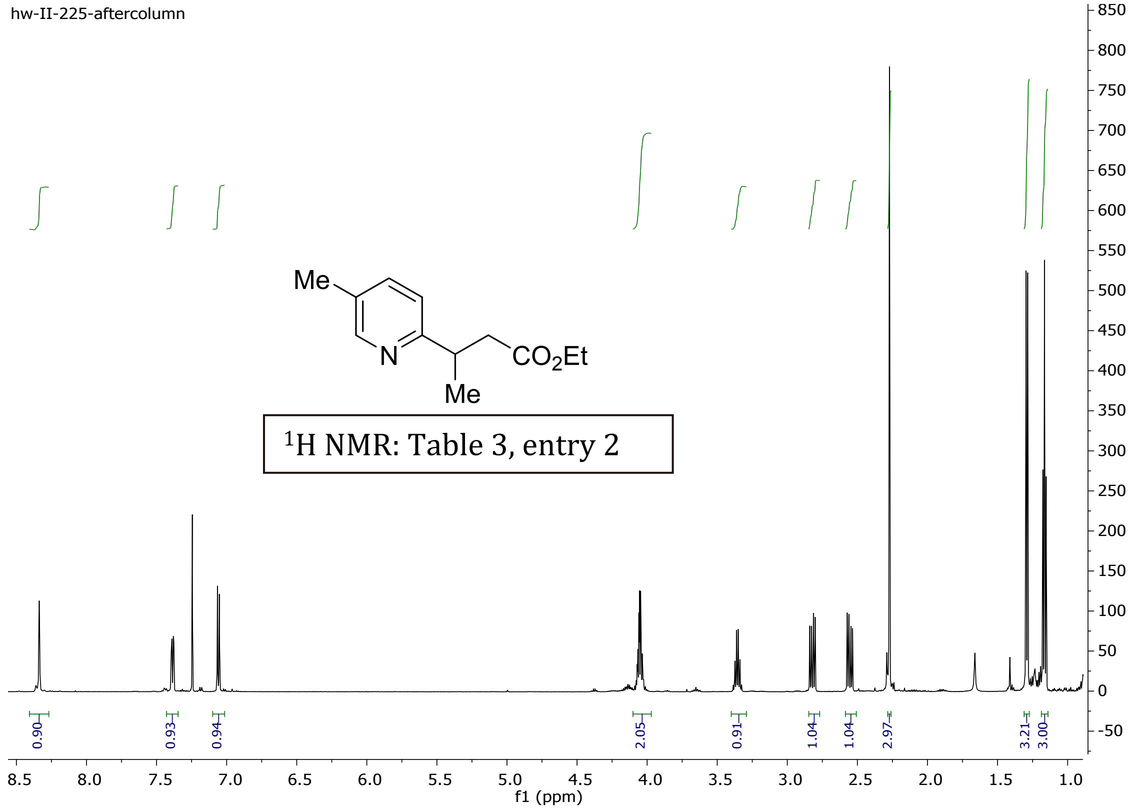
hw-II-225-aftercolumn
Me
N
CO2Et
Me
1H NMR: Table 3, entry 2
f1 (ppm)

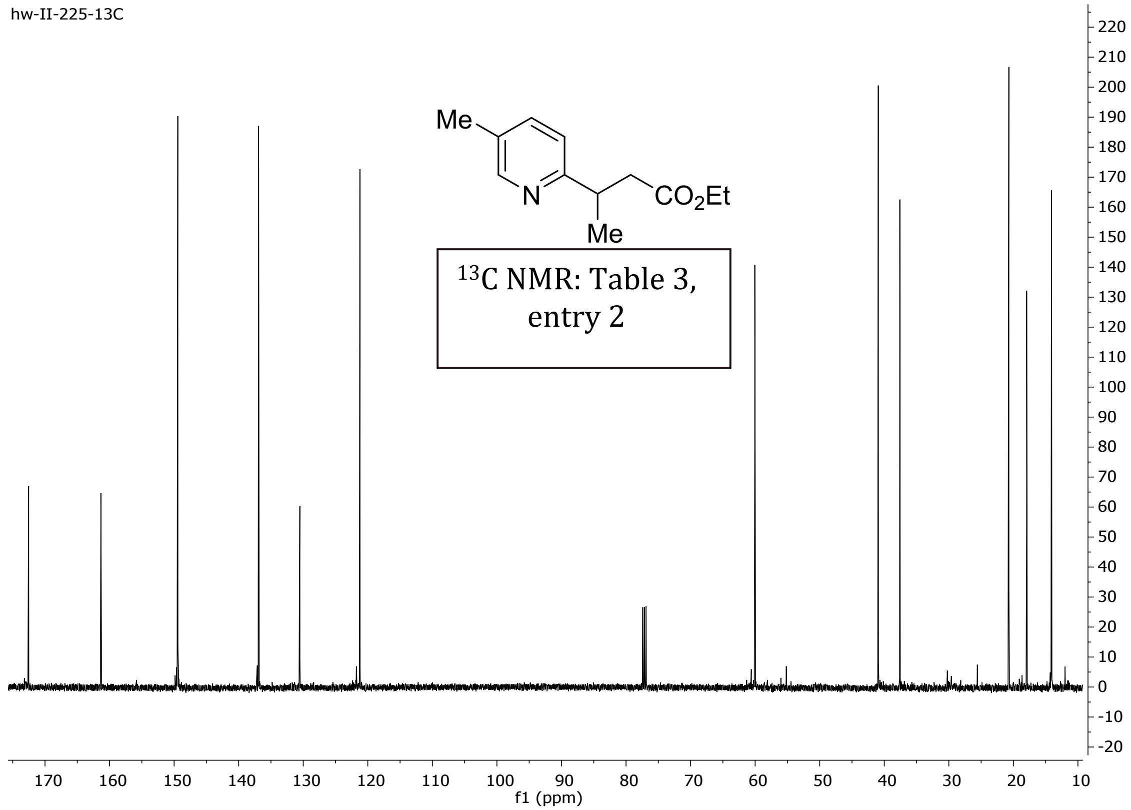
hw-II-225-13C
Me
N
CO2Et
Me
13C NMR: Table 3, entry 2
f1 (ppm)

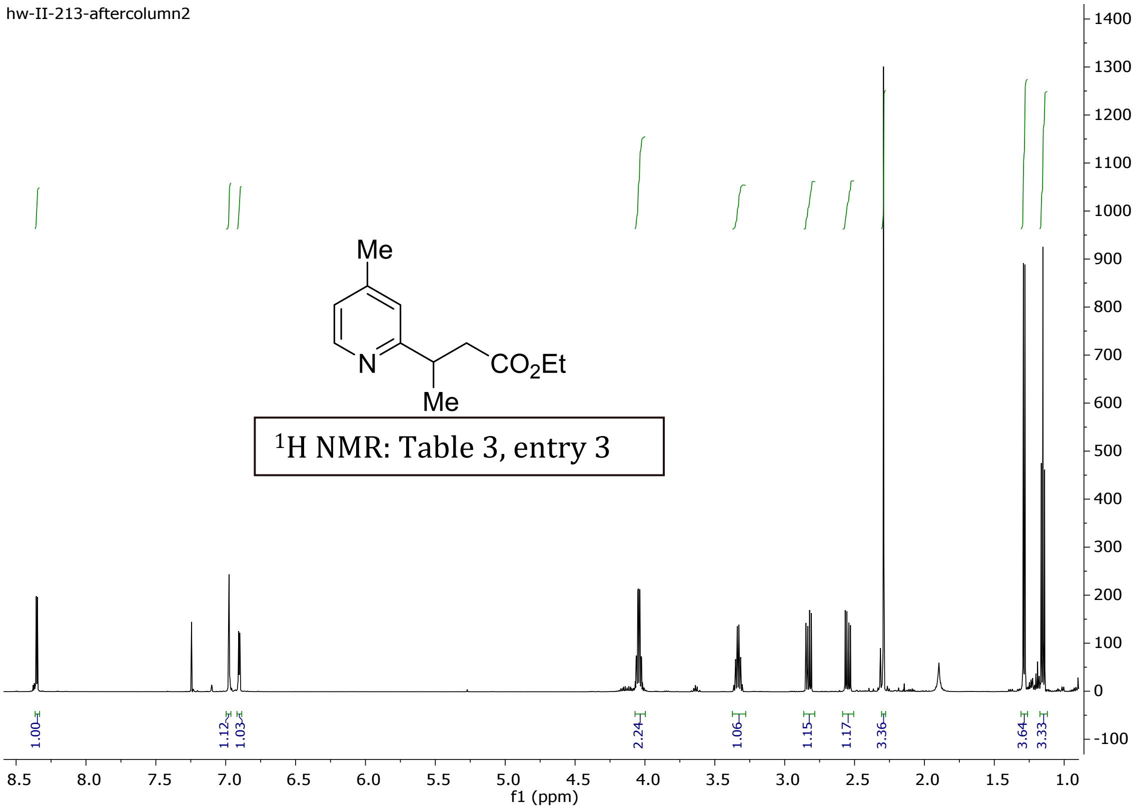

hw-II-213-aftercolumn2
Me
N
CO2Et
Me
1H NMR: Table 3, entry 3
1.00
1.12
1.03
2.24
1.06
1.15
1.17
3.36
3.64
3.33
f1 (ppm)

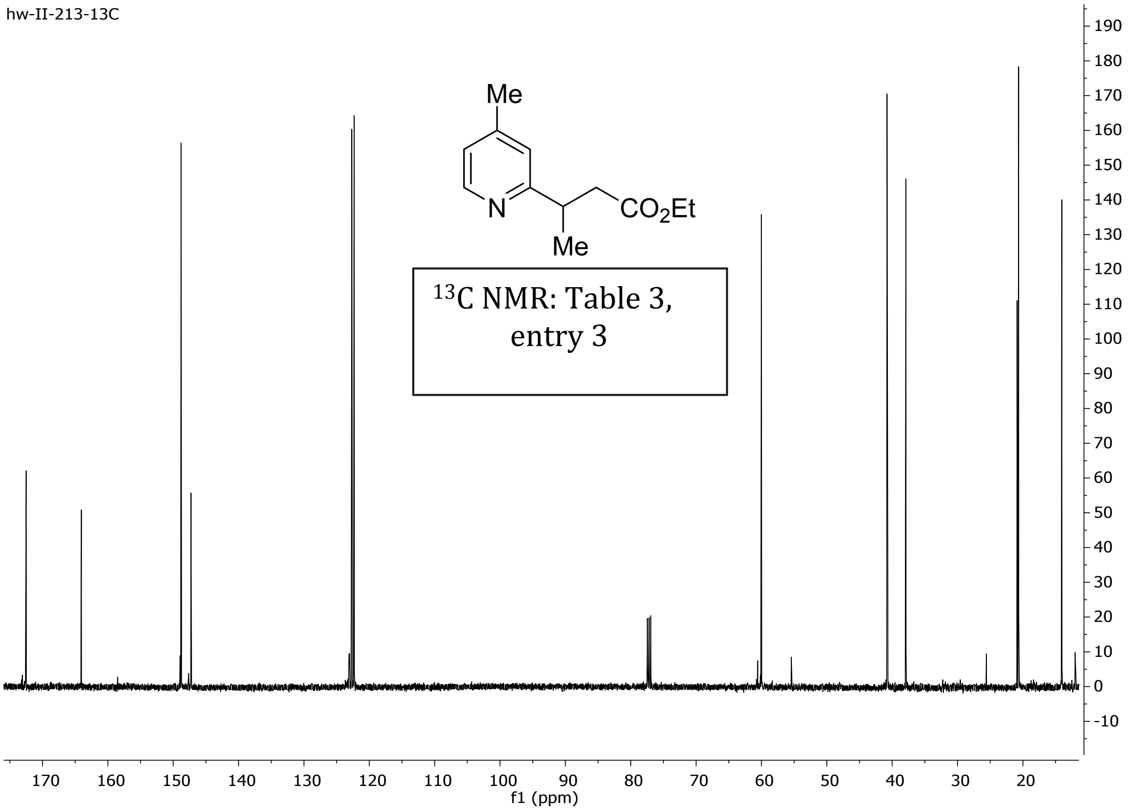

hw-II-213-13C
Me
N
CO2Et
Me
13C NMR: Table 3, entry 3
f1 (ppm)

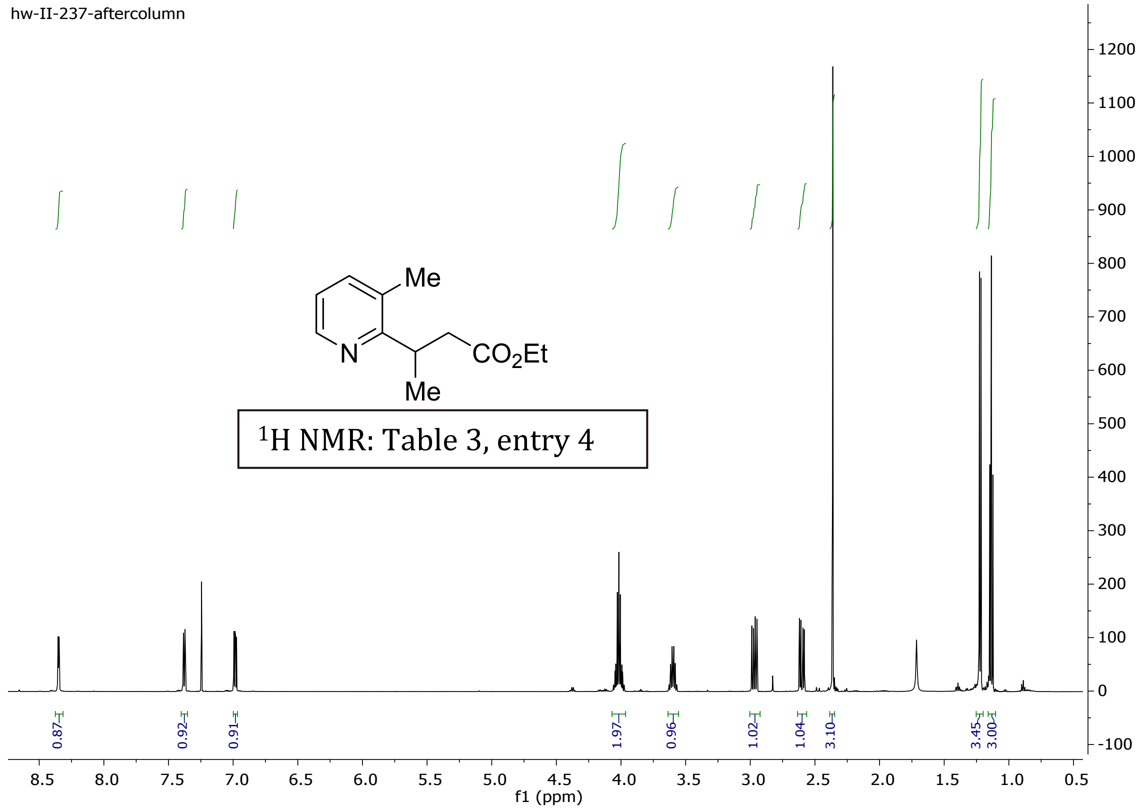
hw-II-237-aftercolumn
Me
N
CO2Et
Me
1H NMR: Table 3, entry 4
0.87
0.92
0.91
1.97
0.96
1.02
1.04
3.10
3.45
3.00
f1 (ppm)

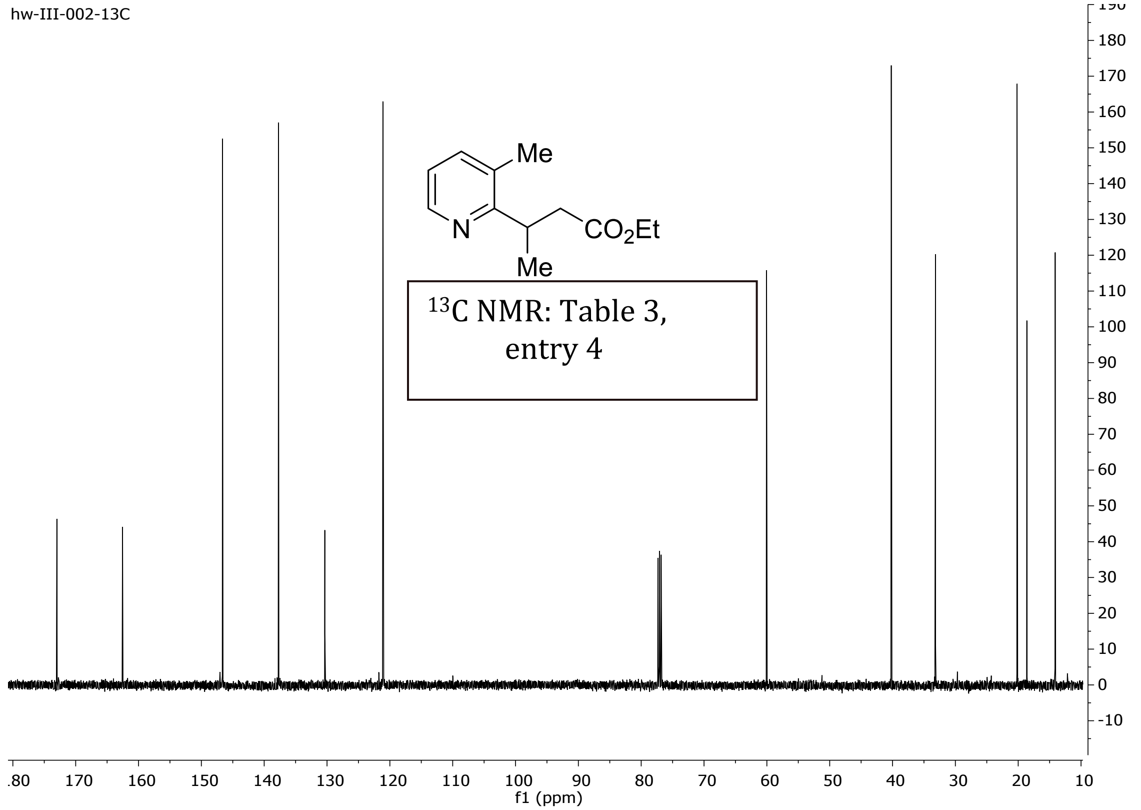
hw-III-002-13C
Me
N
CO2Et
Me
13C NMR: Table 3, entry 4
f1 (ppm)

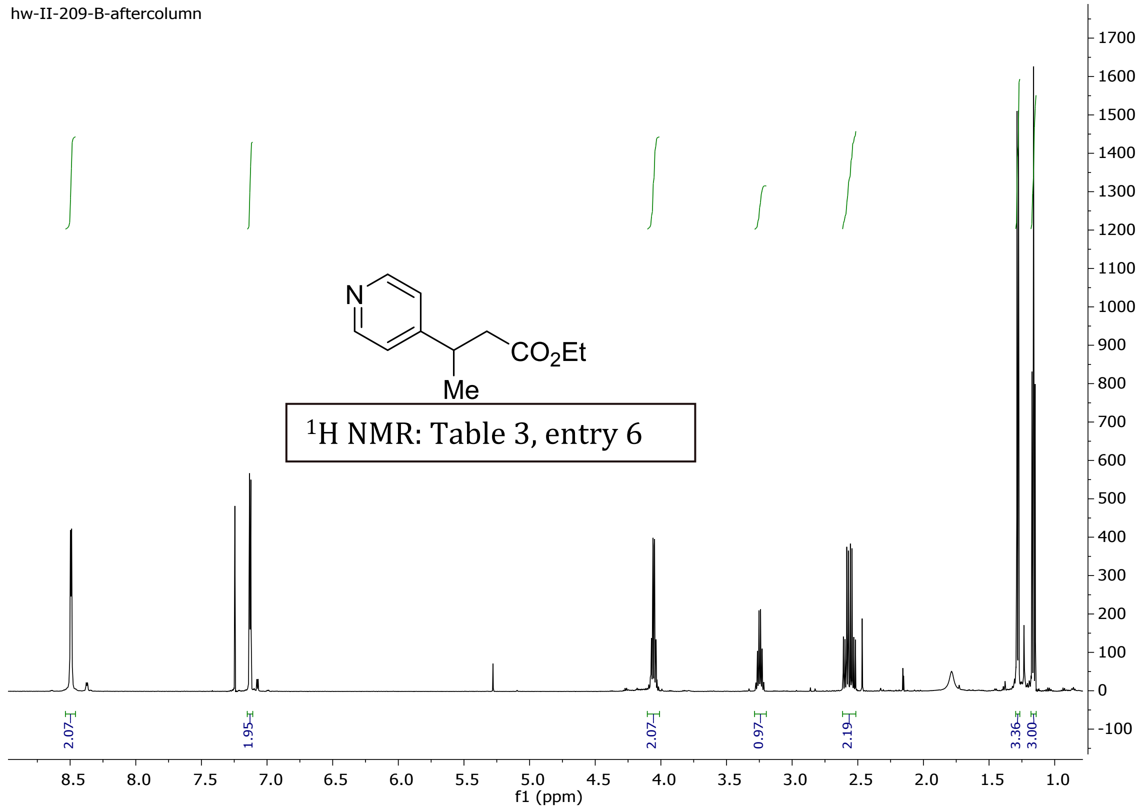
hw-II-209-B-aftercolumn
N
CO2Et
Me
1H NMR: Table 3, entry 6
2.07
1.95
2.07
0.97
2.19
3.36
3.00
f1 (ppm)

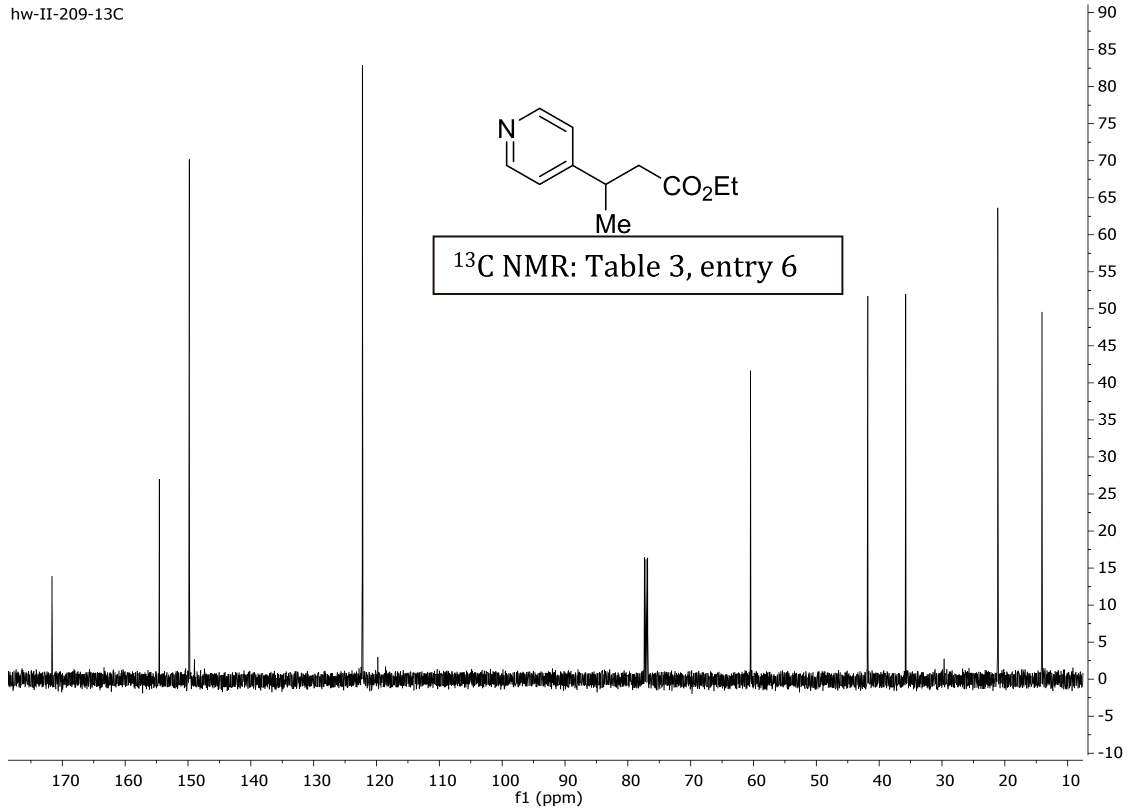
hw-II-209-13C
N
CO2Et
Me
13C NMR: Table 3, entry 6
f1 (ppm)

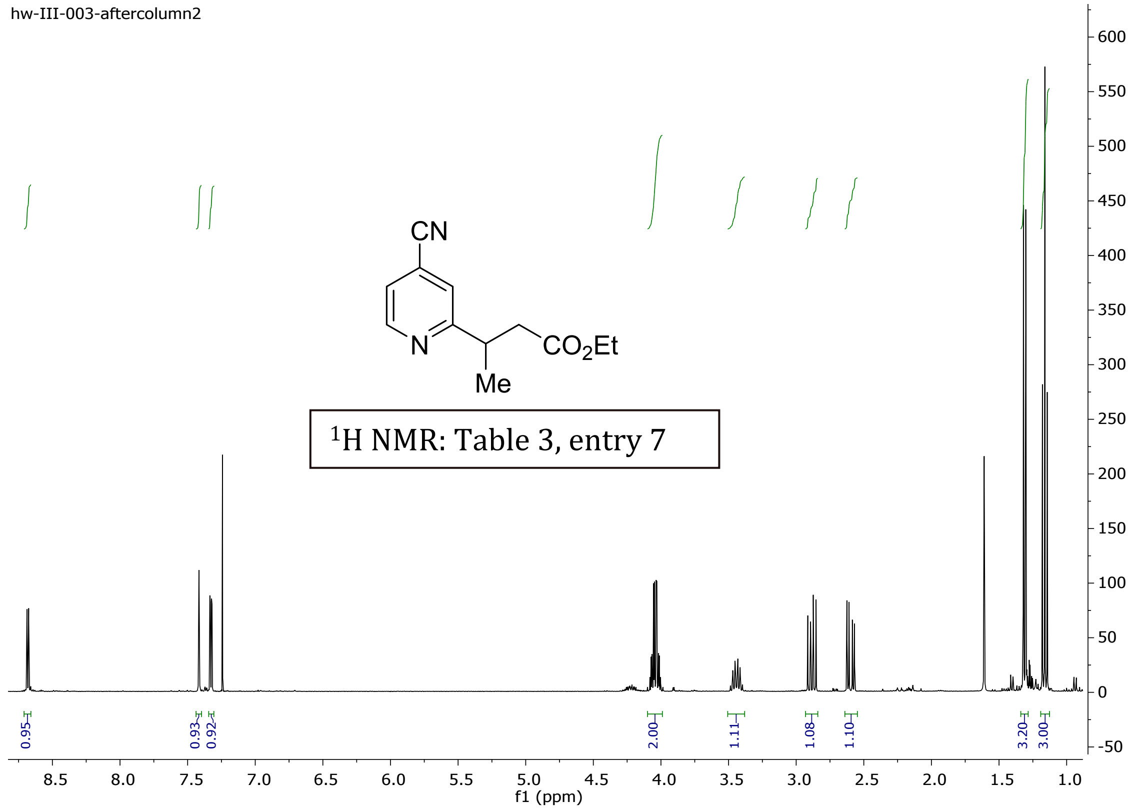

hw-III-003-aftercolumn2
CN
N
CO2Et
Me
1H NMR: Table 3, entry 7
f1 (ppm)

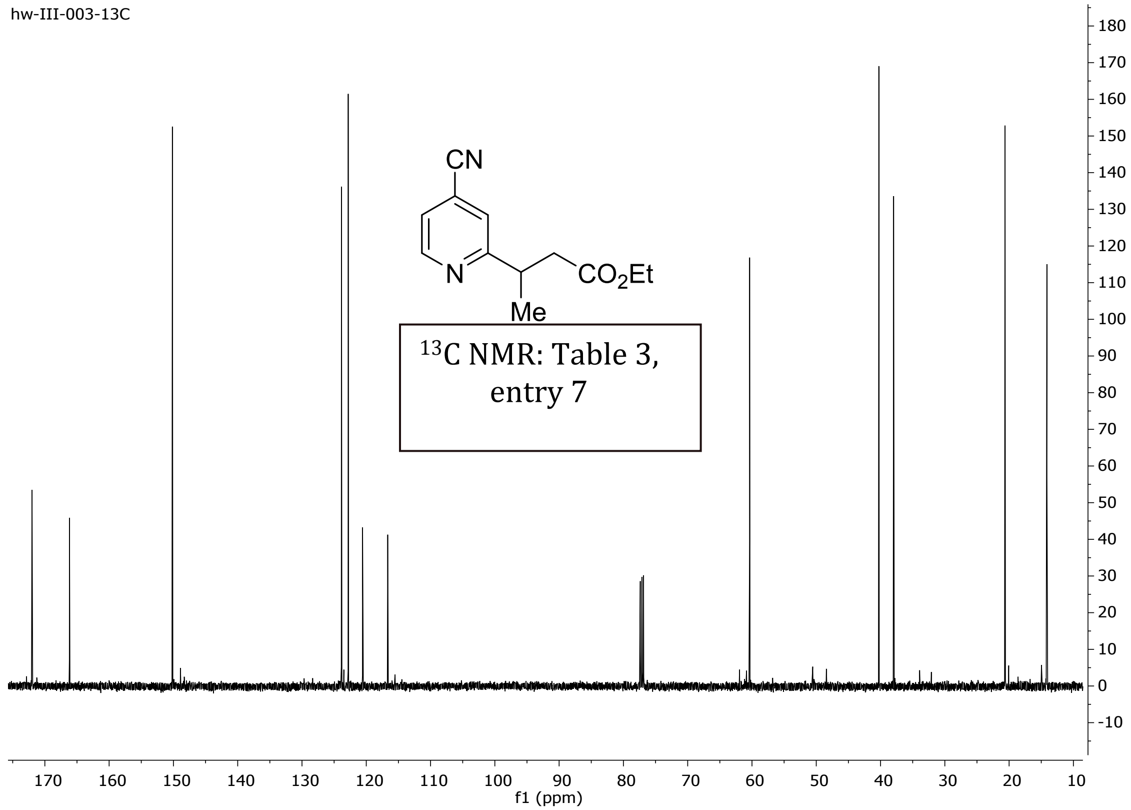

hw-III-003-13C
CN
N
CO2Et
Me
13C NMR: Table 3, entry 7
f1 (ppm)

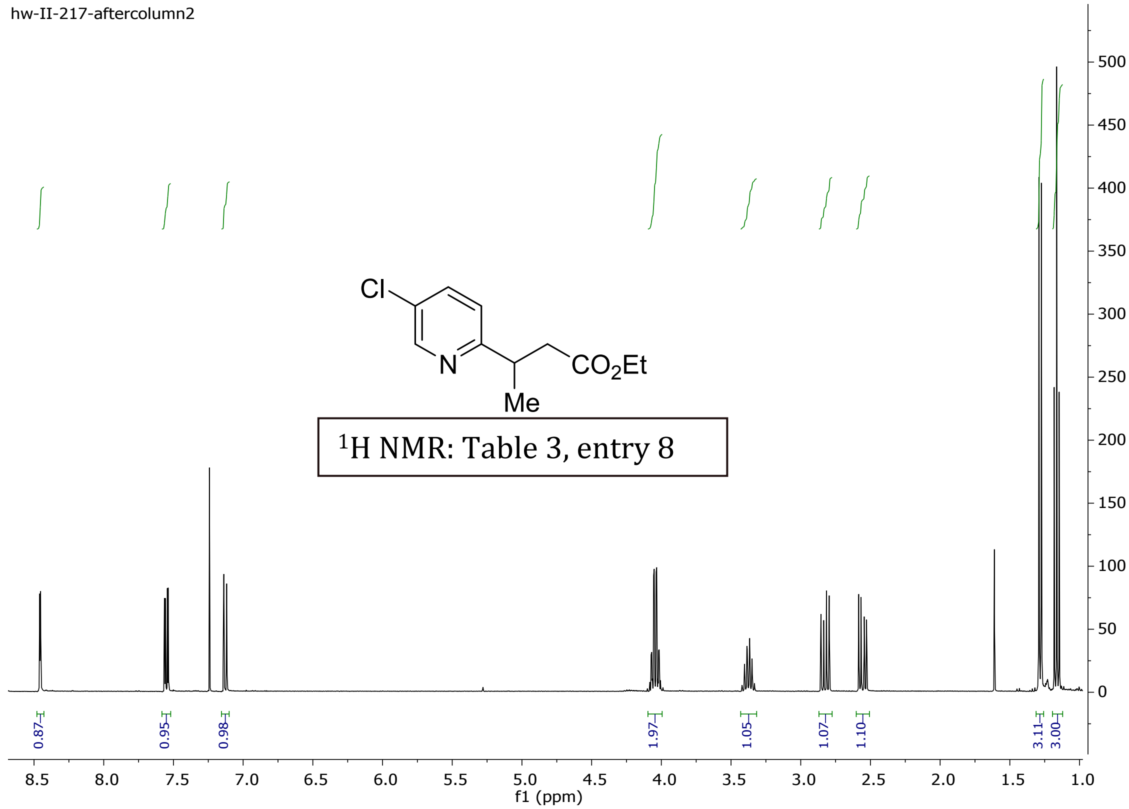
hw-II-217-aftercolumn2
Cl
N
CO2Et
Me
1H NMR: Table 3, entry 8
0.87
0.95
0.98
1.97
1.05
1.07
1.10
3.11
3.00
f1 (ppm)

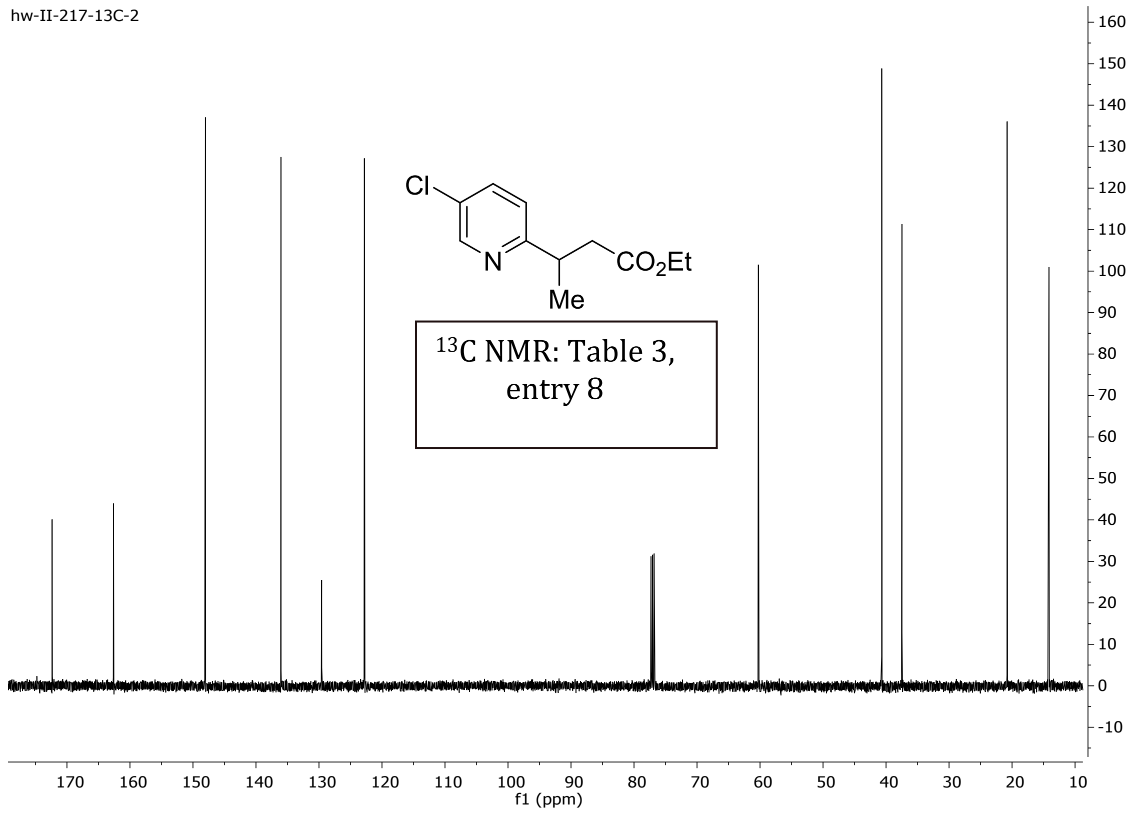
hw-II-217-13C-2
Cl
N
CO2Et
Me
13C NMR: Table 3, entry 8
f1 (ppm)

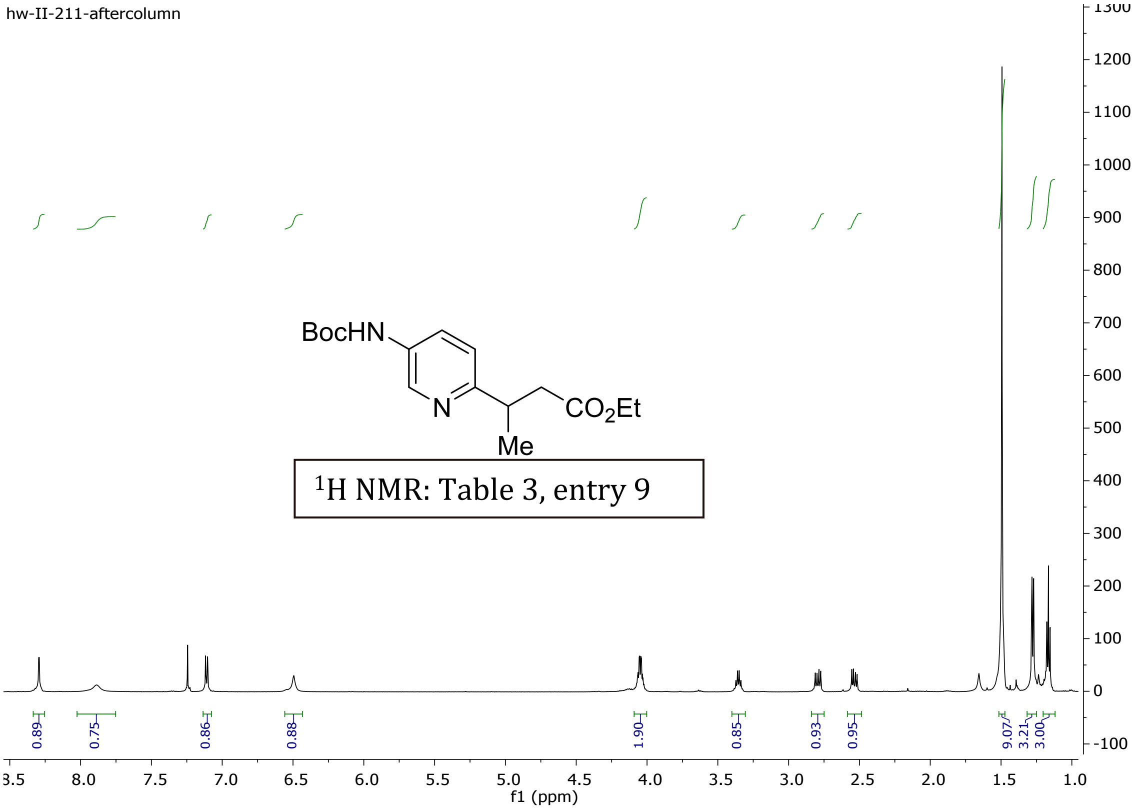
hw-II-211-aftercolumn
BocHN
N
CO2Et
Me
1H NMR: Table 3, entry 9
0.89
0.75
0.86
0.88
1.90
0.85
0.93
0.95
9.07
3.21
3.00
f1 (ppm)

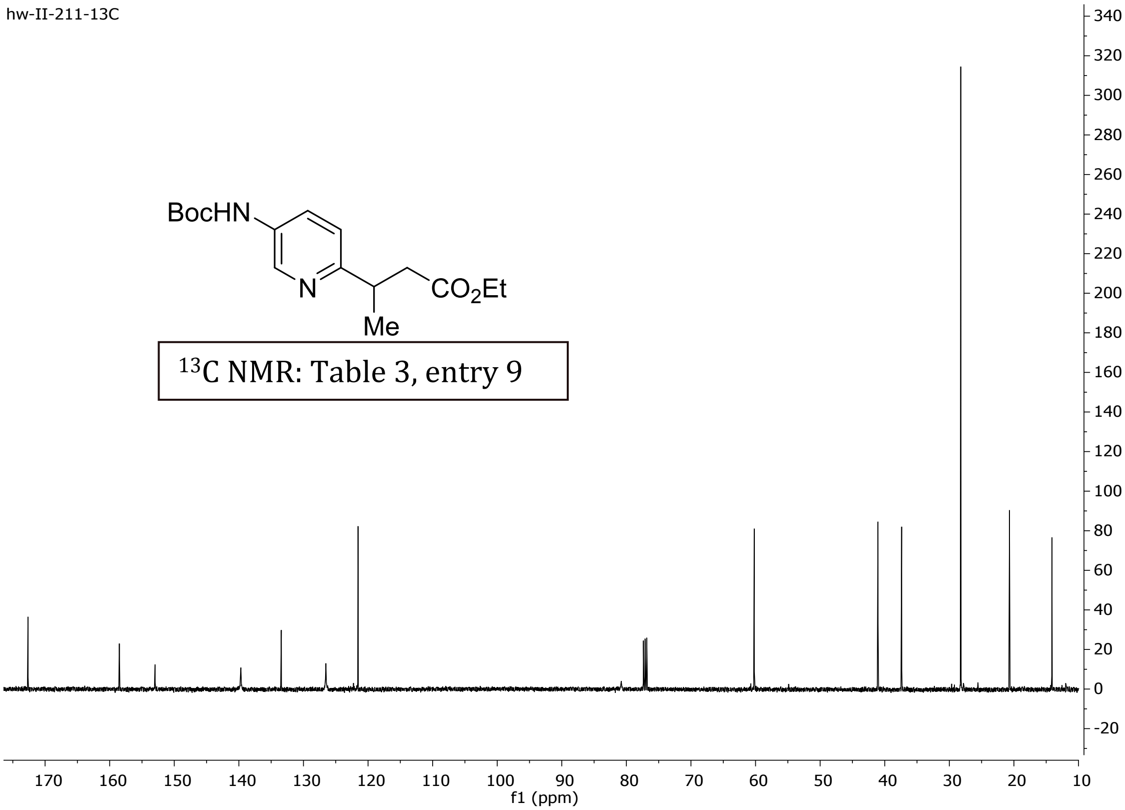
hw-II-211-13C
BocHN
N
CO2Et
Me
13C NMR: Table 3, entry 9
f1 (ppm)

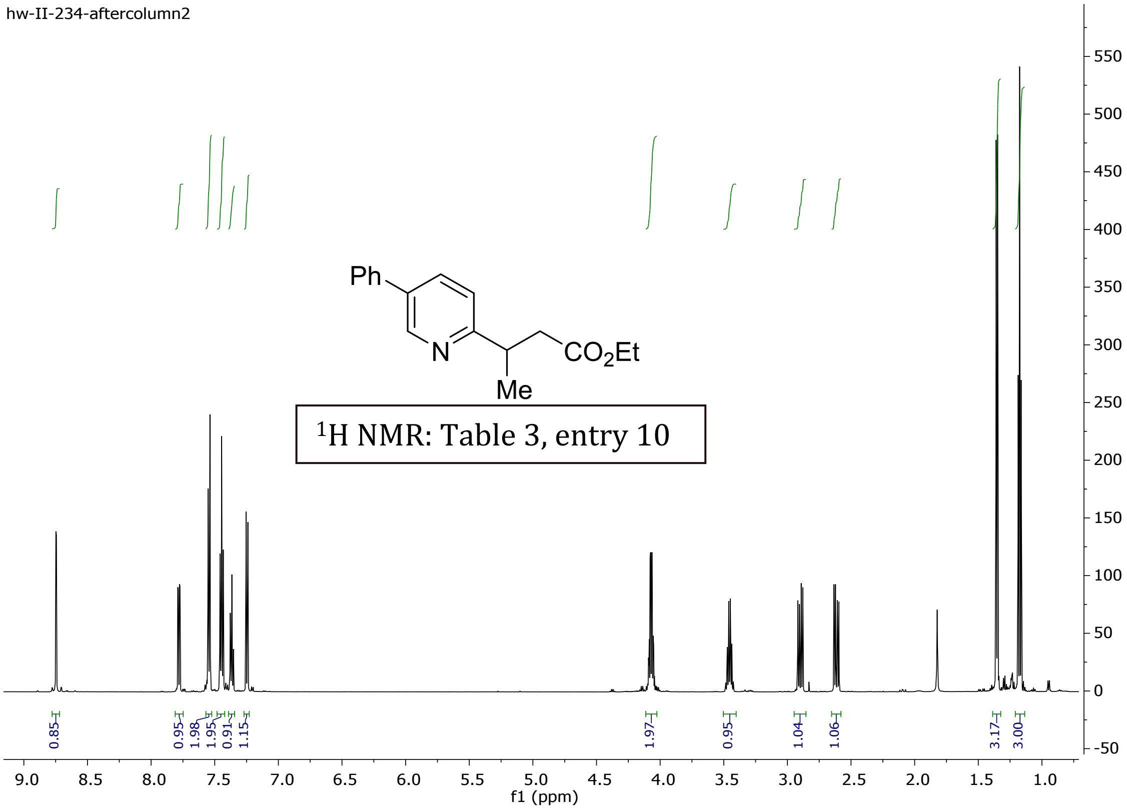
hw-II-234-aftercolumn2
Ph
N
CO2Et
Me
1H NMR: Table 3, entry 10
0.85
0.95
1.98
1.95
0.91
1.15
1.97
0.95
1.04
1.06
3.17
3.00
f1 (ppm)

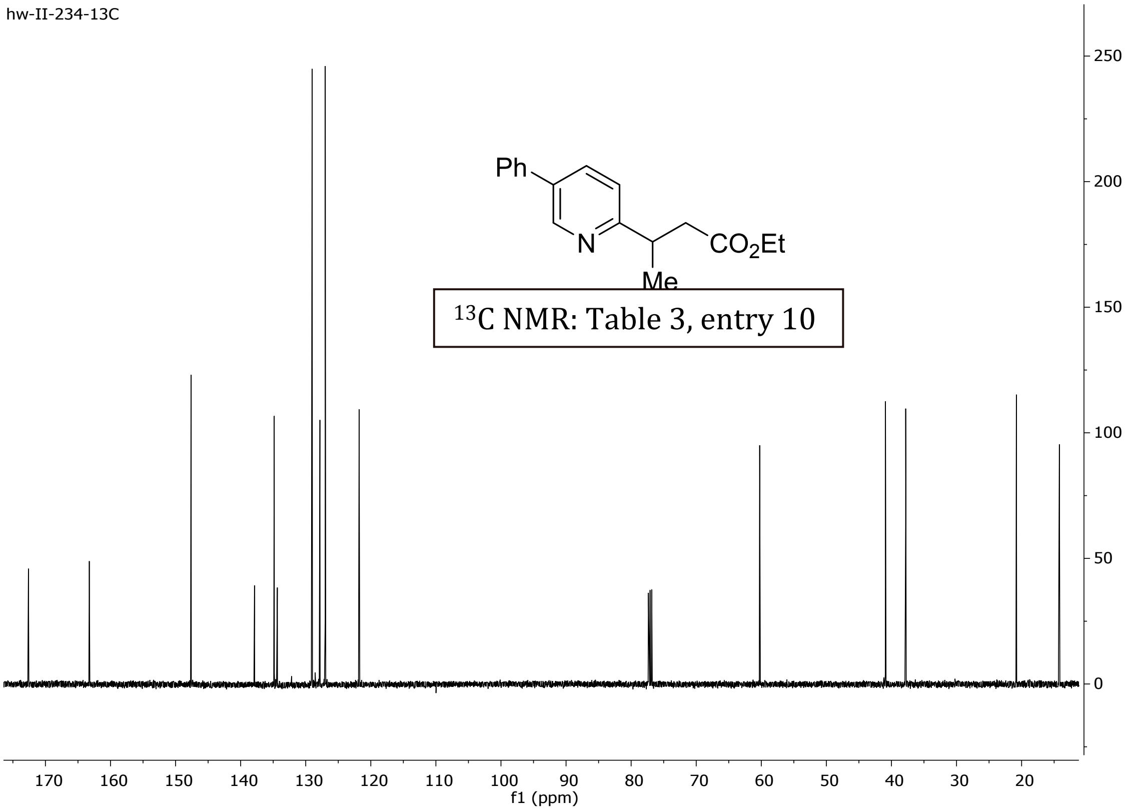
hw-II-234-13C
Ph
N
CO2Et
Me
13C NMR: Table 3, entry 10
f1 (ppm)

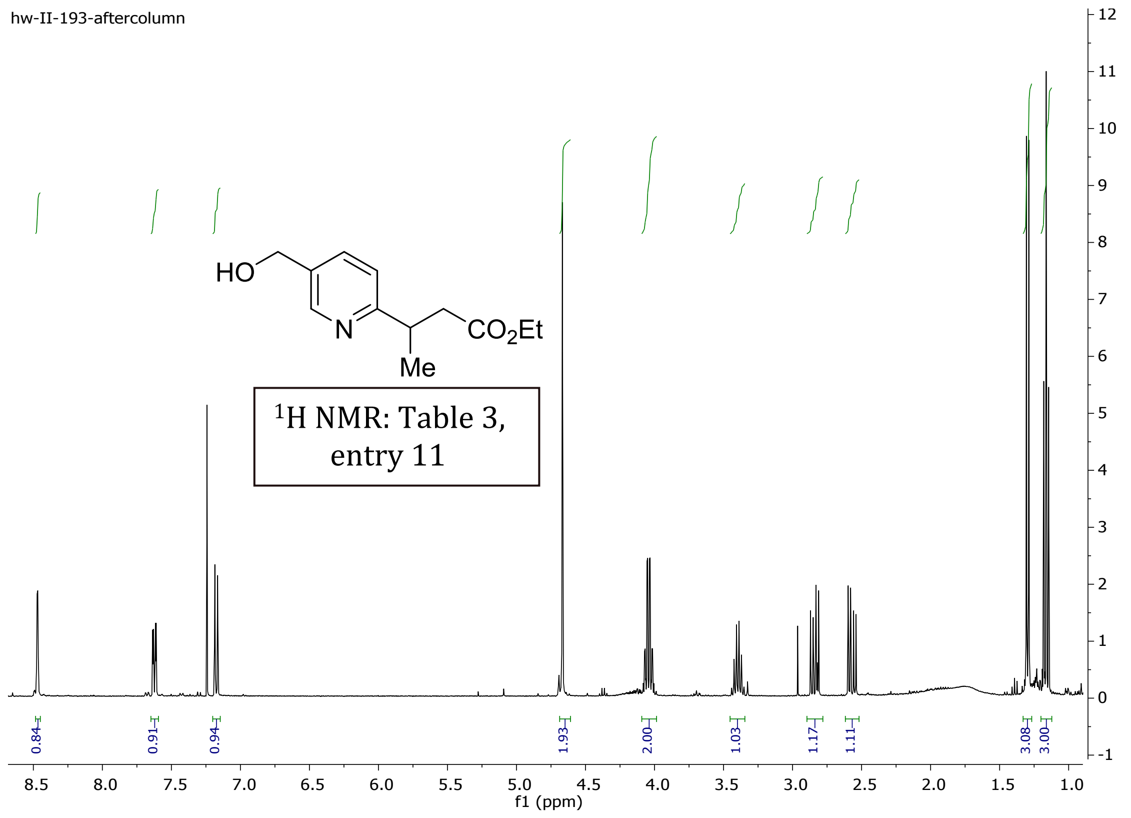
hw-II-193-aftercolumn
HO
N
CO2Et
Me
1H NMR: Table 3, entry 11
f1 (ppm)

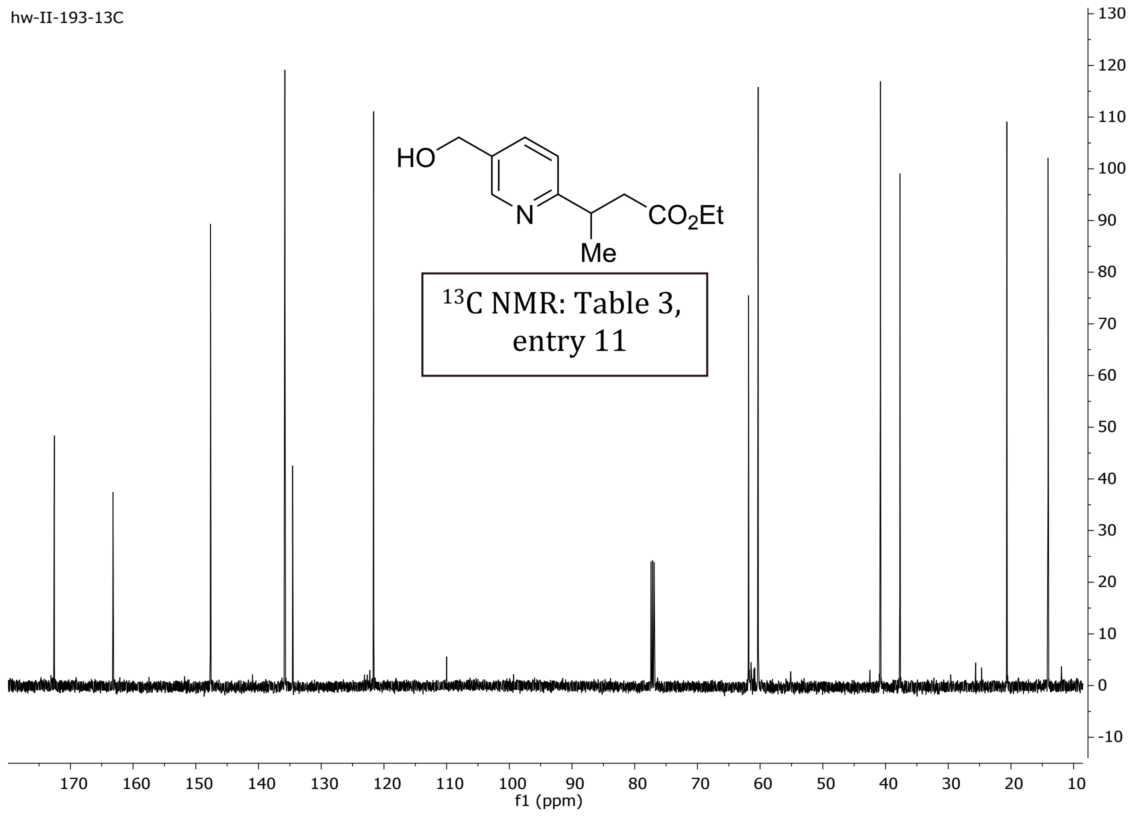
hw-II-193-13C
HO
N
CO2Et
Me
13C NMR: Table 3, entry 11
f1 (ppm)

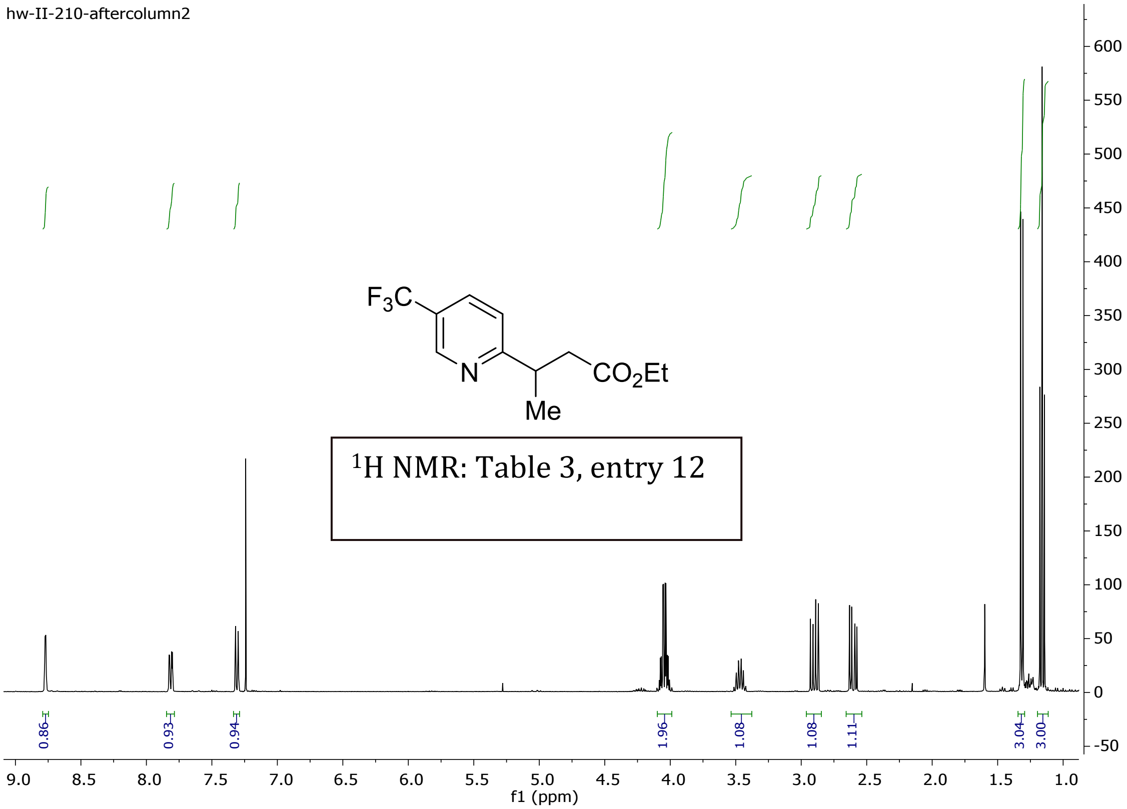
hw-II-210-aftercolumn2
F_3C
N
CO_2Et
Me
^{1}H NMR: Table 3, entry 12
0.86
0.93
0.94
1.96
1.08
1.08
1.11
3.04
3.00
f1 (ppm)

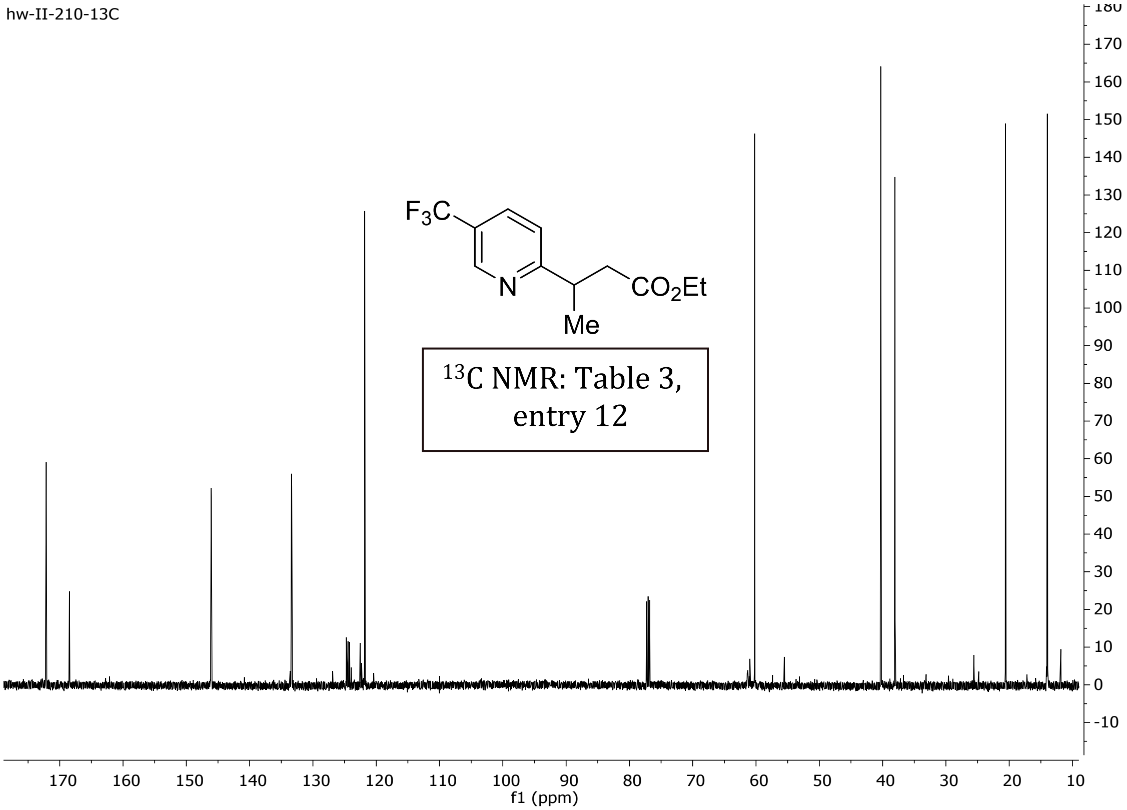
hw-II-210-13C
F_3C
N
CO_2Et
Me
^{13}C NMR: Table 3, entry 12
f1 (ppm)

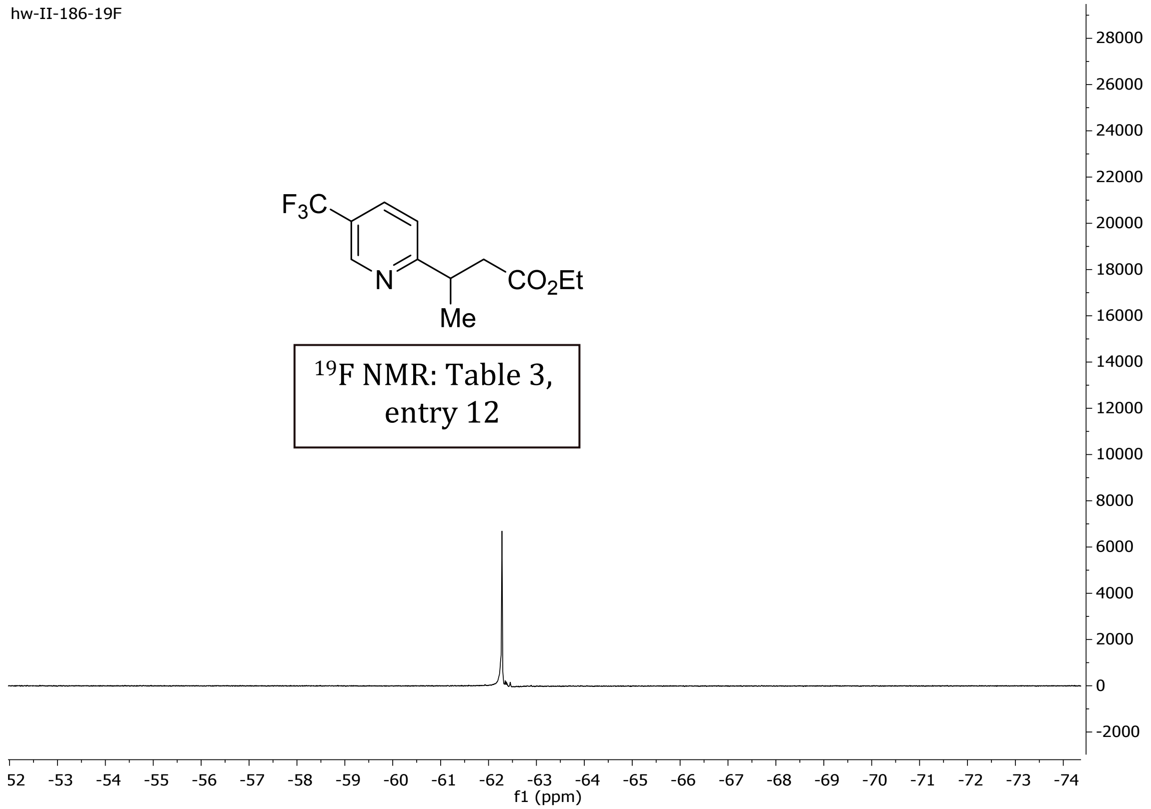
hw-II-186-19F
F_3C
N
CO_2Et
Me
^{19}F NMR: Table 3, entry 12
28000
26000
24000
22000
20000
18000
16000
14000
12000
10000
8000
6000
4000
2000
0
-2000
52 -53 -54 -55 -56 -57 -58 -59 -60 -61 -62 -63 -64 -65 -66 -67 -68 -69 -70 -71 -72 -73 -74
f1 (ppm)

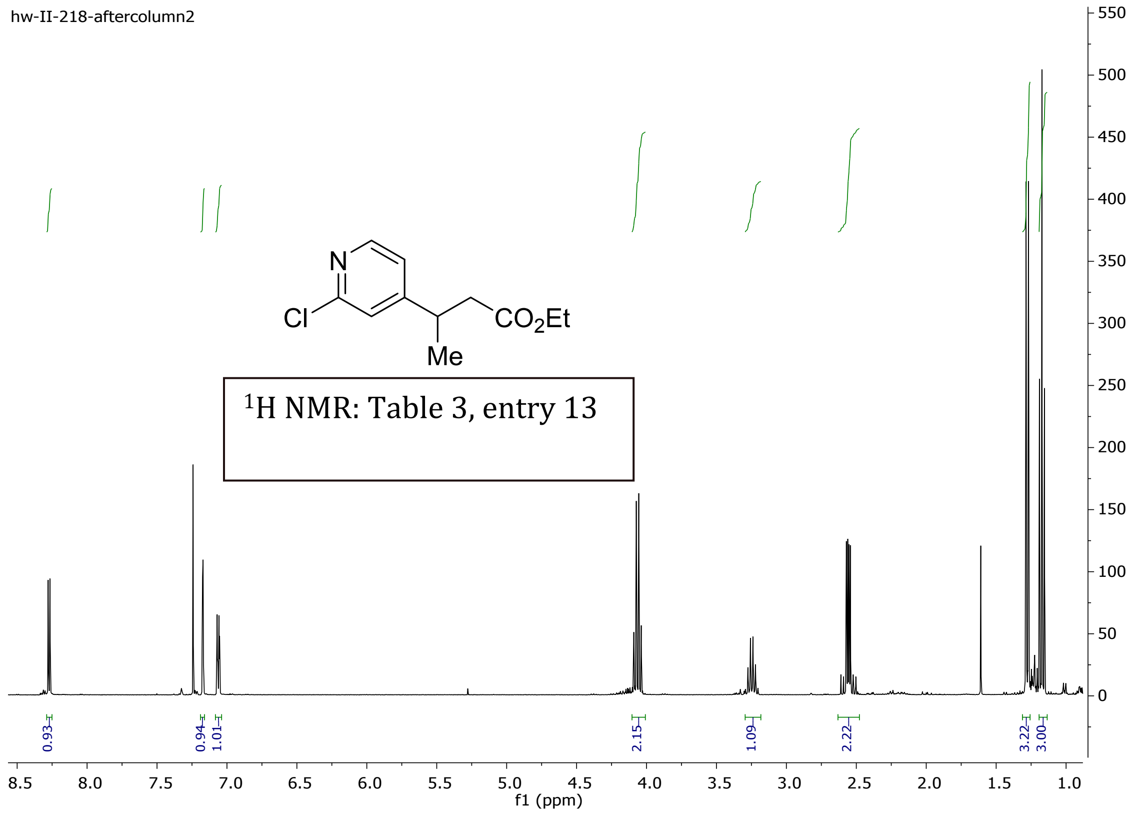
hw-II-218-aftercolumn2
N
Cl
CO_2Et
Me
^{1}H NMR: Table 3, entry 13
0.93
0.94
1.01
2.15
1.09
2.22
3.22
3.00
f1 (ppm)

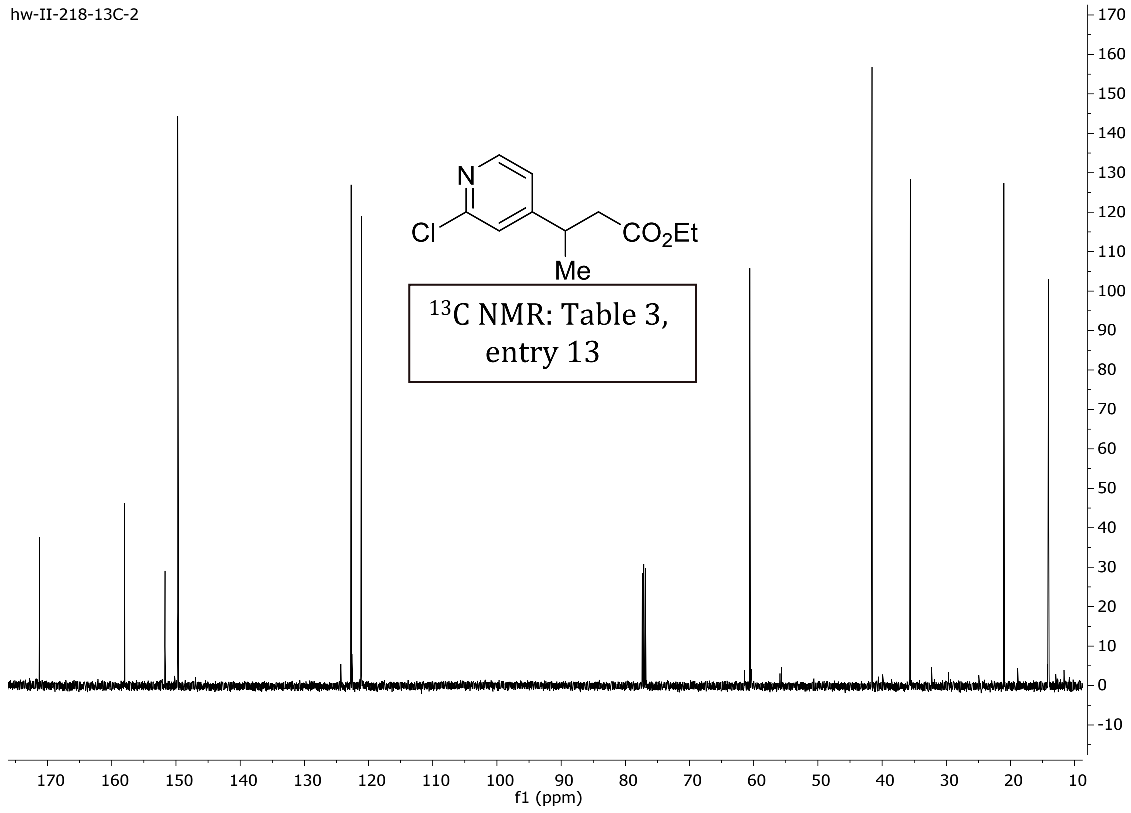
hw-II-218-13C-2
N
Cl
CO_2Et
Me
^{13}C NMR: Table 3, entry 13
f1 (ppm)

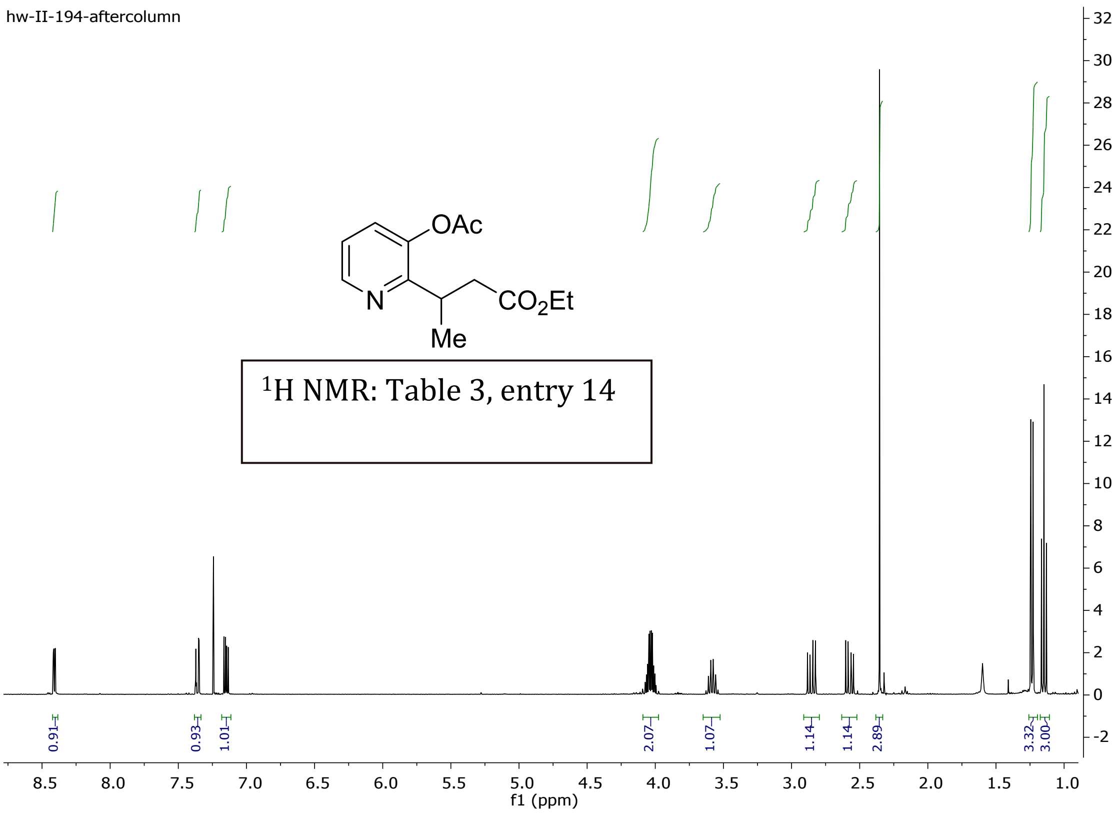
hw-II-194-aftercolumn
OAc
N
CO2Et
Me
1H NMR: Table 3, entry 14
0.91
0.93
1.01
2.07
1.07
1.14
1.14
2.89
3.32
3.00
f1 (ppm)

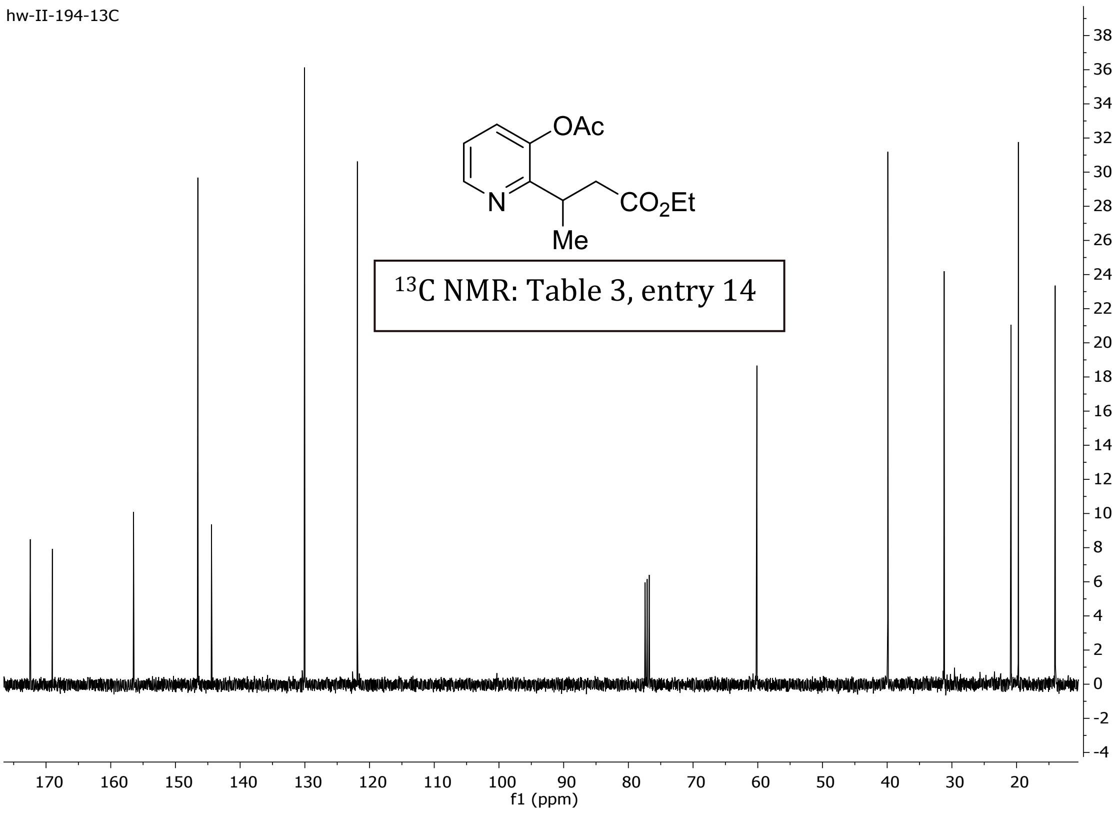
hw-II-194-13C
OAc
N
CO2Et
Me
13C NMR: Table 3, entry 14
f1 (ppm)

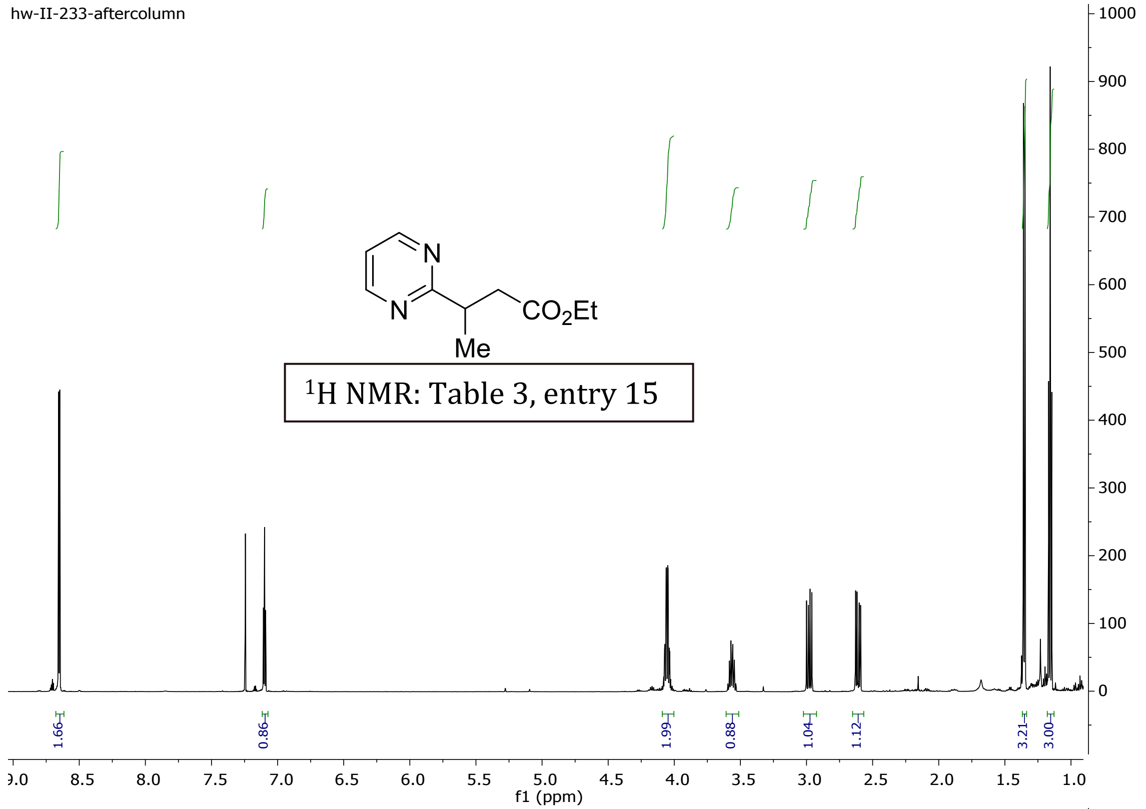
hw-II-233-aftercolumn
N
N
CO2Et
Me
1H NMR: Table 3, entry 15
1.66
0.86
1.99
0.88
1.04
1.12
3.21
3.00
f1 (ppm)

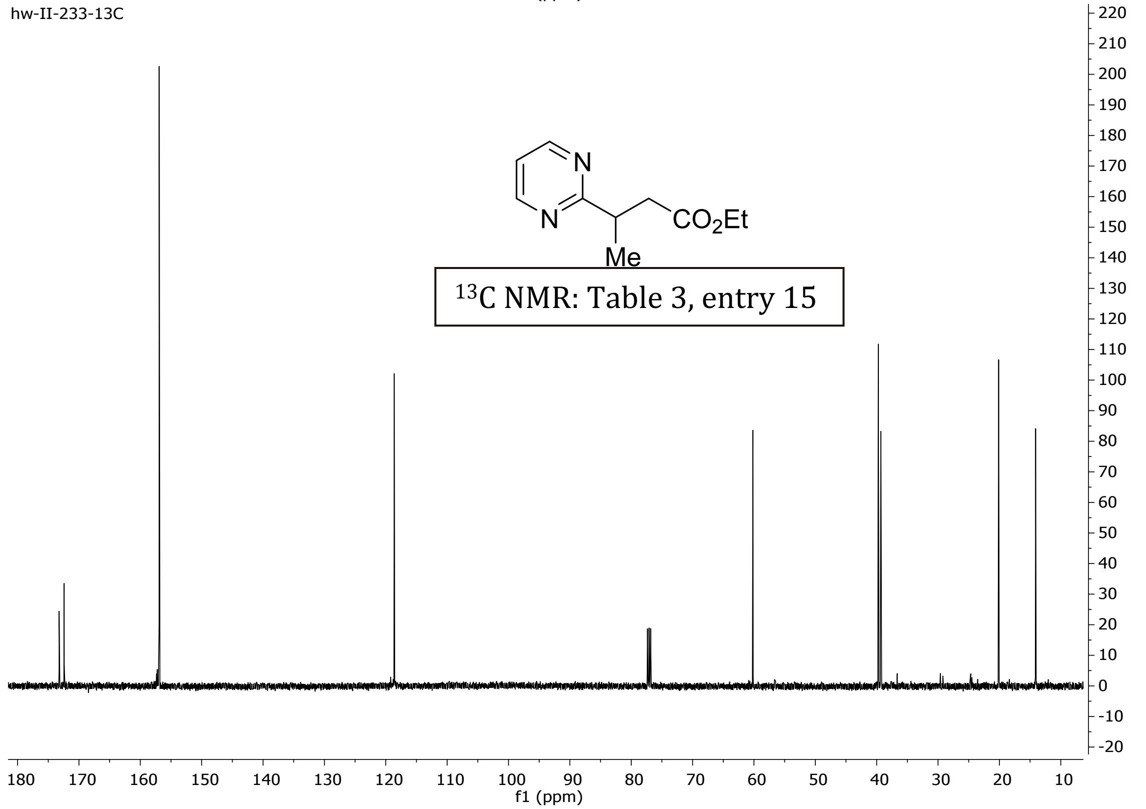
hw-II-233-13C
N
N
CO2Et
Me
13C NMR: Table 3, entry 15
f1 (ppm)

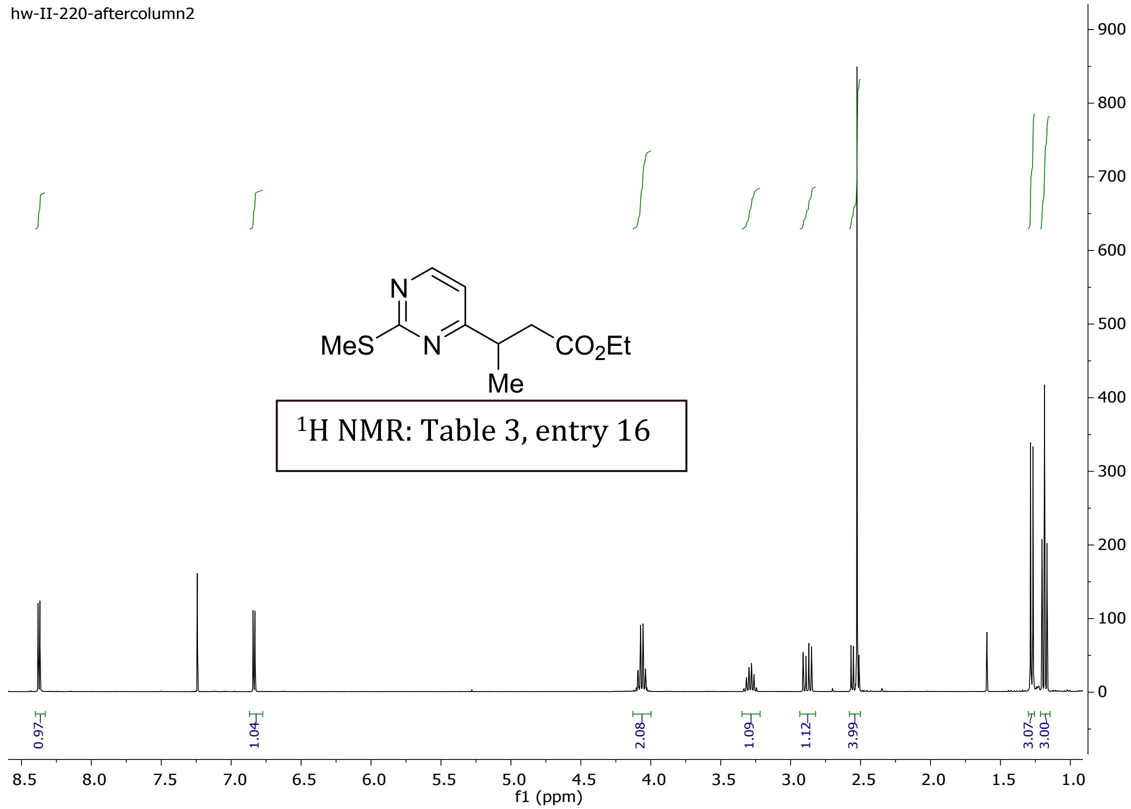
hw-II-220-aftercolumn2
MeS
N
N
CO2Et
Me
1H NMR: Table 3, entry 16
0.97
1.04
2.08
1.09
1.12
3.99
3.07
3.00
f1 (ppm)

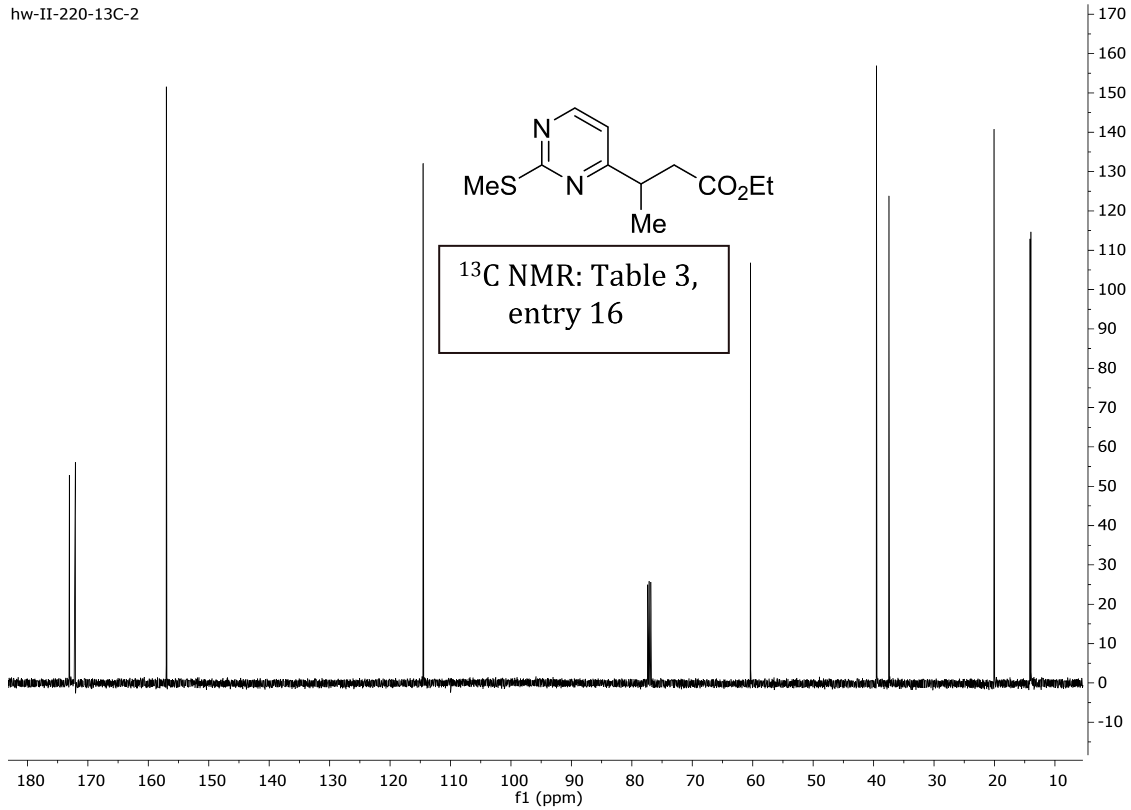
hw-II-220-13C-2
MeS
N
N
CO2Et
Me
13C NMR: Table 3, entry 16
f1 (ppm)

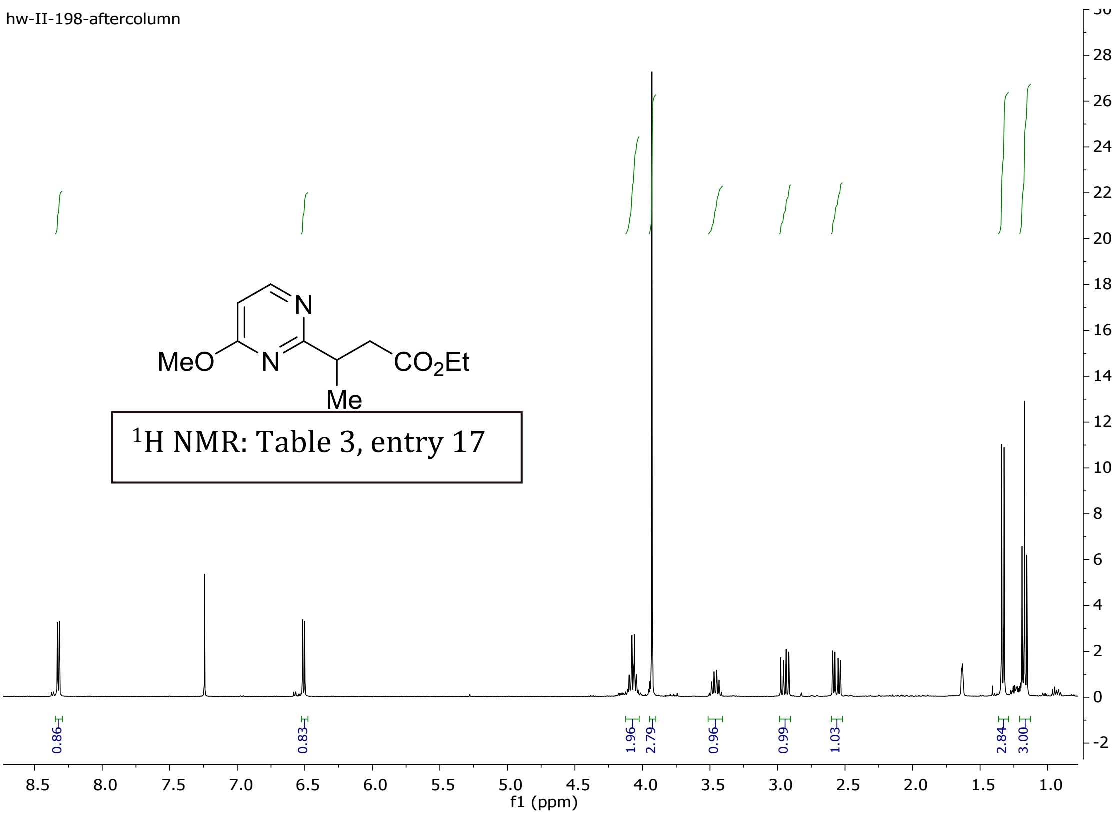
hw-II-198-aftercolumn
MeO
N
N
CO2Et
Me
1H NMR: Table 3, entry 17
0.86
0.83
1.96
2.79
0.96
0.99
1.03
2.84
3.00
f1 (ppm)

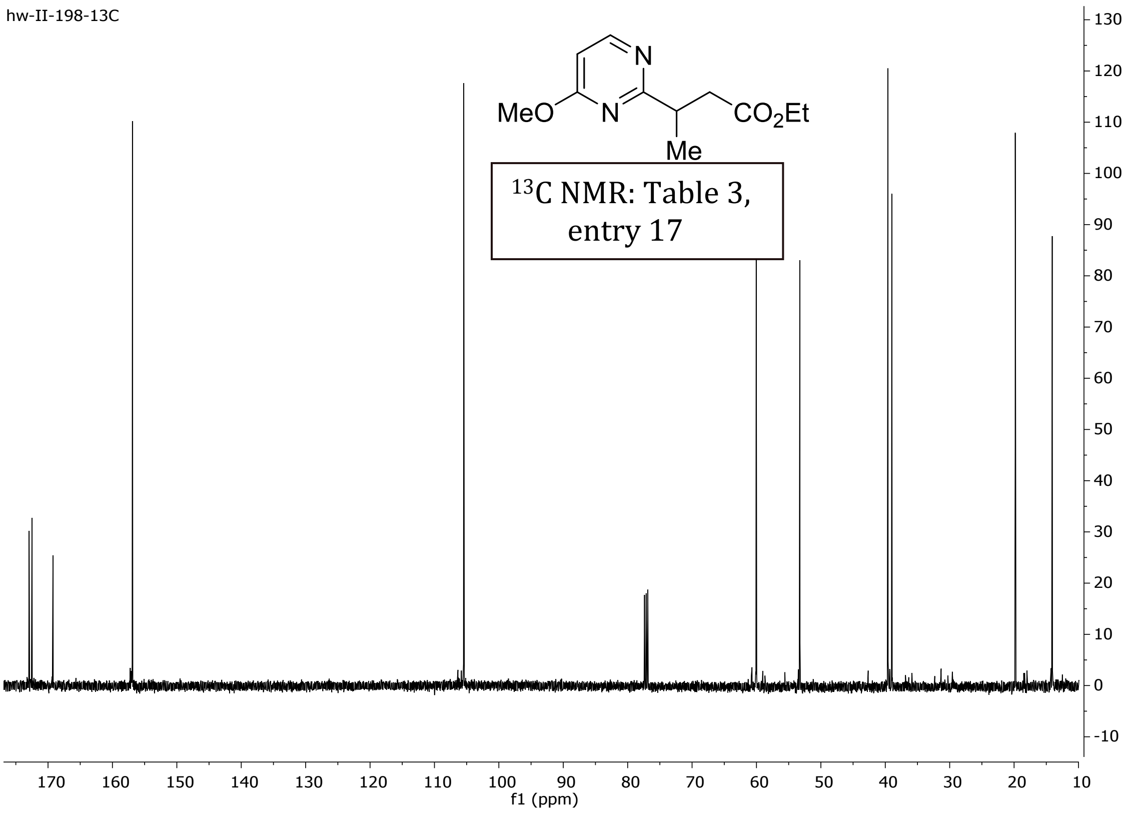
hw-II-198-13C
MeO
N
N
CO2Et
Me
13C NMR: Table 3, entry 17
f1 (ppm)

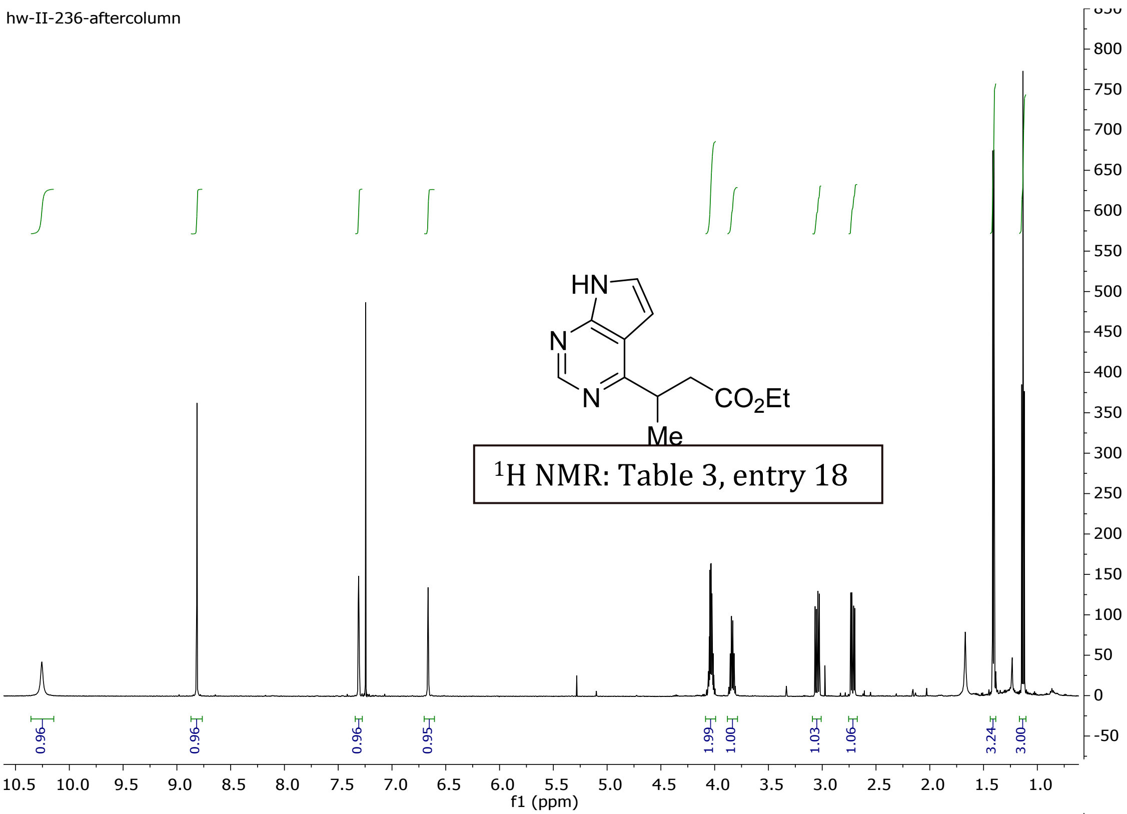
hw-II-236-aftercolumn
HN
N
N
CO_2Et
Me
^{1}H NMR: Table 3, entry 18
0.96
0.96
0.96
0.95
1.99
1.00
1.03
1.06
3.24
3.00
f1 (ppm)

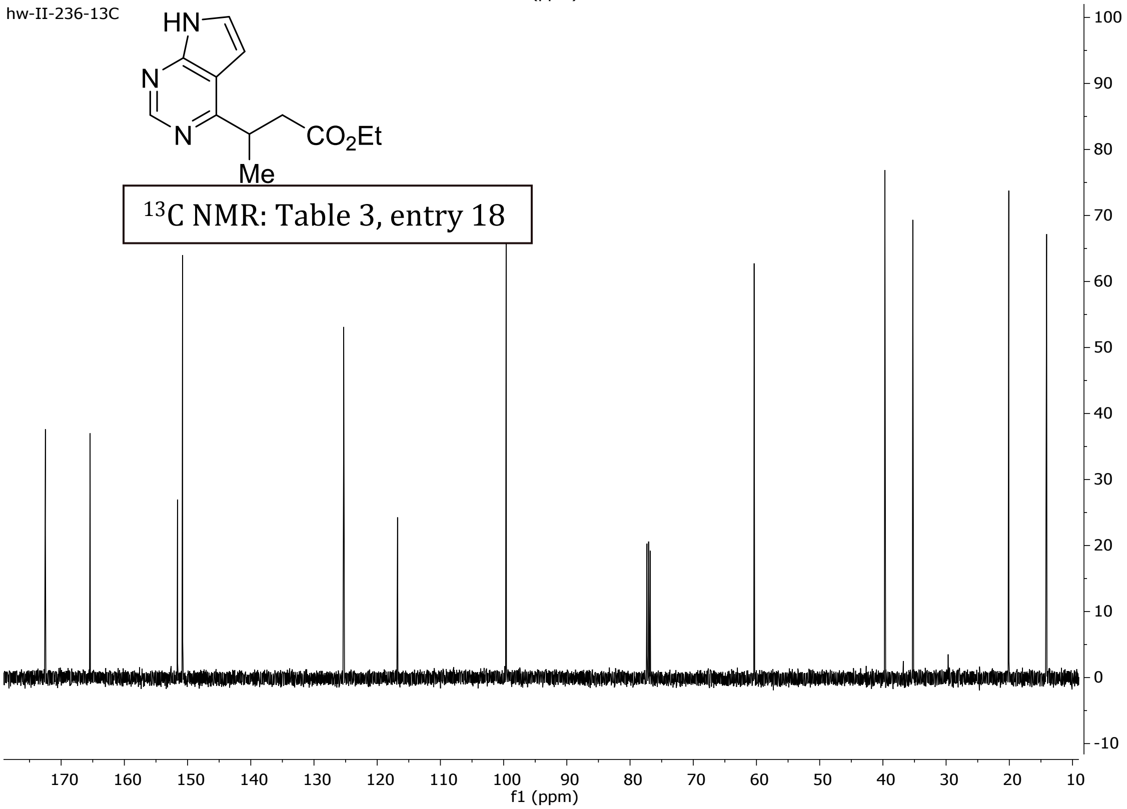
hw-II-236-13C
HN
N
N
CO_2Et
Me
^{13}C NMR: Table 3, entry 18
f1 (ppm)

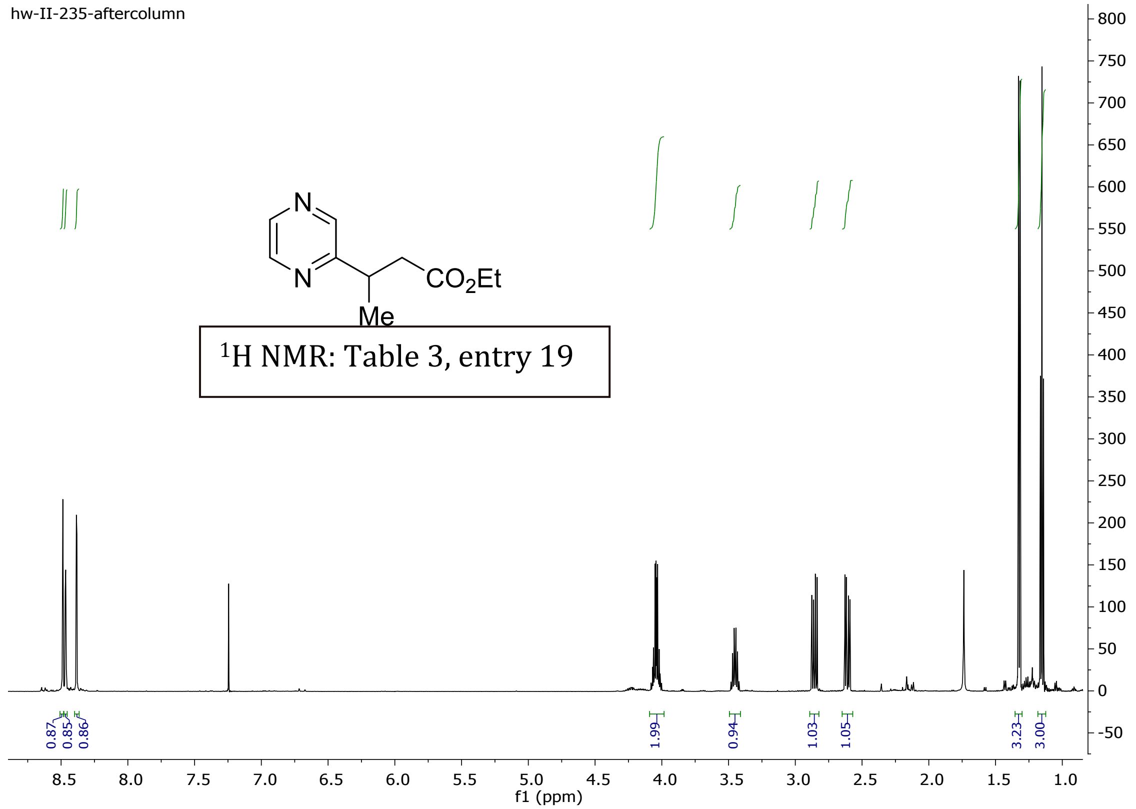
hw-II-235-aftercolumn
N
N
CO2Et
Me
1H NMR: Table 3, entry 19
0.87
0.85
0.86
1.99
0.94
1.03
1.05
3.23
3.00
f1 (ppm)

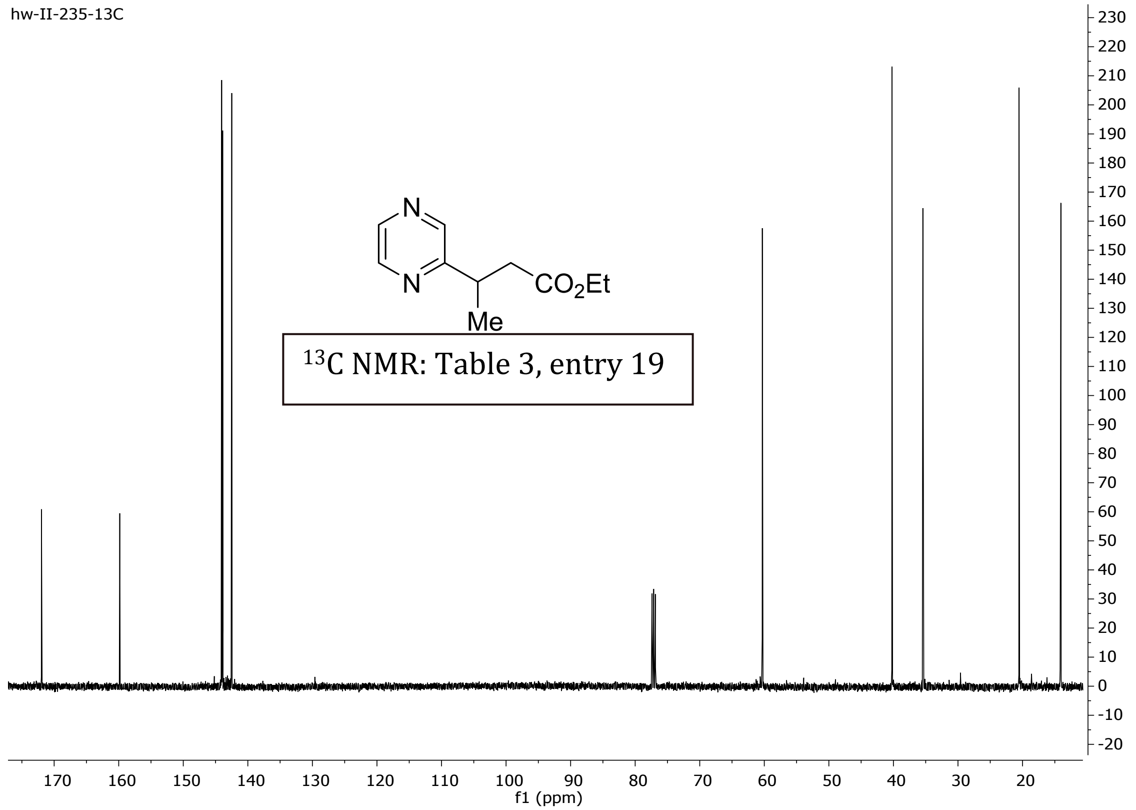
hw-II-235-13C
N
N
CO2Et
Me
13C NMR: Table 3, entry 19
f1 (ppm)

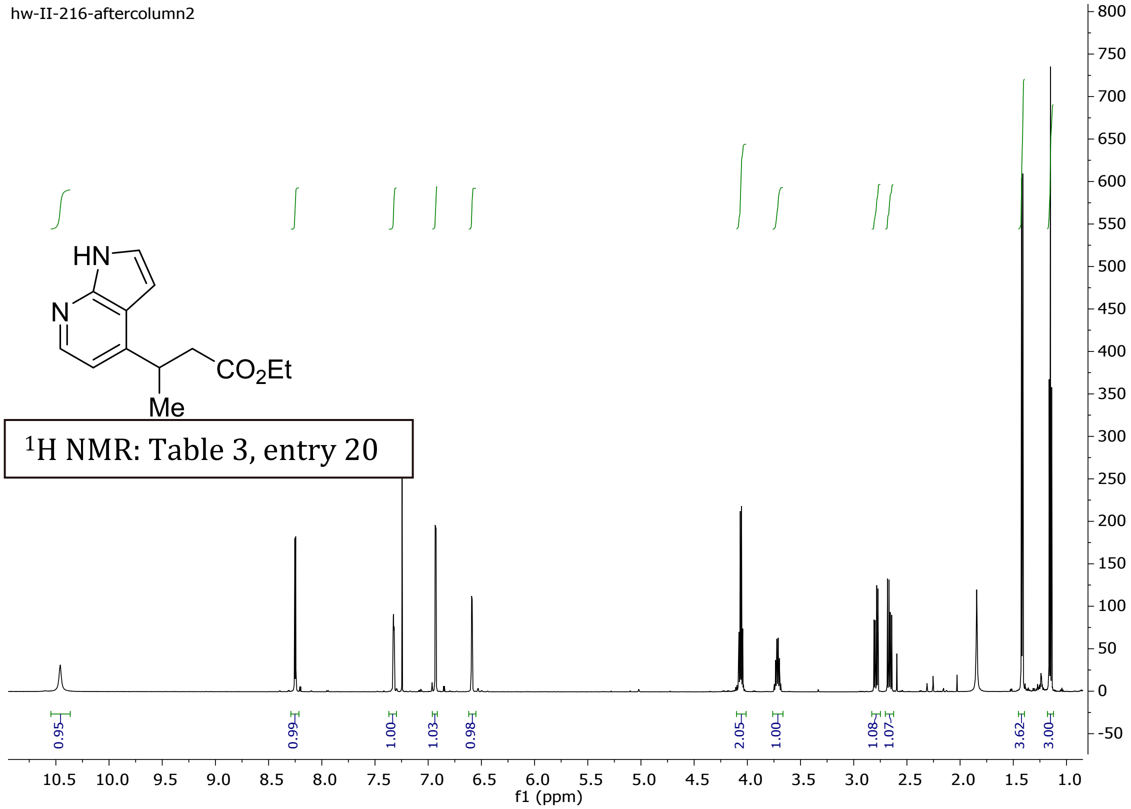
hw-II-216-aftercolumn2
HN
N
CO2Et
Me
1H NMR: Table 3, entry 20
0.95
0.99
1.00
1.03
0.98
2.05
1.00
1.08
1.07
3.62
3.00
f1 (ppm)

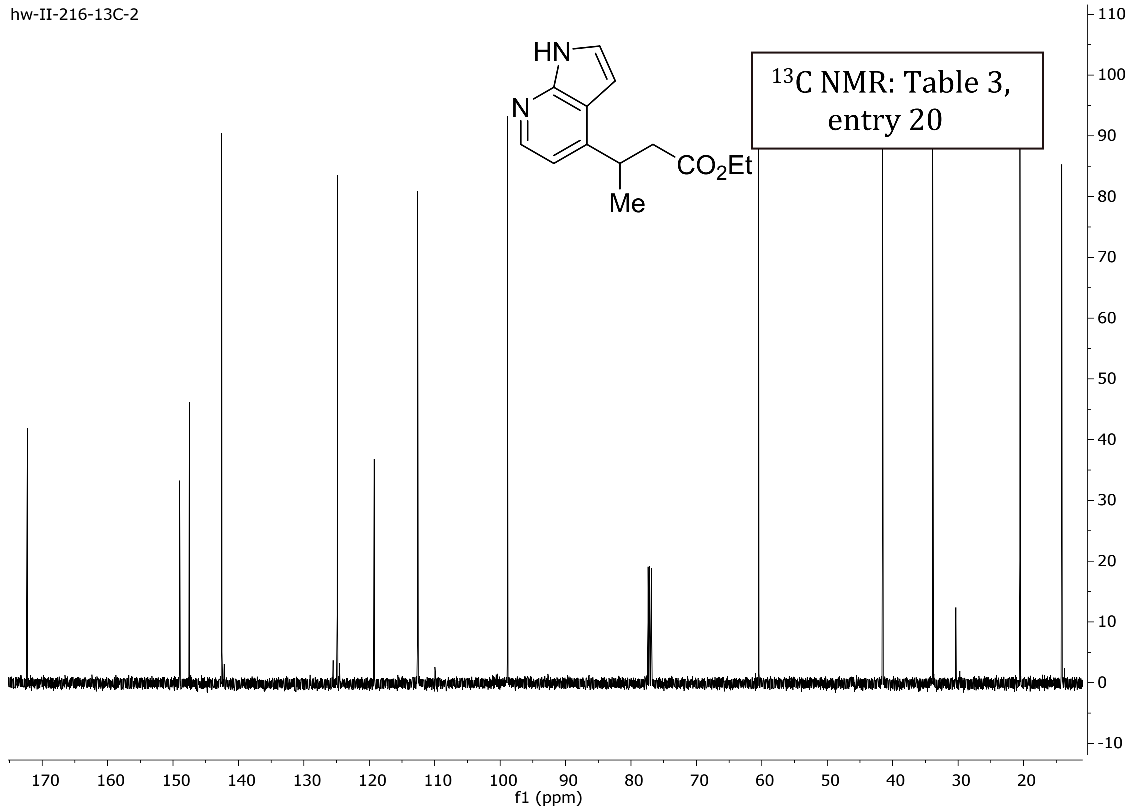
hw-II-216-13C-2
HN
N
CO2Et
Me
13C NMR: Table 3, entry 20
f1 (ppm)

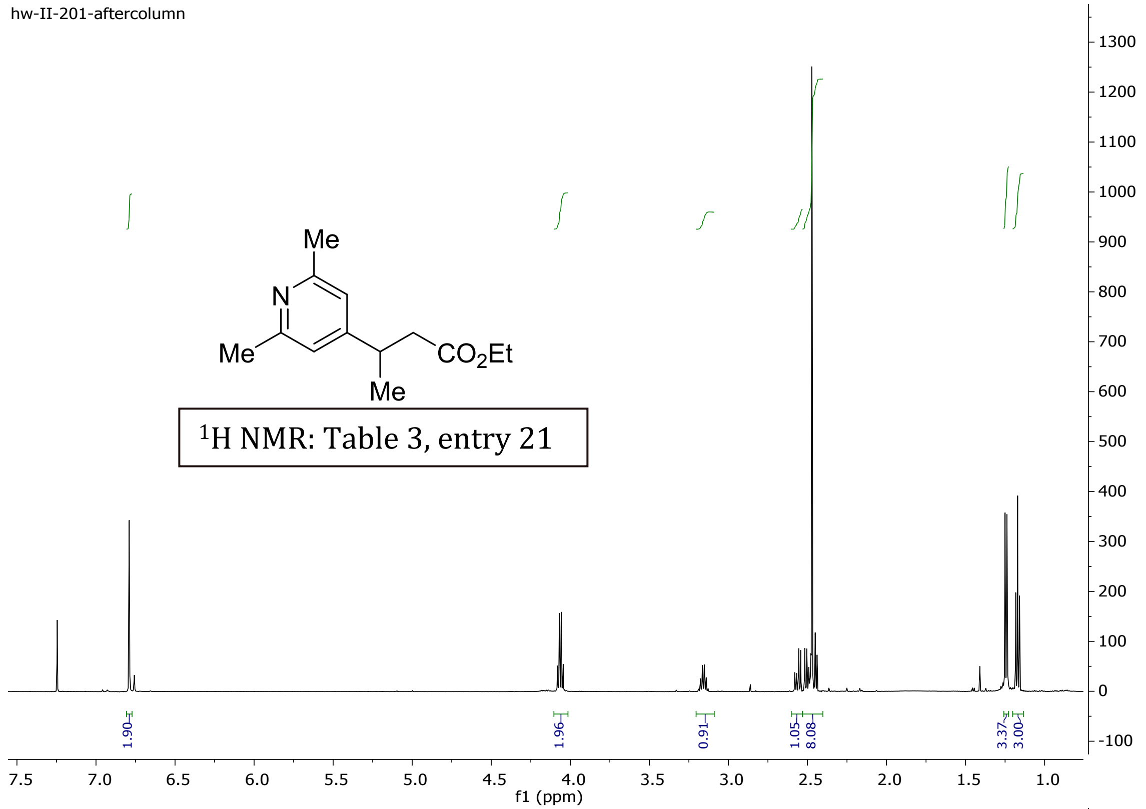
hw-II-201-aftercolumn
Me
N
Me
CO2Et
Me
1H NMR: Table 3, entry 21
1.90
1.96
0.91
1.05
8.08
3.37
3.00
f1 (ppm)

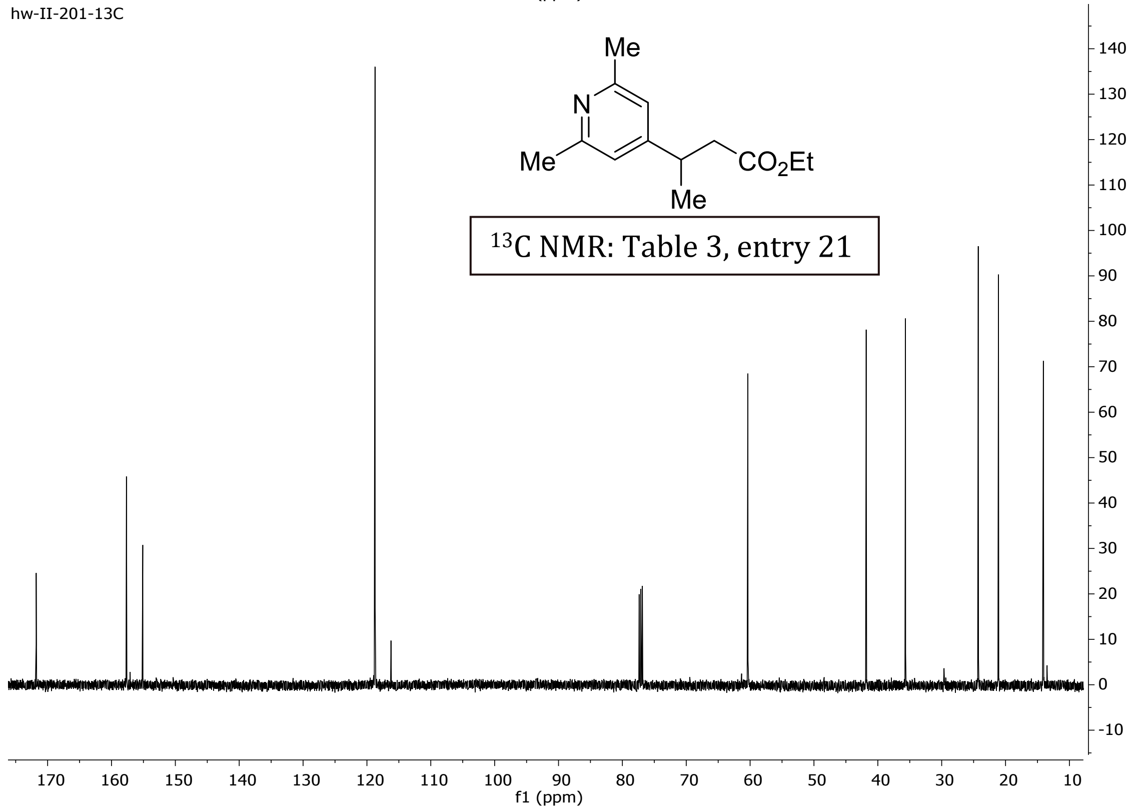
hw-II-201-13C
Me
N
Me
CO2Et
Me
13C NMR: Table 3, entry 21
f1 (ppm)
